# ^31^P NMR Chemical Shift Anisotropy in Paramagnetic Lanthanide Phosphide Complexes

**DOI:** 10.1021/jacsau.4c01018

**Published:** 2025-02-21

**Authors:** Jack Baldwin, Katherine L. Bonham, Toby R. C. Thompson, Gemma K. Gransbury, George F. S. Whitehead, Iñigo J. Vitorica-Yrezabal, Daniel Lee, Nicholas F. Chilton, David P. Mills

**Affiliations:** 1 Department of Chemistry, The University of Manchester, Oxford Road, Manchester M13 9PL, U.K.; 2 Department of Chemical Engineering, The University of Manchester, Oxford Road, Manchester M13 9PL, U.K.; 3 Research School of Chemistry, The Australian National University, Sullivans Creek Road, Canberra ACT2601, Australia

**Keywords:** lanthanide, phosphide, paramagnetic, NMR spectroscopy, magic angle spinning, *ab initio*, DFT calculations

## Abstract

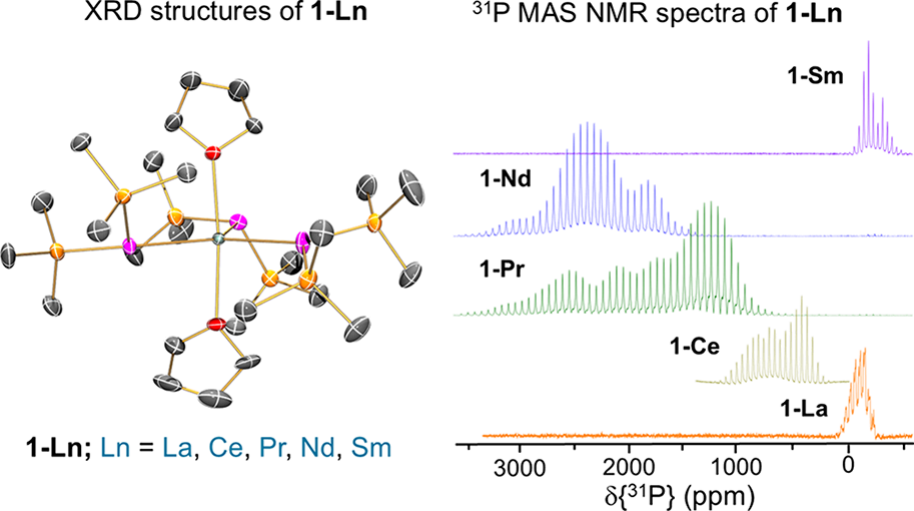

Lanthanide (Ln) magnetic resonance imaging and chiral shift reagents generally exploit ^1^H NMR shifts, as paramagnetic broadening tends to preclude the use of heavier, less sensitive nuclei. Here, we report the solution and solid-state ^31^P NMR shifts of an isostructural series of distorted trigonal bipyramidal Ln(III) *tris*-silylphosphide complexes, [Ln{P(SiMe_3_)_2_}_3_(THF)_2_] (**1-Ln**; Ln = La, Ce, Pr, Nd, Sm); **1-Ln** was also characterized by elemental analysis; single-crystal and powder X-ray diffraction; multinuclear NMR, EPR, ATR-IR, and UV–vis-NIR spectroscopy; and SQUID magnetometry. Breaking assumptions, we observed paramagnetically broadened ^31^P NMR spectra for the Ln-bound P atoms for the **1-Ln** family; in solution, **1-Nd** showed the most downfield chemical shift (δ{^31^P} = 2570.14 ppm) and **1-Sm** the most upfield value (δ{^31^P} = −259.21 ppm). We determined the span of the chemical shift anisotropies (CSAs) for solid **1-Ln** using magic angle spinning NMR spectroscopy; the CSA was largest for **1-Pr** (**Ω**{^31^P} ≈ 2000 ppm), consistent with a combination of paramagnetism and the relatively large differences in pyramidalization of the three P atoms in the solid-state. Density functional theory calculations for **1-La** were in excellent agreement with the experimentally determined ^31^P NMR parameters. We find good agreement of experimental ^1^H NMR chemical shifts with *ab initio*-calculated values for paramagnetic **1-Ln**, while the shifts of heavier ^13^C, ^29^Si, and ^31^P nuclei are not well-reproduced due to the current limitations of paramagnetic NMR calculations for nuclei with large contact shifts.

## Introduction

The unique magnetic and luminescent behavior of lanthanide (Ln) complexes^1^ has been exploited in emissive probes,^2−10^ magnetic resonance imaging PARASHIFT tags,^11−13^ chiral shift reagents,^14−16^ and the determination of spin–spin coupling between metal ions.^17−19^ Magnetic properties may be probed by nuclear magnetic resonance (NMR) spectroscopy, which can also provide information on sample purity, thermodynamic and kinetic parameters, dynamic processes, and exchange coupling.^20^ Signals in the NMR spectra of most Ln complexes typically exhibit low resolutions and large paramagnetic chemical shifts due to nuclear hyperfine interactions with unpaired 4f electrons.^21−23^ As ^1^H nuclei have the highest sensitivity, the ^1^H NMR spectra of paramagnetic Ln complexes can often be fully assigned and correlated to benchmark electronic structures,^21^ but spectra are often intractable for less receptive NMR-active nuclei and attempts to calculate chemical shifts are scarce.^24,25^

Solid-state (ss) NMR allows the chemical shift anisotropy (CSA) to be determined alongside the isotropic chemical shift. The chemical shift tensor due to unpaired electrons **δ**^unpaired^ is described in the most general sense by the theory of van den Heuvel and Soncini,^26^ but can be more intuitively understood by approximating it in terms of the magnetic susceptibility tensor **χ** and reduced hyperfine coupling tensor **C** (eq 1):^23^
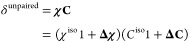
1where each tensor can be separated into isotropic (e.g., χ^iso^ = tr(**χ**)/3) and anisotropic (e.g., **Δχ**, which is a symmetric tensor) components. *C*^iso^ can be approximated as arising from through-bond Fermi contact interactions and **ΔC** due to the through-space spin-dipolar interactions. In the presence of strong spin–orbit coupling, this separation can break down, and additional contributions to **C**, including that due to the paramagnetic spin–orbit (PSO) interaction, can also be significant. These lead to **C** containing an antisymmetric anisotropic component in addition to the isotropic and symmetric anisotropic parts.^27^ Within this simplified formulation, the isotropic component of **δ**^unpaired^ can be seen to consist of χ^iso^*C*^iso^ (the contact shift, CS) and tr(**ΔχΔC**)/3 (the pseudocontact shift, PCS), the latter of which vanishes in the spin-only limit where anisotropy and hence **Δχ =** 0. The anisotropic part of **δ**^unpaired^, the CSA, contains contributions from both the Fermi contact interaction (*C*^iso^**Δχ**) and the spin-dipolar interaction (χ^iso^**ΔC** and the anisotropic part of **ΔχΔC**). Thus, both the isotropic shift and CSA inform on the isotropic and anisotropic magnetic properties of a paramagnetic metal ion, as well as on the bonding and spatial arrangement of the complex containing it.^28^

Ln amide (NR_2_) chemistry is mature,^29−31^ but the corresponding phosphide (PR_2_) chemistry is underdeveloped^32,33^ due to the preference for hard Lewis acidic Ln ions to bind with charge-dense Lewis basic ligands.^1^ This is exemplified by Ln bis(trimethylsilyl)amide ({N(SiMe_3_)_2_}) chemistry, which has been burgeoned^31^ since the landmark trigonal pyramidal Ln(III) complexes [Ln{N(SiMe_3_)_2_}_3_] were reported by Bradley in the early 1970s,^34^ while there are only a limited number of structurally authenticated group 3 and f-block metal bis(trimethylsilyl)phosphide ({P(SiMe_3_)_2_}) complexes: [Sc{C(PPh_2_S)_2_}{P(SiMe_3_)_2_}(py)_2_] (py = pyridine),^35^ [Y{P(SiMe_3_)_2_}_2_{μ-P(SiMe_3_)_2_}]_2_,^36^ [Ln{P(SiMe_3_)_2_}_3_(THF)_2_] (Ln = Tm,^37^ Nd^38^), [Sm{P(SiMe_3_)_2_}{μ-P(SiMe_3_)_2_}_3_Sm(THF)_3_],^39^ [{Ln[P(SiMe_3_)_2_]_3_(THF)}_2_(μ-I)K_3_(THF)] (Ln = Sm, Eu),^40^ [KYb{P(SiMe_3_)_2_}_3_{μ-K[P(SiMe_3_)_2_]}_2_]_∞_,^40^*trans*-[Ln{P(SiMe_3_)_2_}_2_(py)_4_] (Ln = Sm, Eu, Yb),^40^ [Ln{P(SiMe_3_)_2_}_2_(18-crown-6)] (Ln = Sm, Eu, Yb),^40^ [An{P(SiMe_3_)_2_}(Cp*)_2_(Cl)] (An = Th, U; Cp* = C_5_Me_5_),^41^ [An(Tren^R^){P(SiMe_3_)_2_}] (An = Th, U; Tren^R^ = N(CH_2_CH_2_NSiR_3_)_3_; SiR_3_ = DMBS, SiMe_2_^t^Bu or TIPS, Si^i^Pr_3_).^42^

In a recent^29^ Si paramagnetic NMR (pNMR) study of a family of locally *D*_3*h*_-symmetric early f-block M(III) silanide complexes, [M{Si(SiMe_3_)_3_}_3_(THF)_2_] (M = La, Ce, Pr, Nd, U), the metal-bound Si atoms were not observed in ^29^Si ssNMR spectra for paramagnetic examples due to line-broadening, and dynamic THF equilibria in the solution complicated the interpretation of solution ^29^Si DEPT90 NMR shifts.^25^ Here, we present a ^31^P NMR study of a structurally analogous series of distorted trigonal bipyramidal Ln(III) phosphide complexes, [Ln{P(SiMe_3_)_2_}_3_(THF)_2_] (**1-Ln**; Ln = La, Ce, Pr, Nd, Sm). Contravening previous assumptions,^38^ we observe ^31^P NMR signals for all **1-Ln** in the ss, allowing us to determine the CSA using a magic angle spinning (MAS) approach. Furthermore, **1-Ln** does not exhibit dynamic structural behavior in solutions, allowing correlation of ^31^P solution and ssNMR data. Complexes **1-Ln** were additionally characterized by elemental analysis, single-crystal X-ray diffraction, multinuclear NMR, electron paramagnetic resonance (EPR), ATR-IR, and UV–vis-NIR spectroscopy, SQUID magnetometry, density functional theory (DFT), and complete active space self-consistent field spin–orbit (CASSCF-SO) calculations. We find excellent agreement of experimental data with DFT-computed NMR parameters of ^31^P nuclei for **1-La**, when local dynamics are accounted for, while CASSCF-SO-calculated NMR chemical shifts only showed good agreement with experimental values for ^1^H nuclei for paramagnetic **1-Ln**; ^13^C, ^29^Si, and ^31^P resonances are not well-reproduced due to the current limitations of pNMR shift calculations for nuclei with larger contact shifts.

## Results and Discussion

### Synthesis

**Scheme 1:**
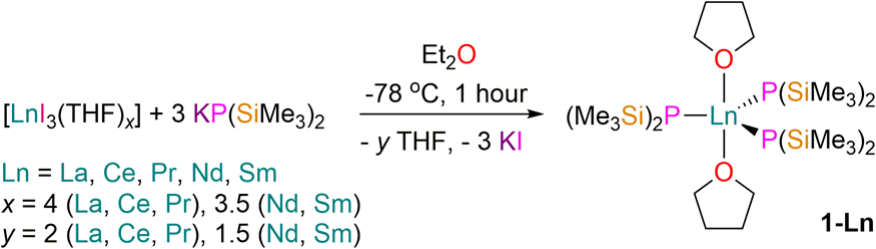
Synthesis of **1-Ln**

Complexes **1-Ln** were prepared by adapting literature procedures^25,38^ for the salt metathesis reactions of [LnI_3_(THF)*_*x*_*] (Ln = La, Ce, Pr, *x* = 4; Ln = Nd, Sm, *x* = 3.5)^43^ with 3 eq. KP(SiMe_3_)_2_^44^ in diethyl ether (Scheme 1). We were not able to isolate significant quantities of **1-Ln** by following the previously reported conditions for the synthesis of **1-Nd** using THF as the reaction solvent.^38^ Upon noting that reaction mixtures tended to darken over time at room temperature, we hypothesized that decomposition pathways involving THF (e.g. ring opening) were occurring. We therefore changed the reaction solvent to diethyl ether and maintained reaction mixtures at −78 °C for 1 h before briefly allowing them to return to room temperature with stirring; all volatiles were then removed under vacuum and products were extracted into hexane. Filtration and concentration of hexane extracts and storage at −30 °C overnight reproducibly gave **1-Ln** in low crystalline yields (14–50%) under these optimized conditions. Overlapping absorption features were observed in the ATR-IR spectra of microcrystalline **1-Ln** (see Supporting Information Figures S1–S6), indicating that they exhibit similar bulk structural features. Elemental analyses performed on **1-Ln** reproducibly gave carbon values that were lower than those predicted. This observation was previously made for **1-Nd** even when combustion agents were added and was attributed to carbide formation.^38^ While this could be interpreted as an intrinsic feature for the **1-Ln** family, we note that low carbon values were also obtained for [M{Si(SiMe_3_)_3_}_3_(THF)_2_],^25^ and this can be a general feature depending on the experimental setup.^45^ However, all other analytical data collected for **1-Ln** are in accordance with their bulk purities (see below).

### X-Ray Crystallography

**Figure 1:**
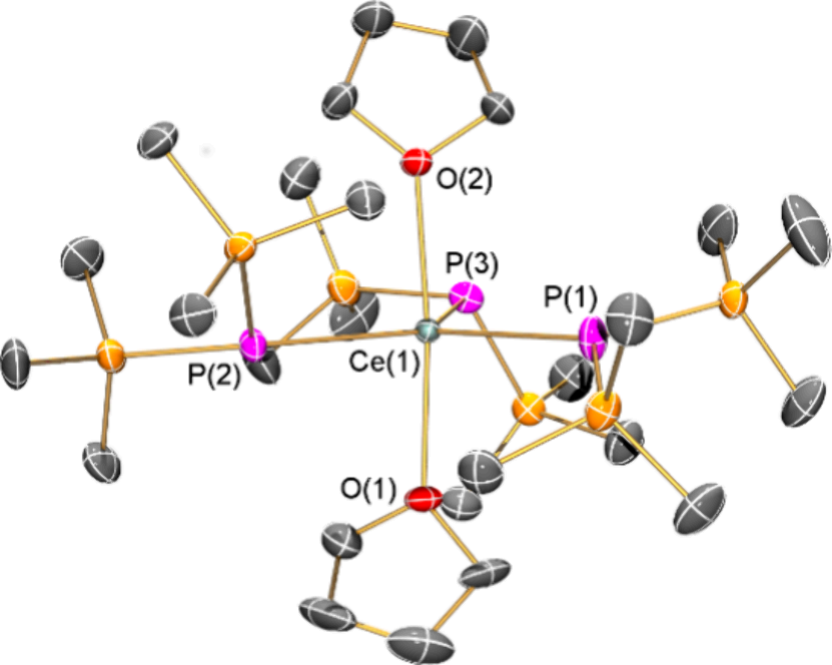
Solid-state structure of **1-Ce**. Displacement ellipsoids set at 50% probability level; hydrogen atoms omitted for clarity.

Single-crystal XRD studies were performed on **1-Ln**; as these complexes show similar structural features, only **1-Ce** is depicted in Figure 1 (see Table 1 for selected bond lengths and angles and Supporting Information Figures S7–S10 and Tables S1–S3 for other structures and crystallographic parameters). We provide an improved data set for **1-Nd**, which is similar to the previously reported structure but with more precise metrical parameters.^38^ Powder XRD studies performed on **1-Ln** were in accord with samples showing bulk phase purities (See Supporting Information Figures S11–S20 and Table S4).

**Table 1:** Selected Mean Bond Lengths and Angles for **1-Ln**

**parameter**	**1-La**	**1-Ce**	**1-Pr**	**1-Nd**	**1-Sm**
**Ln–P/Å**	2.886(2)	2.849(3)	2.837(3)	2.818(2)	2.789(3)
**Ln–O/Å**	2.503(4)	2.482(3)	2.443(6)	2.439(4)	2.408(4)
**P(1)–Ln(1)–P(2)/**°	129.46(3)	128.99(4)	129.30(4)	128.90(3)	128.80(5)
**P(1)–Ln(1)–P(3)/**°	117.85(3)	118.23(4)	118.30(4)	118.66(4)	119.44(6)
**P(2)–Ln(1)–P(3)/**°	112.39(3)	112.58(4)	112.06(4)	112.22(4)	111.52(6)
**O(1)–Ln(1)–O(2)/**°	175.14(8)	175.6(6)	175.58(13)	175.68(9)	175.36(13)
Ln···P_3_	0.0901(7)	0.0732(8)	0.0957(11)	0.0761(8)	0.0781(10)
**Σ angles about P/**°	320.45(8)	331.58(12)	323.81(14)	331.9(3)	330.4(5)
	353.38(8)	351.06(11)	353.47(13)	351.12(9)	351.34(14)
	358.66(8)	359.28(11)	358.76(13)	359.03(10)	358.95(14)

All **1-Ln** exhibit distorted trigonal bipyramidal geometries, with three equatorial phosphide ligands adopting a “propeller” arrangement and two axial THF molecules, in common with the structures of **1-Tm**(37) and **1-Nd**;^38^ individual P–Ln–P and O–Ln–P angles vary by up to 11° from 120° and 90°, respectively. The O–Ln–O angles lie in a narrow range from 175.14(8) to 175.68(9)°, and the Ln centers are all located <0.1 Å from the P_3_ planes; there are no obvious trends in these parameters between larger and smaller Ln;^37,38^ thus, these discrepancies can be attributed to crystal packing effects.^46^ The large deviation of the central Ln-P_3_O_2_ cores from ideal polyhedra is shown by their τ_5_ values (**1-La**: 0.76, **1-Ce**: 0.78, **1-Pr**: 0.77, **1-Nd**: 0.78, **1-Sm**: 0.78), where τ_5_ = 1 is a trigonal bipyramid and τ_5_ = 0 is a square-based pyramid (τ_**5**_*=* (β ***–*** α)/60, where β is the largest and α the second-largest angle in the coordination sphere).^47^ As the Ln series is traversed for **1-Ln**, the mean Ln–P and Ln–O bond lengths decrease, consistent with the decrease in Ln(III) ionic radii with increasing atomic number;^48^ the range of mean Ln–P (2.886(2) Å for **1-La** vs. 2.278(9) Å for **1-Sm**) and Ln–O (2.503(4) Å for **1-La** vs. 2.408(4) Å for **1-Sm**) bond lengths for **1-Ln** reported herein is longer than the respective distances previously reported for **1-Tm** (Tm–P: 2.705(3) Å; Tm–O: 2.315(3) Å).^37^

Complexes **1-Ln** are analogous to [Ln{Si(SiMe_3_)_3_}_3_(THF)_2_] (Ln = La, Ce, Pr, Nd), which show τ_5_ values between 0.98 and 1.00 because each silanide ligand bears three trimethylsilyl groups, leading to relatively long Ln–Si bonds (3.131(2)–3.197(3) Å) and smaller distortions from ideal local D_3*h*_ geometries in the ss due to lower metal coordination sphere saturation.^25^ By contrast, the related Ln(III) silylamide complexes [Ln{N(SiMe_3_)_2_}_3_] are solvent-free and trigonal pyramidal in the ss due to their relatively short Ln–N bonds and strong agostic-type electrostatic interactions between Ln(III) ions and β-Si–C bonds providing electronic and steric saturation.^13,34^ The structures of **1-Ln** are similar to the Ln(III) silylamide complexes [Ln{N(SiMe_2_H)_2_}_3_(THF)_2_],^49−51^ which show more bent O–Ln–O angles (*ca*. 162°) and mean Ln–N distances that are ca. 0.4 Å shorter than the Ln–P bonds in **1-Ln** for the corresponding metal; the mean Ln–O distances of [Ln{N{SiMe_2_H)_2_}_3_(THF)_2_] are ca. 0.1 Å longer than their **1-Ln** analogues.^49−51^ The smaller ligand substituents and donor atoms in [Ln{N(SiMe_2_H)_2_}_3_(THF)_2_] lead to mean Ln–N distances that are approximately 0.4 Å shorter than the corresponding Ln–P bonds in **1-Ln** for the same metal, which is not entirely accounted for by the sum of the Pyykkö covalent radii (Ln–N = 0.278 Å; Ln–P = 0.314 Å).^52^ The resultant saturation of the metal coordination spheres in [Ln{N(SiMe_2_H)_2_}_3_(THF)_2_] leads to their mean Ln–O distances being ca. 0.1 Å longer than those of the corresponding **1-Ln** analogue.

The pyramidalization of P atoms in **1-Ln**, defined here as the deviation from 360° of the sum of Si–P–Si and Ln–P–Si angles, is different for all three phosphide ligands in **1-Ln**; one P center is close to planarity, one deviates only slightly, and the other shows a more pronounced difference. Both **1-La** and **1-Pr** show the widest gamut of pyramidalization values (320.45(8)–358.66(8)° and 323.81(14)–358.76(13)°, respectively), while **1-Ce** (331.58(12)–359.28(11)°) **1-Nd** (331.9(3)–359.03(10)°), and **1-Sm** (330.4(5)–358.95(14)°) show narrower ranges. This observation is attributed to saturation of the metal coordination spheres and appears to manifest in differences in the spread of the CSA in ss ^31^P NMR spectra (see below). This feature is not seen for [Ln{N(SiMe_2_H)_2_}_3_(THF)_2_], where all N atoms are planar; this is likely due to their sp^2^-hybridized lone pairs interacting with σ* orbitals associated with the Si–C bonds of the trimethylsilyl groups by negative hyperconjugation.^49−51^ The pyramidalization of P centers in terminal Ln phosphides has frequently been observed and commented upon, e.g., for [La(P^t^Bu_2_)_2_(μ-P^t^Bu_2_)_2_Li(THF)],^53^ [Ln(PPh_2_)_2_(THF)_4_] (Ln = Sm, Yb),^54^ [Sm(PPh_2_)_2_(*N*-MeIm)_4_] (*N*-MeIm = *N*-methylimidazole),^55^ and [Ln{P(H)(Mes*)}_2_(THF)_4_] (Ln = Eu, Yb; Mes* = C_6_H_2_^t^Bu_3_-2,4,6).^55^ Conversely, near-planar P atoms were observed for [Ln{P(Mes)_2_}_2_(THF)_4_] (Ln = Sm, Yb; Mes = C_6_H_3_Me_3_-2,4,6); this was ascribed to the sterically demanding phosphide substituents.^56^

### Solution NMR Spectroscopy

The ^1^H, ^13^C{^1^H}, ^29^Si DEPT90, and ^31^P{^1^H} NMR spectra collected for solutions of **1-Ln** in C_6_D_6_ were all fully assigned (see Table 2 for chemical shifts, coupling constants and selected fwhm for broad signals, and Supporting Information Figures S21–S40 for all spectra). For diamagnetic **1-La**, three resonances were observed in the ^1^H and ^13^C{^1^H} NMR spectra at expected chemical shifts for the magnetically equivalent SiMe_3_ groups and the two THF ^1^H and ^13^C environments. These results contrast with the related M(III) silanide complexes [M{Si(SiMe_3_)_3_}_3_(THF)_2_] (M = La, Ce, Pr, Nd, U), where the bound THF molecules were found to dissociate in aromatic solvents, leading to rapid decomposition at room temperature in the absence of excess THF.^25^ There is no evidence of THF dissociation in neat C_6_D_6_ solutions of **1-Ln**, which are shown to be stable for several days at room temperature by multinuclear NMR spectroscopy; thus, we posit that the ss structures are essentially maintained in the solution but with dynamic averaging of equivalent nuclear environments. The ^29^Si DEPT90 NMR spectrum of **1-La** contains a doublet at 2.66 ppm (^1^*J*_SiP_ = 22.4 Hz), as expected from the coupling of ^29^Si nuclei to 100% abundant *I* = 1/2 ^31^P nuclei; the coupling constant is typical for a P–Si bond, e.g., ^1^*J*_SiP_ = 18.1 Hz for the pentacoordinate silirane [Si(CH_2_CH_2_)(Ph){norbornene-(NDipp)-1-(PPh_2_)-2}] (Dipp = C_6_H_3_^i^Pr_2_-2,6).^57^ A broad and asymmetric signal was observed in the ^31^P{^1^H} NMR spectrum of **1-La** at −113.0 ppm (fwhm ≈1150 Hz), where coupling constants could not be readily extracted due to quadrupolar broadening caused by ^139^La nuclei (abundance = 99.95%; *I* = ^7^/_2_). This observation is typical for La(III) phosphide complexes, e.g., [La(P^t^Bu_2_)_2_(μ-P^t^Bu_2_)_2_Li(THF)] in C_6_D_6_ (δ{^31^P} = 158 ppm, fwhm = 3,000 Hz).^53^

**Table 2:** ^1^H, ^13^C{^1^H}, and ^29^Si DEPT90 and ^31^P{^1^H} NMR Chemical Shifts (δ), Coupling Constants (Hz), and Selected FWHM Values for **1-Ln** in C_6_D_6_

**complex**	^1^H (δ)	^13^C{^1^H} (δ)	^29^Si DEPT90 (δ)	^31^P{^1^H} (δ)
**1-La**	0.54 (s, C*H*_3_)	7.49 (*C*H_3_)	2.66 (d, *Si*Me_3_)	–113.0 (br, *P*-La)
	1.46 (m, THF-β-H)	25.37 (THF-β-C)	^1^*J*_SiP_ = 22.4 Hz	fwhm ≈1150 Hz
	4.37 (m, THF-α-H)	73.20 (THF-α-C)		
**1-Ce**	–2.17 (s, C*H*_3_)	7.93 (*C*H_3_)	5.30 (*Si*Me_3_)	616.7 (br, *P*-Ce)
	1.02 (br, THF-β-H)			fwhm ≈350 Hz
	fwhm ≈ 70 Hz			
	8.33 (br, THF-α-H)			
	fwhm ≈ 180 Hz			
**1-Pr**	–6.83 (s, C*H*_3_)	11.17 (*C*H_3_)	15.65 (*Si*Me_3_)	1894.2 (br, *P*-Pr)
	23.50 (br, THF-β-H)			fwhm ≈550 Hz
	fwhm ≈ 450 Hz			
	48.12 (br, THF-α-H)			
	fwhm ≈ 960 Hz			
**1-Nd**	–2.24 (s, C*H*_3_)	23.94 (*C*H_3_)	42.94 (*Si*Me_3_)	2570.3 (br, *P*-Nd)
	11.75 (br, THF-β-H)			fwhm ≈1100 Hz
	fwhm ≈ 130 Hz			
	23.52 (br, THF-α-H)			
	fwhm ≈ 360 Hz			
**1-Sm**	0.09 (s, C*H*_3_)	5.47 (*C*H_3_)	0.52 (*Si*Me_3_)	–259.2 (br, *P*-Sm)
	2.35 (br, THF-β-H)	26.15 (THF-β-C)		fwhm ≈1500 Hz
	fwhm ≈ 10 Hz	78.89 (THF-α-C)		
	6.11 (br, THF-α-H)			
	fwhm ≈ 20 Hz			

In the previous report of **1-Nd**, only one signal was observed in the ^1^H NMR spectrum of a C_6_D_6_ solution at −2.24 ppm and this was assigned to the SiMe_3_ groups; THF resonances were not observed in the ^1^H NMR spectrum and no signals were seen in the ^13^C{^1^H}, ^29^Si{^1^H}, and ^31^P{^1^H} NMR spectra collected due to paramagnetic broadening of resonances into the baseline.^38^ However, cryogenic NMR probes can provide drastically increased sensitivity for NMR experiments by reducing thermal noise,^58^ and as we have this facility, we were able to observe resonances in the ^1^H, ^13^C{^1^H}, ^29^Si DEPT90, and ^31^P{^1^H} NMR spectra for all paramagnetic **1-Ln** reported herein following experimental optimizations (see Supporting Information Tables S5 and S6 for parameters used). The expected three resonances were seen in the ^1^H NMR spectra for all **1-Ln**, with paramagnetic shifts and the extent of broadening varying with the magnetic anisotropy of the Ln(III) ion.^1^ However, the expected three signals in the ^13^C{^1^H} NMR spectra were only seen for **1-Sm** (and diamagnetic **1-La**), where the Sm(III) ion shows diminished paramagnetic behavior as expected due to its low magnetic moment.^1^ Only SiMe_3_ signals were observed in the ^13^C{^1^H} NMR spectra of other paramagnetic **1-Ln**, in accord with these groups experiencing a smaller paramagnetic shift than the THF ^13^C NMR nuclei and assuming that the effects are similar to those observed for the corresponding ^1^H resonances. The ^29^Si DEPT90 NMR spectra of paramagnetic **1-Ln** show similar trends in the paramagnetic shifts, but we were unable to extract ^1^*J*_PSi_ coupling constants.

**Figure 2:**
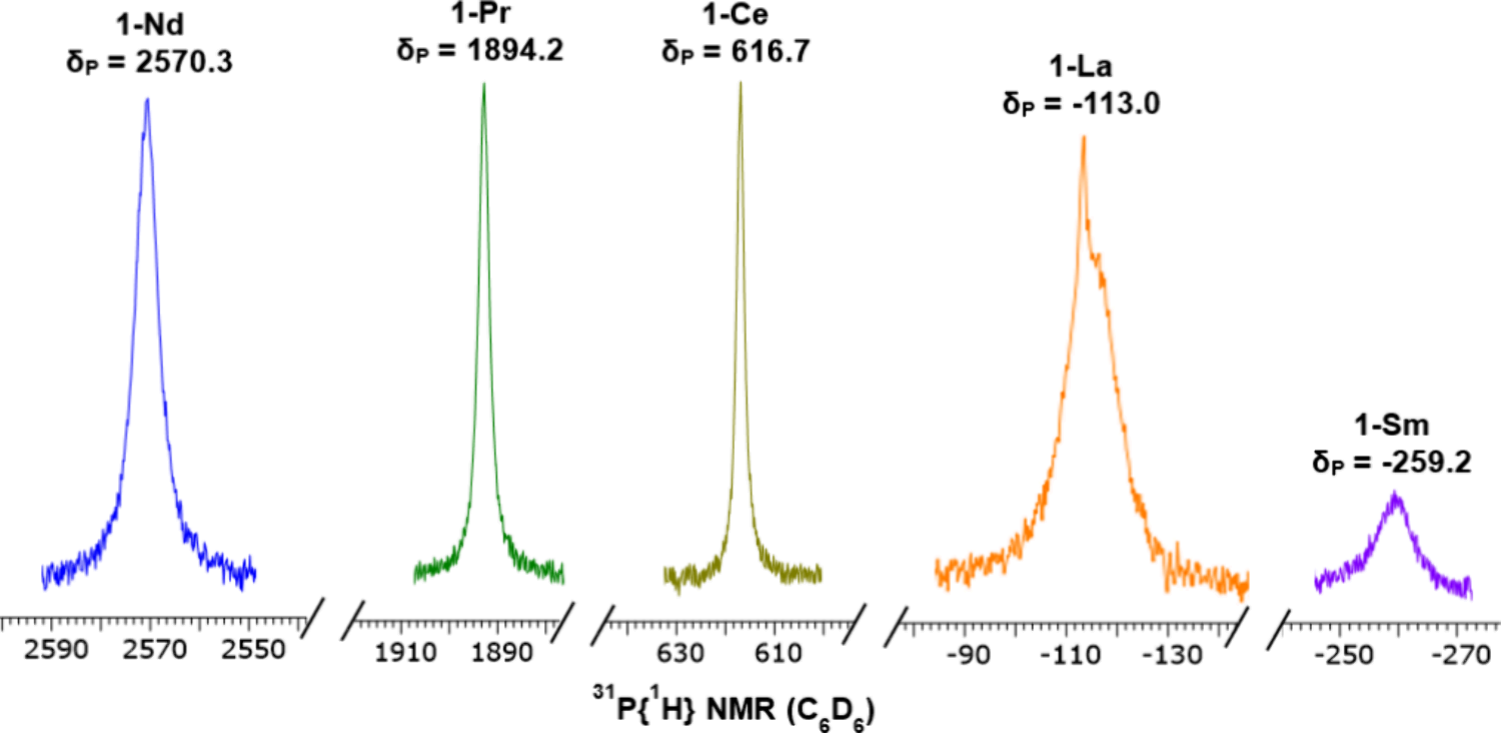
Signals observed in the ^31^P{^1^H} NMR spectra of **1-Ln**.

Metal-bound phosphide resonances were observed by ^31^P{^1^H} NMR spectroscopy for all paramagnetic **1-Ln** (Figure 2), together with trace amounts of either KP(SiMe_3_)_2_ (δ{^31^P} = −296 ppm),^44^ (Me_3_Si)_2_P–P(SiMe_3_)_2_ (δ{^31^P} = −216 ppm),^59^ HP(SiMe_3_)_2_ (δ{^31^P} = −237.4 ppm),^60^ and/or H_2_PSiMe_3_ (δ{^31^P} = −239.0 ppm).^60^ In contrast to the sharp metal-bound silanide resonances seen in the ^29^Si DEPT90 NMR spectra of [Ln{Si(SiMe_3_)_3_}_3_(THF)_2_],^25^ the signals in the ^31^P{^1^H} NMR spectra of **1-Ln** for Ln = Ce, Pr, Nd, and Sm were all subject to significant broadening by the respective Ln(III) ion, and the large paramagnetic shifts required the acquisition of multiple spectra to cover a wide range of chemical shifts. To the best of our knowledge, δ{^31^P} values have not been reported for any other paramagnetic molecular Ln silylphosphide complex to date; however, there has been a reported chemical shift of 2055.21 ppm for the U(IV) complex [U(Tren^DMBS^){P(SiMe_3_)_2_}].^52^ We attribute the increased intensities of signals for **1-Ln** here to a combination of the use of a cryoprobe and the presence of three symmetry-related ^31^P nuclei in the solution.^33^

For **1-Ce** (δ_P_ = 616.7 ppm), **1-Pr** (δ_P_ = 1894.2 ppm), and **1-Nd** (δ_P_ = 2570.3 ppm), the resonances show a downfield trend in the isotropic chemical shift with increasing paramagnetism of the Ln(III) ion from Ce–Nd; a search of the literature indicated that **1-Nd** exhibits the most deshielded δ_P_ value that has been observed for any Ln complex to date.^33^ By contrast, **1-Sm** shows a broad resonance at δ_P_ = −259.2 ppm that is an upfield of diamagnetic **1-La**, as the less paramagnetic Sm(III) ion does not induce as large a shift, and the ordering of *m*_J_ states is expected to be the opposite of Ce(III), Pr(III), and Nd(III) in the same ligand environment.^61^

### ssNMR Spectroscopy

**Figure 3:**
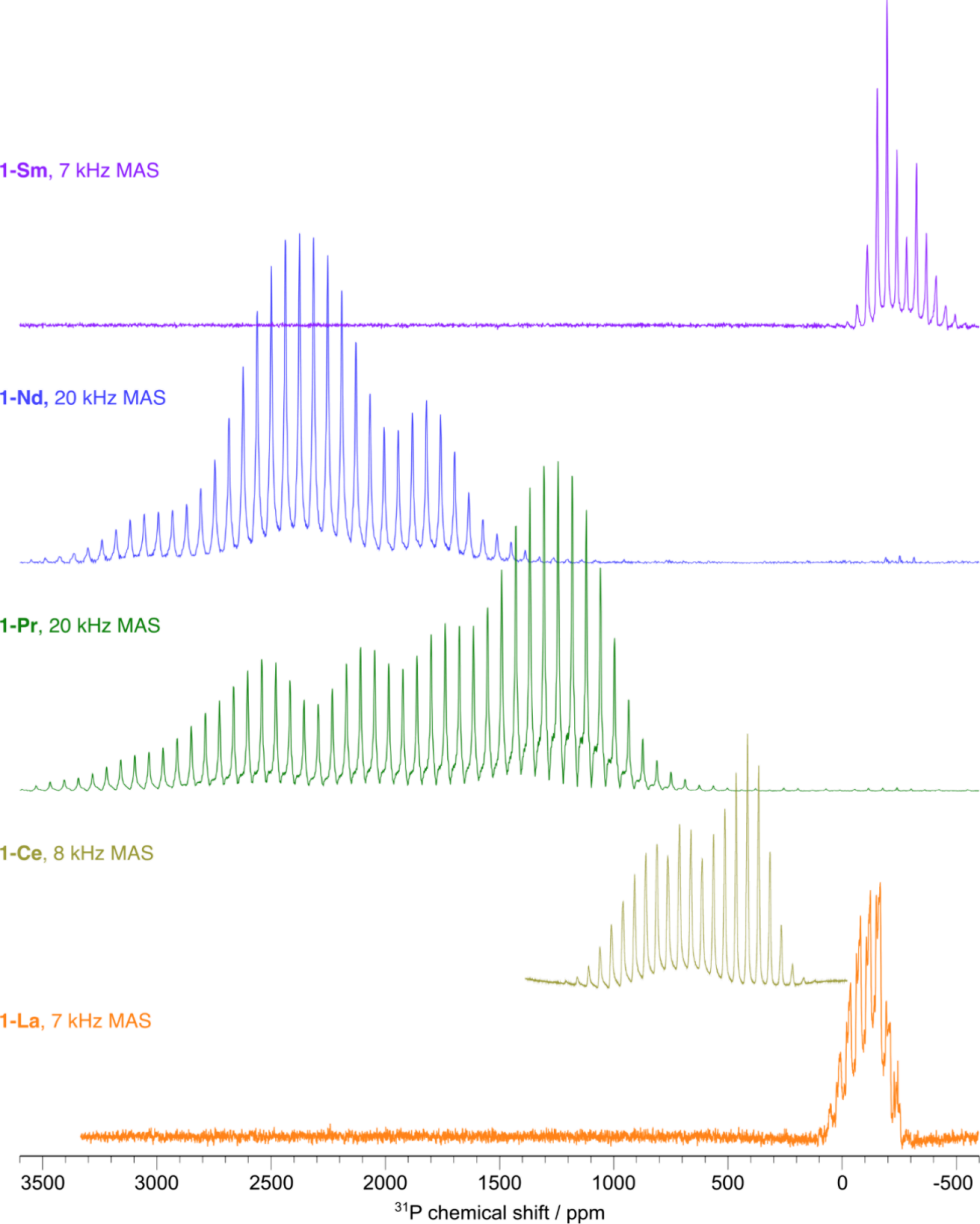
^31^P MAS NMR spectra for **1-La**, **1-Ce**, and **1-Sm** and ^31^P WCPMG-MAS NMR spectra^62^ for **1-Pr** and **1-Nd** recorded at ambient temperature using the indicated MAS frequencies. Fitting of the spinning sideband manifolds can be found in Supporting Information Figures S41–S45.

To get a fuller appreciation of the effect of the magnetic anisotropy of **1-Ln**, ^31^P and ^1^H MAS NMR spectra were recorded of the pure solids (Figure 3 and Supporting Information Figures S41–S46). Unlike for the analogous ^29^Si MAS NMR of [Ln{Si(SiMe_3_)_3_}_3_(THF)_2_],^25^ the ^31^P resonances from the metal-bound nuclear spins can be observed for all **1-Ln**; this is owing to the much greater NMR receptivity of ^31^P compared to ^29^Si (∼180 times greater). For diamagnetic **1-La**, it is evident that on top of the spinning sideband pattern that can be used to determine the CSA parameters (Table 3), there are some underlying features (see Figure S41 for the calculated fit). The *J*-coupling of the ^31^P spins to ^139^La (abundance = 99.95%; *I* = ^7^/_2_) results in a splitting of the resonance into an eight-line multiplet, each line of which has a different broadening dependent on the unequal lifetimes of the ^139^La Zeeman states and the ^1^*J*_LaP_ value (∼500 Hz). Here, the broadening is such that this multiplet cannot be resolved but its effect on the line shape can be observed. There are also small, relatively narrow resonances detected at δ{^31^P} = −251 and −235 ppm, which correspond to P(SiMe_3_)_3_ (δ{^31^P} = −251.2 ppm) and HP(SiMe_3_)_2_ and/or H_2_PSiMe_3_^60^ degradation product(s), respectively. There is only a small difference in the isotropic chemical shift between the solid (δ_iso-ss_{^31^P} = −123 ppm) and solution NMR spectra (δ_iso-sol_{^31^P} = −113 ppm), indicating that the solution and solid structures of **1-La** are similar. The span, Ω, describes the breadth of the observed powder pattern to provide a measure of the “magnitude” of the CSA (and associated tensor), while the skew, κ, describes the asymmetry of the CSA (and tensor); an axially symmetric tensor has κ = ± 1. For **1-La**, the ^31^P chemical shift tensor is not axially symmetric (κ ≠ ± 1). The span of almost 300 ppm, as well as the downfield isotropic ^31^P chemical shift compared to HP(SiMe_3_)_2_,^60^ highlights a large deshielding of the ^31^P nucleus due to bonding to La.

**Table 3:** ^31^P MAS NMR Parameters for **1-Ln**. δ_iso_ = Isotropic Shift; δ_11_, δ_22_, δ_33_ = Principal Components of Chemical Shift Tensor; Ω = Span = δ_11_ – δ_33_; κ = Skew = 3(δ_22_ – δ_*iso*_)/Ω^63^^,^^a^

**complex**	MAS/kHz	^31^P δ_iso_/ ppm	^31^P δ_11_/ ppm	^31^P δ_22_/ ppm	^31^P δ_33_/ ppm	^31^P Ω/ ppm	^31^P κ
**1-La**	7	–123	32	–139	–262	295	–0.16
**1-Ce**P(1,2)	8	561	1017	415	252	765	–0.57
**1-Ce P(3)**	8	763	1108	753	428	680	–0.04
**1-Pr P(1,2)**	20	1676	2960	1187	882	2078	–0.71
**1-Pr P(3)**	20	2046	3547	1366	1224	2322	–0.88
**1-Nd P(1,2)**	20	2191	2767	2299	1506	1261	0.26
**1-Nd P(3)**	20	2685	3341	2585	2129	1212	–0.25
**1-Sm**	7	–245	–96	–180	–444	348	0.52

a These are not definitive due to large relative errors in the fitting.

As with diamagnetic **1-La**, the isotropic ^31^P chemical shifts in the MAS NMR spectra of paramagnetic **1-Ln** are similar to those seen in the solution ^31^P{^1^H} NMR spectra (Table 3). For paramagnetic **1-Ln**, the analysis becomes more complex owing to the (hyperfine) interaction between the unpaired electron spins of Ce(III), Pr(III), Nd(III), and Sm(III) and the nuclear spins. Since there is a direct bond between the paramagnetic Ln and ^31^P nuclei, CS, PCS, and PSO contributions to the paramagnetic shift are all likely to be significant; conversely, for the shifts of ^1^H nuclei in paramagnetic **1-Ln**, the PCS term will dominate. The ^1^H shifts observed for solid **1-Ln** are very similar to those in the solution (|δ_iso-ss_{^1^H} – δ_iso-sol_{^1^H}| < 0.1 ppm for **1-La** and **1-Sm** and <2.2 ppm for **1-Nd** and **1-Pr**), but the ^1^H MAS NMR spectra are substantially broadened by paramagnetism (see Figure S46). The ^31^P MAS NMR spectra of **1-Ce**, **1-Pr**, and **1-Nd** in Figure 3 have substantial spans (Ω ≈ 700, 2200, and 1200 ppm, respectively; see Table 3), whereas that of **1-Sm** is much less (Ω ≈ 350 ppm). The magnitude of the Ω values is generally consistent with the relative extents of paramagnetism of Ln ions; **1-Pr** has a larger span than expected, which we ascribe to the greater differences in geometries of P atoms of this complex in the ss compared to **1-Ce** and **1-Nd** (Table 1). The asymmetry (skew, κ) for **1-Nd** and **1-Sm** is opposite that of **1-Ce** and **1-Pr**, which could indicate a change in the anisotropy of the magnetic susceptibility of the Ln or a change in ambient structural motion owing to the shorter Ln–P bonds of **1-Nd** and **1-Sm** compared to those of **1-Ce** and **1-Pr**. We note here that the observed CSA may be affected by the bulk magnetic susceptibility of the ensemble, i.e., the demagnetizing field associated with the net magnetization of the sample, which we have not accounted for here. This effect is well-summarized by Pell, Pintacuda, and Grey in their seminal review.^26^ Usually for molecular crystals of Ln complexes, the demagnetizing fields are not large compared to densely packed inorganic materials and do not need to be considered; we find that our results show good resolution and are well-modeled by a conventional second-rank tensor, which suggests these effects are small in the present case. The ^31^P MAS NMR spectra of **1-Ce**, **1-Pr**, and **1-Nd** each have two clear components, one major and one minor (see Supporting Information Figures S42–S44). This can be attributed to the 2:1 ratio of planar/pyramidal ^31^P environments of ss **1-Ln** (Figure 1); only one environment is observed in the solution ^31^P{^1^H} NMR spectra of **1-Ln** due to dynamic structural motion. This is validated by the isotropic ^31^P chemical shifts, where the weighted average is similar to that seen in the solution (δ_iso-ss,avg_{^31^P} = 628 ppm and δ_iso-sol_{^31^P} = 617 ppm for **1-Ce**; δ_iso-ss,avg_{^31^P} = 1799 ppm and δ_iso-sol_{^31^P} = 1894 ppm for **1-Pr**; δ_iso-ss,avg_{^31^P} = 2356 ppm and δ_iso-sol_{^31^P} = 2570 ppm for **1-Nd**). It is somewhat surprising that two components are not observed for **1-Sm** or **1-La**. This could be due to the reduced or no paramagnetic effects, which would otherwise help to distinguish these components. There are also unidentified components in the ^31^P MAS NMR spectrum of **1-La** (Figure S41) that could stem from a second component, but overlapping signals (due to *J*-splitting and CSA) prevent resolution.

### UV–Vis-NIR Spectroscopy

**Figure 4:**
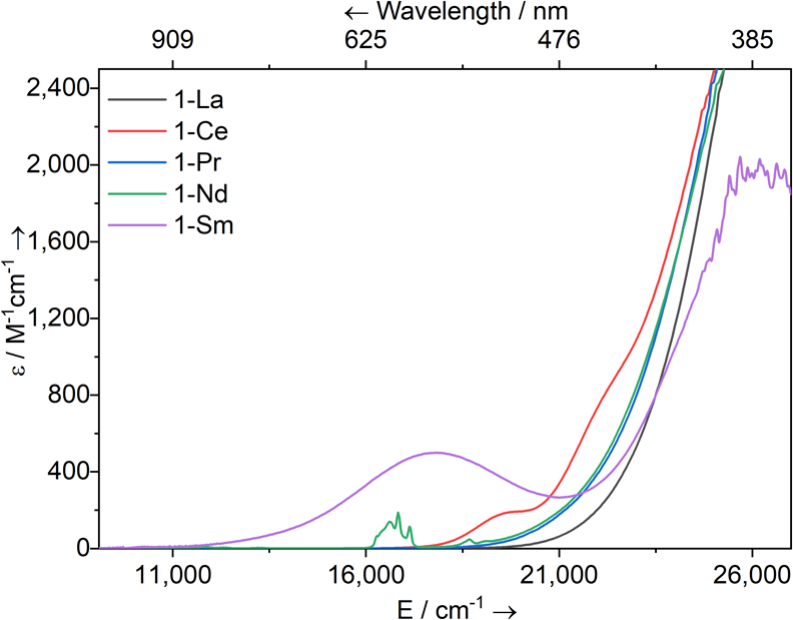
UV–vis-NIR spectra of **1-Ln** (2 mM in toluene) between 9000 and 27,000 cm^–1^ (1111–370 nm). Legend: M = La (block), Ce (red), Pr (blue), Nd (green), and Sm (purple).

Complexes **1-Ln** vary in color, with solutions ranging from pale yellow (**1-La**) to deep purple (**1-Sm**). To study these electronic transitions further, the UV–vis-NIR spectra of 2 mM toluene solutions of **1-Ln** were recorded at room temperature (compiled in Figure 4; see Supporting Information Figures S49–S53 for individual spectra). All spectra exhibited an intense charge transfer (CT) absorption tailing into the visible spectrum from the UV region. For **1-Ce**, two shoulders were seen at υ̃_max_ = 22,000 cm^–1^ (ε = 750 M^–1^ cm^–1^) and 19,500 cm^–1^ (ε = 180 M^–1^ cm^–1^); the lower energy absorption may be due to a 4f^1^ → 5d^1^ transition, as assigned previously for [Ce{Si(SiMe_3_)_3_}_3_(THF)_2_] (υ̃_max_ = 23,300 cm^–1^, ε = 240 M^–1^ cm^–1^).^25^ The electronic spectrum of **1-Nd** has a weak absorption at υ̃_max_ = 18,650 cm^–1^ (ε = 50 M^–1^ cm^–1^) as well as a set of absorptions in the visible region (υ̃_max_ = 16,100–17,300 cm^–1^; ε = 190 M^–1^ cm^–1^) that are assigned as f-f transitions arising from the ^4^I_9/2_ → ^4^G*_J_* states;^64^ a similar feature was previously observed for [Nd{Si(SiMe_3_)_3_}_3_(THF)_2_].^35^ For **1-Sm**, there is a broad and relatively intense absorption in the visible region (υ̃_max_ = 17,800 cm^–1^; ε = 500 M^–1^ cm^–1^). This is unusual as Sm(III) complexes typically only show weak f-f transitions in this region.^64^ However, we have noted previously that the CT band tends to tail in further into the visible region for Sm(III) homologues of a structurally analogous series of light Ln(III) complexes, and that more axial ligand fields can effect a greater blue-shift for the CT bands of similar Sm(III) complexes.^65^ We therefore assign this feature to a ligand metal CT (LMCT) that arises due to significant orbital mixing of the 4f and 5d orbitals and vibronic coupling occurring in **1-Sm**.^1^ A similar strong absorbance has been observed in the spectrum of [Eu(N″)_3_] at approximately 16,000 cm^–1^, which was ascribed to an LMCT.^34^

### Magnetism

**Figure 5:**
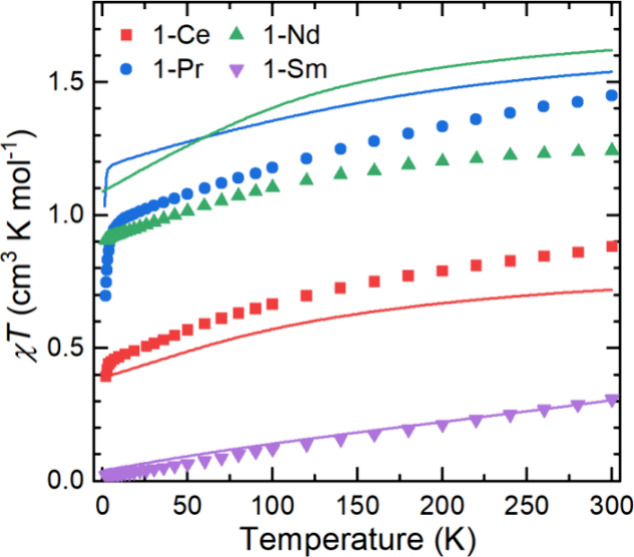
Temperature dependence of χ*T* for **1-Ln** (symbols) along with CASSCF-calculated curves (solid lines).

The molar magnetic susceptibility (χ_M_*T*) of powders of paramagnetic **1-Ln** suspended in eicosane was examined by variable-temperature DC SQUID magnetometry (compiled in Figure 5 with selected parameters in Table 4; see Supporting Information Figures S54–S61 for all magnetic data) and CASSCF-SO calculations (see below). There is excellent agreement between measured and calculated susceptibility values for **1-Sm**; at 300 K, these are slightly higher than the free ion value (Sm(III) 4f^5 6^H_5/2_) due to the mixing of low-lying ^6^H*_J_* excited terms with the ground term. The magnetization data for **1-Sm** are slightly lower than the CASSCF-SO-calculated values, but given their small magnitude, they are more susceptible to experimental uncertainties. The absolute values of χ_M_*T* and magnetization are alternately under- (**1-Ce**, 4f^1 2^F_5/2_) and overpredicted (**1-Pr**, 4f^2 3^H_4_; **1-Nd**, 4f^3 4^I_9/2_) by CASSCF-SO calculations; at 300 K, the largest discrepancy is seen for **1-Nd** (ca. 0.4 cm^3^ K mol^–1^), which should be investigated in the future. However, the shapes of the variable-temperature χ_M_*T* and variable field *M* traces of all **1-Ln** are accurately reproduced by CASSCF-SO-predicted values, showing a gradual decrease in χ_M_*T* with *T* due to thermal depopulation of excited crystal field states. For **1-Pr**, the sharp drop in χ_M_*T* < 5 K can be attributed to Pr(III) being a non-Kramers ion and subsequent polarization of the ground state at low temperatures.

**Table 4:** Product of the Molar Susceptibility and Temperature, χ_M_*T* (cm^3^ mol^–1^ K), of **1-Ce**, **1-Pr**, **1-Nd**, and **1-Sm** Determined by SQUID Magnetometry at 1.8 and 300 K, Calculated CASSCF-SO Values at 2 and 300 K, and Free Ion Calculated Values at 300 K

	SQUID magnetometry		CASSCF-SO calculations		free ion^1^
complex	1.8 K	300 K	2 K	300 K	χ_M_*T*
**1-Ce**	0.39	0.88	0.39	0.72	0.81
**1-Pr**	0.70	1.45	1.15	1.54	1.60
**1-Nd**	0.91	1.24	1.10	1.62	1.64
**1-Sm**	0.02	0.31	0.04	0.30	0.09

### EPR Spectroscopy

**Figure 6:**
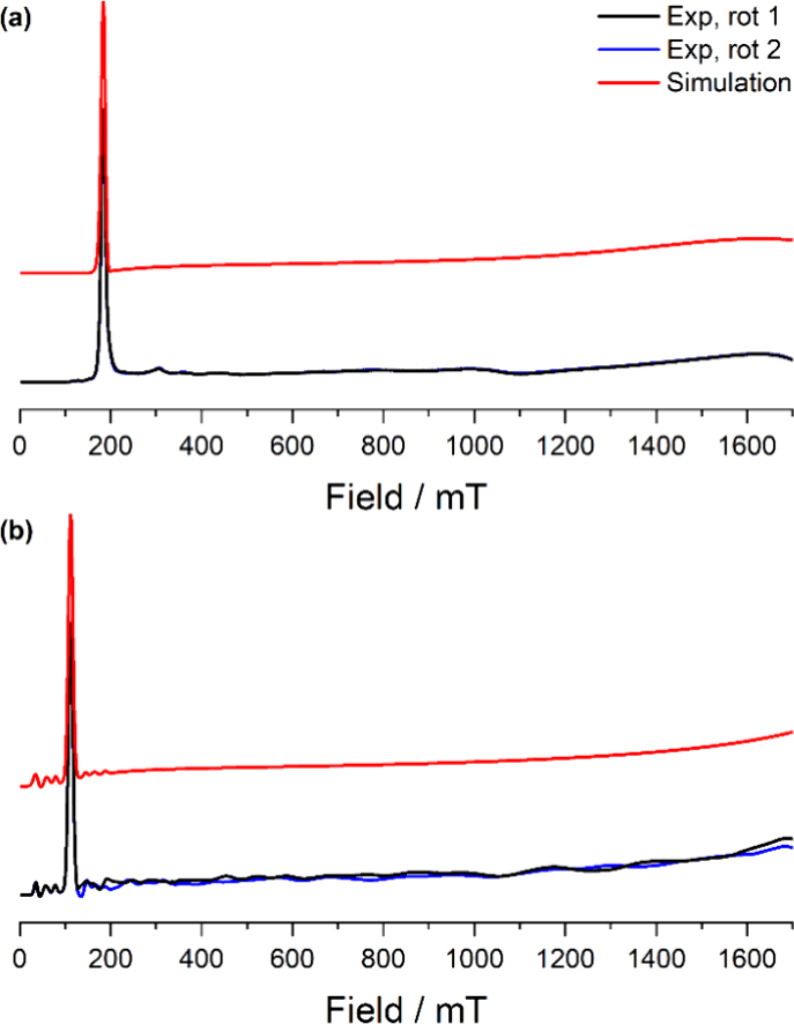
CW X-band EPR spectra of (a) **1-Ce** powder at 7 K and (b) **1-Nd** powder at 12–16 K. Two perpendicular orientations of powder spectra are shown in black and blue. Simulations using parameters from Supporting Information Table S9 are shown in red.

The electronic structures of Kramers ion complexes **1-Ce** and **1-Nd** were further investigated by c.w. X-band (ca. 9.4 GHz) EPR spectroscopy on ground polycrystalline samples, using EasySpin^66^ to model the data (Figure 6; see Supporting Information Table S9 for full details of simulations). Attempts were made to collect spectra of **1-Sm** but the results were not reproducible, consistent with decomposition occurring upon sample grinding. The spectra are highly axial, with *g*_*z*_ values of 3.598 (**1-Ce**) and 5.969 (**1-Nd**), indicating that ground states are dominated by the highest *m*_*J*_ component of the ground *J* multiplet; these are very close to those predicted by CASSCF-SO calculations (3.498 and 5.874, respectively, Tables S17 and S19).

### DFT Calculations

The electronic structure of **1-La** was calculated with a variety of DFT methods using ORCA v.5.0 (see Experimental Section for details).^67^ The models of **1-La** used included the XRD-determined atomic positions with and without geometry-optimized H-positions and fully geometry-optimized structures starting from the XRD structure (see Supporting Information Tables S10–S14 for geometry-optimized atomic positions). ^31^P NMR chemical shielding tensors were calculated in all cases. A variety of methods were used, including BP86 and B3LYPHFXX with exact exchange contributions ranging from 20 to 50% (see Supporting Information Table S15 for the full results). While PBE0^68−75^ and SAOP^76−78^ functionals have been most extensively used to date to calculate NMR chemical shifts of f-block metal complexes, calculations with B3LYPHF20–50 hybrid-functionals have also provided good correlation of ^13^C, ^15^N, ^29^Si, and ^31^P NMR parameters when these atoms are bonded to f-block ions;^25,79−81^ B3LYPHF50 was previously found to be optimal for predicting chemical shifts of thorium-bound phosphorus atoms.^80^

In this case, optimization of H-positions gave the best agreement with the experiment (Table 5). B3LYPHF20, corresponding to Becke’s popular three-parameter fit of the hybrid exchange-correlation functional,^82^ achieved close agreement with the experimental isotropic shifts, but the span of the anisotropy was larger than that observed experimentally (in all calculations, see Table S15) and the predicted skew was more negative. However, these were of the correct order of magnitude, and the skew had the correct sign. The optimal Hartree–Fock exchange contribution to best match the experimental solid-state isotropic shift was 30%. The agreement achieved with this functional is most likely due to cancellation of errors, however, especially from the neglect of dynamics in our calculations; B3LYPHF30 should not be expected to give an improved description of the electronic structure of the complex. This interpretation is supported by the very different optimal exact exchange contribution found by the previous fit to f-element bound ^31^P NMR data.^80^

**Table 5:** Experimental and Calculated ^31^P Chemical Shifts (δ_iso_*vs*. 85% H_3_PO_4_) and Anisotropy Parameters for **1-La**, Using Both XRD and Optimized Structures with Different DFT Methods^a^

method	structure	^31^P (exp) δ_iso_ (ppm)^d^	^31^P δ_11_ (ppm)^d^	^31^P δ_22_ (ppm)^d^	^31^P δ_33_ (ppm)^d^	Span, Ω (ppm)^d^	Skew, κ^d^
experimental		–123,^b^ – 113^c^	32	–139	–262	295	–0.16
B3LYPHF20	XRD	–85.6	270.5	–200.4	–326.8	597.3	–0.58
	H-Opt	–109.0	224.3	–203.3	–348.1	572.3	–0.49
	Opt	–66.5	283.3	–100.9	–382.1	665.4	–0.15
B3LYPHF30	XRD	–97.5	243.7	–206.1	–330.2	573.9	–0.57
	H-Opt	–121.0	200.40	–213.9	–349.5	549.8	–0.51
	Opt	–56.5	227.2	–75.2	–321.6	548.8	–0.10

a δ_11_, δ_22_, δ_33_ = principal components of chemical shift tensor; Span, Ω = δ_11_–δ_33_; Skew, κ = [3(δ_22_–δ_iso_)]/δ_11_–δ_33_)

b ^31^P MAS NMR data.

c Solution ^31^P{^1^H} NMR data.

d Only one value obtained upon averaging.

The DFT-calculated ^31^P NMR parameters are those of a static **1-La** structure. However, at ambient temperature, where the experimental NMR measurements were recorded, there will be dynamic averaging owing to local motions, if these are present. For instance, librational motion of the La–P bond will reduce the effective ^31^P shielding span. Simulating a fast (on the NMR time-scale) librational motion of the calculated ^31^P shielding tensor with an amplitude of ±32° gives an averaged spectrum (see Figure S47) that corresponds to the spectrum measured experimentally. Note that this librational motion is one of many possible solutions but highlights that **1-La** is not static. The relative directions and magnitudes of the principal components and associated shielding surfaces of the DFT-calculated ^31^P shielding tensor of the three P of **1-La** are given in Figure S48 and Table S8.

Natural bond orbital (NBO) analysis was carried out using the NBO 6.0 software in Orca, with the aim of characterizing the Ln–P bonds. The results presented in Table 6 use B3LYPHF20 with optimized H-positions (see Supporting Information Table S15 for results from other methods). These data indicate relatively weak covalent bonding that is slightly stronger for phosphorus atoms with a lower degree of pyramidalization. The bonding interaction can be split into σ and π components, and as expected, the σ NBO has significant contributions from phosphorus 3s and 3p orbitals and lanthanum 4f, 5d, and 6s orbitals, with the 5d contribution being most significant from lanthanum. The π NBO is similar but without significant contributions from the s orbitals. The NBOs are significantly polarized toward phosphorus. This polarization occurs to a greater degree than in related hypersilanide complexes,^25^ consistent with phosphorus being more electronegative than silicon.

**Table 6:** Mayer Bond Order and NBO Analysis of the P–La Bonds in **1-La**^a^

atom	Mayer bond order	σ NBO				π NBO			
		%La	%s/p/d/f	%P	%s/p	%La	%s/p/d/f	%P	%s/p
**P(1)**	0.8001	7.11	22.16/2.3/69.67/5.87	92.89	50.48/49.45	8.24	0.17/0.59/67.81/31.43	91.76	0.02/99.90
**P(2)**	0.7396	6.55	16.68/1.48/68.19/13.65	93.45	52.23/47.67	7.49	0.56/0.69/58.07/40.68	92.51	0.36/99.55
**P(3)**	0.7209	6.78	17.59/0.55/51.22/30.65	93.22	53.99/45.71	6.69	5.20/1.25/66.23/27.33	93.31	0.63/99.08

a Contributions from phosphorus d and f orbitals are not shown, as they are always insignificant.

### *Ab Initio* Calculations

Minimal active space CASSCF-SO calculations were performed on all paramagnetic **1-Ln** using OpenMolcas^83^ and the molecular structures from single-crystal XRD data, in order to probe their electronic structures (see Experimental Section for details; Supporting Information Figures S62–S65 and Tables S17–S20). The active spaces consist of seven 4f orbitals with the appropriate number of electrons in each case. A mixed ground state is observed for **1-Ce** (*g*_*z*_ = 3.498, 78% *m*_J_ = ± 5/2), as *g*_*z*_ is tilted away from the O–Ce–O *pseudo*-axis; the calculated *g*-value is in good agreement with that determined by EPR spectroscopy (see above). By contrast, the ground state for **1-Pr** is a more pure 97% *m*_J_ = ± 4 and ***∓*** 4 pseudo doublet, with a splitting of 0.9 cm^–1^ and the principal magnetic axis approximately coincident with O–Pr–O. Similarly, the *g*_*z*_ axis of **1-Nd** is aligned along O–Nd–O and the ground state is quite pure (*g*_*z*_ = 5.874, 89% *m*_J_ = ± 9/2), in good agreement with the experiment (see above). Finally, for **1-Sm**, the ground state is calculated to be 87% *m*_J_ = ± 5/2, with *g*_*z*_ almost perpendicular to O–Sm–O. The variation in principal *g*-axis orientation shows that there is significant competition between the two hard neutral O-donors of THF and three soft formally negatively charged P-donors of {P(SiMe_3_)_2_} in defining the anisotropy of the complexes;^84,85^ for the more magnetic **1-Pr** and **1-Nd**, the O atoms dominate, while this is less clear-cut for **1-Ce** and **1-Sm**.

### pNMR Calculations

The paramagnetic contributions to the experimental isotropic pNMR shifts of **1-Ce**, **1-Pr**, **1-Nd**, and **1-Sm** were approximated by subtracting the shifts of the diamagnetic complex **1-La** (see Table 7 and Experimental Section for details and Supporting Information Tables S21–S23). To calculate the paramagnetic shifts, a variety of methods have been employed. First, the common approach of assuming the nuclei are all in the long-range limit compared to the well-localized magnetic moment of the paramagnetic ion has been considered, and hence, we calculate PCS using the point-dipolar approximation for **ΔC**, ignoring all other contributions in eq 1.^86^ In the general case of low-symmetry structures, this takes the form of eq 2:
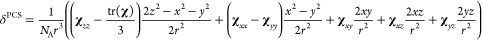
2where **χ** is the magnetic susceptibility tensor in c.g.s. e.m.u. (cm^3^ mol^–1^); *x*, *y*, and *z* are the Cartesian coordinates of the nucleus in question (in meters, where the Ln ion defines the origin), *r* is the distance between the nucleus and the Ln ion (in meters), and tr() indicates a matrix trace. We approximate **χ** by using CASSCF-SO calculations in OpenMolcas.^83^ The resulting calculated shifts are reasonably accurate for all ^1^H resonances, which are indeed significantly distal (3 or 4 bonds) to the Ln ions, suggesting that their contact shifts are effectively zero. Nonetheless, in some cases (**1-Ce** especially), the THF proton shifts have noticeably worse agreement with the experiment than the CH_3_ protons. This is expected from their proximity to the Ln ion. However, the trend is not universal due to other inaccuracies, such as those due to neglect of the dynamics. Calculated shifts for other nuclei are less accurate, although the calculated ^13^C shifts of **1-Sm** are in reasonable agreement with experiment.

**Table 7:** Experimental and Calculated Isotropic Paramagnetic NMR Shifts for Paramagnetic **1-Ln**

				calculated paramagnetic shift (ppm)		
nucleus	complex	environment	experimental paramagnetic shift (ppm)	magnetic susceptibility method	van den Heuvel method with CASSCF-SO	van den Heuvel method with RASCI-SO
^1^H	**1-Ce**	CH_3_	–2.17	–2.34	–2.39	–2.70
		THF-β	1.02	8.01	8.09	9.42
		THF-α	8.33	15.30	15.12	17.72
	**1-Pr**	CH_3_	–6.83	–10.16	–10.13	–11.95
		THF-β	23.50	32.71	32.59	37.41
		THF-α	48.12	61.38	60.75	69.77
	**1-Nd**	CH_3_	–2.24	–3.87	–3.92	–4.51
		THF-β	11.75	12.07	12.02	14.54
		THF-α	23.52	21.91	21.12	25.89
	**1-Sm**	CH_3_	0.09	–0.54	–0.53	–0.52
		THF-β	2.35	1.69	1.68	1.70
		THF-α	–6.11	2.53	2.52	2.54
^13^C	**1-Ce**	CH_3_	7.93	–2.90	–6.51	–2.39
		THF-β		12.05	10.41	–9.94
		THF-α		24.96	25.48	25.93
	**1-Pr**	CH_3_	11.17	–12.64	–13.31	–10.88
		THF-β		48.50	47.82	54.83
		THF-α		100.18	95.78	105.23
	**1-Nd**	CH_3_	23.94	–4.87	–8.22	–0.36
		THF-β		18.41	15.20	21.29
		THF-α		37.15	27.24	30.96
	**1-Sm**	CH_3_	5.47	–0.65	–0.58	–1.08
		THF-β	26.15	2.47	2.57	2.50
		THF-α	78.89	4.61	5.11	5.25
^29^Si	**1-Ce**	SiMe_3_	5.30	–5.74	–9.94	–1.93
	**1-Pr**	SiMe_3_	15.65	–26.19	–35.12	–15.27
	**1-Nd**	SiMe_3_	42.94	–9.84	–20.09	4.88
	**1-Sm**	SiMe_3_	0.52	–1.14	–0.46	–1.36
^31^P	**1-Ce**	P-Ln	616.7	–27.2	–52.7	–1.5
	**1-Pr**	P-Ln	1894.2	–107.8	–201.3	–96.0
	**1-Nd**	P-Ln	2570.1	–40.8	–172.8	–40.6
	**1-Sm**	P-Ln	–259.2	–4.4	–0.5	–6.1

A second method, intended to overcome the limitations of the first method and achieve a better agreement with experimental chemical shifts, is one where we can implicitly account for the nonpoint-dipolar nature of the spin system (i.e., the spatial delocalization of 4f spin density) and the spin density at the nuclei of interest (i.e., including the contact contribution): here, we employ the van den Heuvel and Soncini method^26^ in the Hyperion program.^87^ Using the existing CASSCF-SO calculations from above with the 4f-only active space, we observe only small changes in the calculated shifts (Table S22). This clearly indicates that while the 4f-only active space is sufficient to effectively approximate the localized magnetic anisotropy of the metal ion, it fails to model the electronic structure of the ligands well enough to accurately describe the contact shift or the effect of the spatial distribution of the unpaired electron density on the pseudocontact shift. The former of these issues is expected to be more significant for the ^31^P nuclei, which are directly bonded to the Ln ions.

Various more sophisticated active spaces were developed, focusing on the phosphorus atoms, in an attempt to improve calculated shifts by incorporating the phosphorus valence orbitals; however, only minor improvements to calculated ^31^P shifts were achieved (Table S22). The calculation in closest agreement with the experiment was a restricted active space configuration interaction spin–orbit (RASCI-SO) calculation using the orbitals generated by the above minimal CASSCF-SO calculations, where the phosphorus 1s, 2s, 3s, and 3p orbitals were placed in RAS1, the Ln 4f orbitals defined RAS2, and the 13 lowest-energy virtual orbitals comprised RAS3: this is just about the computational limit of CASSCF/RASSCF methods. Nonetheless, these calculations deliver ^31^P shifts that are still far from the experiment, highlighting the difficulty of capturing the details of electronic structure to which pNMR shifts are so sensitive to. It can also be noted that solution ^31^P NMR spectroscopy is particularly sensitive to factors such as temperature as well as various stereoelectronic effects, which are often not captured in the calculation of ^31^P chemical shifts when starting from the crystal structure.^88,89^ Therefore, small differences in structure in both solution and the ss can lead to significant changes in chemical shifts.

Surprisingly, we found large changes in the calculated shifts of other non-^31^P nuclei when these calculations were performed with larger active spaces on the phosphorus atoms. The RASCI-SO method that improved shifts for ^31^P also improved shifts for ^13^C and ^29^Si, having a larger effect relative to the magnitude of the experimental shift than that for ^31^P. A potential explanation is that the carbon and silicon atoms, separated from the central ion by more bonds than the phosphorus atoms in all cases, have a smaller contact shift contribution relative to their total pNMR shift than for phosphorus. Hence, any improvement in the modeling of the spin-dipolar interaction (i.e., of the spatial variation of the unpaired electron density that leads to the pseudocontact shift with a 1/r^3^ dependence) may be having a significant effect in these cases. This is consistent with the methyl ^1^H being considerably more distal and the only nucleus for which the point-dipole assumption seems to hold in **1-Ln**, showing no significant changes upon an increase in the active space.

## Conclusions

We have modified synthetic protocols for the preparation of solvated Ln(III) *tris*-(*bis*-trimethylsilyl)phosphide complexes to expand the [Ln{P(SiMe_3_)_2_}_3_(THF)_2_] series to Ln = La, Ce, Pr, Nd, Sm, and Tm.^37,38^ The unexpected observation of resonances in both the solution and solid-state ^31^P NMR spectra for this family of complexes for Ln = La, Ce, Pr, Nd, and Sm has enabled the first systematic study of ^31^P chemical shifts for paramagnetic f-block ions. Single-crystal XRD showed that these complexes adopt distorted trigonal bipyramidal coordination geometries in the ss, with three equatorial phosphides and two axially bound THF molecules, and NMR data obtained on solutions of these complexes in aromatic solvents indicated that these structures are retained in the solution. Uniquely, we were able to extract the CSAs from signals observed in ^31^P MAS NMR spectra of ss samples and correlate these data with the paramagnetism of the Ln and pyramidalization of the P atoms. While DFT calculations on the diamagnetic La(III) complex predicted ^31^P chemical shift parameters that were in excellent agreement with the values extracted from ^31^P MAS NMR spectra, CASSCF-SO calculations on paramagnetic Ce(III), Pr(III), Nd(III), and Sm(III) homologues could only effectively reproduce ^1^H NMR chemical shifts for these complexes. We find diminishing agreement with the experiment for calculated ^13^C, ^29^Si, and ^31^P pNMR chemical shifts as the distances between Ln(III) ions and ligands decrease across the **1-Ln** series; this highlights the current limitations of these methods for nuclei where contact shift contributions dominate.

## Experimental Section

### General

All manipulations were conducted under argon with the strict exclusion of oxygen and water by using Schlenk line and glovebox techniques. [LnI_3_(THF)*_*x*_*]^43^ and KP(SiMe_3_)_2_^44^ were synthesized according to literature procedures, and **1-Nd** was prepared by modification of literature procedures.^38^ Diethyl ether and toluene were purged with nitrogen and passed through columns containing alumina catalyst and molecular sieves, and hexane was dried by refluxing over potassium; these solvents were degassed, refilled with argon, and stored over a potassium mirror before use. For NMR spectroscopy, C_6_D_6_ was dried by refluxing over K and vacuum transferred and degassed by three freeze–pump–thaw cycles before use. Elemental analysis (C, H) was carried out by Mr. Martin Jennings and Mrs. Anne Davies at the Microanalytical service, Department of Chemistry, the University of Manchester. ATR-IR spectra were recorded on microcrystalline powders on a Bruker Alpha spectrometer with a Platinum-ATR module. UV–vis-NIR spectra were recorded on a PerkinElmer Lambda 750 spectrometer on 2 mM toluene solutions in 1 cm path length Youngs tap-appended cuvettes and were corrected to a toluene reference cell.

Single crystals suspended in Fomblin on a Micromount were examined by using Rigaku FR-X, Rigaku Synergy-S, and Rigaku Supernova diffractometers variously equipped with HE6000Hypix and CCD Eos detectors and graphite-monochromated Cu Kα (λ = 1.54178 Å) or Mo Kα radiation (λ = 0.71073 Å). Intensities were integrated from data recorded on 1° (**1-Ln**) frames by ω rotation. Cell parameters were refined from the observed positions of all of the strong reflections in each data set. A Gaussian grid face-indexed was used to correct for X-ray absorption.^90^ The structures were solved using SHELXT;^91^ the data sets were refined by full-matrix least-squares on all unique *F*^2^ values,^92^ with anisotropic displacement parameters for all non-hydrogen atoms, and with constrained riding hydrogen geometries; Uiso(H) was set at 1.2 (1.5 for methyl groups) times Ueq of the parent atom. The largest features in final difference syntheses were close to those of heavy atoms and were of no chemical significance. CrysAlisPro^90^ was used for control and integration, and SHELX^91,92^ was employed through OLEX2^93^ for structure solution and refinement. ORTEP-3^94^ and POV-Ray^95^ were employed for molecular graphics.

Powder XRD data were obtained on small batches of microcrystalline **1-Ln** that were suspended in Fomblin oil to prevent sample decomposition from oxygen and water. These samples were mounted on a Micromount and placed on a goniometer head under a cryostream to cool the sample to 100 K, freezing the Fomblin to suspend the crystallites for the duration of the experiment. The PXRD data were measured on a Rigaku FR-X diffractometer, operating in powder diffraction mode by using Cu Kα radiation (λ = 1.5418 Å) with a Hypix-6000HE detector and an Oxford Cryosystems nitrogen flow gas system. Data were collected between 3 and 70° θ, with a detector distance of 150 mm and a beam divergence of 1.0 mRad.^96^ X-ray data were collected using CrysAlisPro.^90^ For data processing, the instrument was calibrated using LaB_6_ as standard. Then, X-ray data were reduced and integrated using CrysAlisPro.^90^ Peak hunting and unit cell indexing were performed using the TOPAS software.^97^ Le Bail profile analysis was performed using the JANA2020 software.^98^ Some samples suffer from high background scatter from the Fomblin YR-1800 oil used due to the ratio of powder to oil. Qualitatively, the Le Bail refinements match the experimental data.

Solution NMR spectra were recorded on a Bruker AVIII HD 400 spectrometer operating at 400.07 (^1^H), 100.60 (^13^C{^1^H}), 79.48 (^29^Si DEPT90), and 161.98 (^31^P{^1^H}) MHz. Solid-state NMR spectra were recorded using a Bruker AVIII 9.4 T spectrometer equipped with a 4 mm HFXY MAS probe and a 2.5 mm HX MAS probe (162.03 (^31^P) MHz). Experiments were acquired at ambient temperature using various MAS frequencies to extract CSA parameters and isotropic shifts. For the frequencies employed (7 to 20 kHz), the sample temperature was determined using an external reference of KBr to be 300 ± 9 K; note that for paramagnetic **1-Ln**, the chemical shift will depend on the temperature. Samples were packed into 4 or 2.5 mm o.d. zirconia rotors in a glovebox and were sealed with Kel-F or Vespel rotor caps, respectively. The ^31^P (π/2)-pulse durations were 4 and 2.5 μs for the 4 and 2.5 mm rotors, respectively. For **1-Pr** and **1-Nd**, WCPMG-MAS NMR spectra were recorded to excite the required large bandwidth.^62^ 11 rotor-synchronized WURST echoes were recorded, the WURST sweep width was 6000 kHz with a shape parameter, *N*, of 20 for the WURST pulses of 100 kHz RF amplitude. To obtain the isotropic chemical shifts for **1-Pr** and **1-Nd**, 2D pjMATPASS experiments were recorded and sheared, with summed 1D projections extracted (see Supporting Information Figures S43b and S44b).^99^ See Table S7 for further details. Spectral simulations were performed in the solid line-shape analysis (SOLA) module v2.2.4 in Bruker TopSpin v3.6.3 and using EXPRESS 2.0^100^ and WSolids1 ver 1.21.7.^101^^1^H, ^13^C, and ^29^Si NMR spectra were referenced to SiMe_4_, and ^31^P NMR spectra were referenced to 85% H_3_PO_4_.

Magnetic data were collected on a Quantum Design MPMS3 superconducting quantum interference device (SQUID) magnetometer using doubly recrystallized powdered samples. Samples were prepared in an NMR tube containing a finely ground material with eicosane as a restraint, which was then flame-sealed under *vacuo*. The ampules were mounted in plastic straws, held in place with diamagnetic tape. Samples were carefully checked for purity and data reproducibility between several independently prepared batches for each compound examined. For **1-Ce**, slow thermalization was observed below 50 K, and care was taken to ensure complete thermalization of the sample before each data point was measured. Measurements were corrected for the contribution of the blank sample holders (flame-sealed Wilmad NMR tube and straw) and eicosane matrix, corrected for the shape of the sample using the MPMS3 Geometry Simulator (correction factors 0.974–1.075) and corrected for the diamagnetic contribution, approximated as the molecular weight multiplied by 0.5 × 10^–6^ cm^3^ K mol^–1^.^102^ Variable-temperature magnetic susceptibility was collected under an applied field (**1-Ce**: 0.5 T, **1-Pr**: 0.1 T, **1-Nd**: 0.1 T, **1-Sm**: 1 T) using either DC scan mode with a 40 mm scan length and 6 s scan time (**1-Ce**, **1-Nd**) or VSM mode with 5 mm amplitude and 2 s averaging time (**1-Pr**, **1-Sm**). Isothermal magnetization measurements were performed in DC scan mode with a 40 mm scan length and 6 s scan time for all samples.

Continuous wave (CW) X-band EPR spectra were recorded with a Bruker EMXPlus spectrometer with a 1.8 T electromagnet and Stinger closed-cycle helium gas cryostat. Polycrystalline samples of **1-Ce** and **1-Nd** were sealed in quartz X-band EPR tubes under vacuum; samples were lightly ground with a mortar and pestle under inert atmosphere to reduce the amount of sample decomposition, but we note that some effects due to polycrystallinity remain in the **1-Nd** spectra (identified by comparing two sample rotations at ∼90 deg to one another). Spectra were obtained at base temperature (7–16 K). The field was corrected by using a strong pitch sample (*g* = 2.0028). Spectra were simulated in EasySpin 6.0.0-dev.48 using the pepper function.^66^ The ground doublet was simulated as an effective *S* = 1/2 with axial *g*-values (*g* = [*g*_*z*_, *g*_*xy*_, *g*_*xy*_]) and *g*-strains to phenomenologically account for all anisotropic line-broadening effects. For Nd, a hyperfine coupling on the *z*-component was included in the model (*A* = [*A*_*z*_, 0, 0]), with the *A* axis assumed to be collinear with the *g*-axis. The hyperfine coupling constants are defined for the most abundant isotopes and scaled for other isotopes based on nuclear *g*-factors. Values used in the simulation are reported in Table S9.

DFT calculations for **1-La** were performed using ORCA v5.0.^67^ NMR parameter calculations and geometry optimizations were performed by using a variety of DFT methods, starting from the XRD structure. Reference chemical shielding was found from an optimized structure of H_3_PO_4_ using each method. In all cases, scalar relativistic ZORA^103^ was employed alongside the GD3 dispersion correction^104^ and the def2-TZVP basis set^105^ in its relativistically contracted form. The SARC form^106^ of this basis set was used for La. The DFT methods used were BP86^107,108^ and B3LYP^82,109^ with a range of exact exchange contributions. The PCM solvent model^110^ was investigated, but was found to be ineffective (see Supporting Information Table S15). NBO calculations used NBO 6.0^111^ in Orca. They were carried out using the DFT methodology described above with B3LYPHF20 and B3LYPHF30.

Multiconfigurational electronic structure theory calculations were performed on **1-Ce**, **1-Pr**, **1-Nd**, and **1-Sm** in OpenMolcas version 23.02.^83^ The molecular geometries from single-crystal XRD structures were used with no optimization, selecting a single molecule from the asymmetric unit and taking the largest disorder component as the only one. Integrals were performed in the SEWARD module using basis sets from the ANO-RCC library^112,113^ with VTZP quality on the metal atom, VDZP quality on the P and O atoms, and VDZ quality on all other atoms, employing the second-order DKH transformation. Cholesky decomposition of the two-electron integrals with a threshold of 10^–8^ was performed to save disk space and reduce computational demand. The molecular orbitals (MOs) were optimized in state-averaged CASSCF calculations in the RASSCF module, with a CAS(*n*,7) calculation (**1-Ce**: *n* = 1; **1-Pr**: *n* = 2; **1-Nd**: *n* = 3; **1-Sm**: *n* = 5) where the active space was the seven 4f orbitals. For **1-Ce**, the MOs were averaged over the seven lowest seven doublets. For **1-Pr**, the MOs were averaged over the 21 lowest 21 triplets and the 28 lowest 28 singlets. For **1-Nd**, the MOs were averaged over the 35 lowest-order quartets and the 112 lowest-order doublets. For **1-Sm**, the MOs were averaged over the lowest 21 sextets, the lowest 224 quartets, and the lowest 490 doublets. The wave functions obtained from these CASSCF calculations were then mixed by spin orbit coupling in the RASSI module, where all states were included for **1-Ce**, **1-Pr**, and **1-Nd**, and for **1-Sm**, all 21 sextets, 128 of the quartets, and 130 of the doublets were included. SINGLE_ANISO was used to decompose the resulting spin–orbit wave functions into the CF Hamiltonian formalism.^114^ Diamond was employed for molecular graphics.^115^

For pNMR calculations, a temperature of 297 K was used for all analyses, and shifts were averaged over all atoms in each environment. An ANO-RCC-VTZP basis set was employed for the Ln ion, an ANO-RCC-VDZP basis set for the ligand atoms directly coordinated to the Ln ion, and an ANO-RCC-VDZ basis set for all other atoms.^116^ Two-electron integrals were decomposed using the Cholesky method, with a threshold of 10^–8^. The PCM solvent model^110^ was used for some calculations using the magnetic susceptibility method^86^ but was found to have an insignificant effect (Table S21). Geometry optimizations for **1-Ln** were carried out in Gaussian 16^117^ using DFT, starting from XRD structures. These used the PBE exchange-correlation functional^118^ with the GD3 dispersion correction^104^ and a cc-pVDZ basis set.^119^ An f-in-core pseudopotential was used for the Ln ion.^120^ Calculated shifts for the XRD and optimized geometries, found using the magnetic susceptibility method, are compared in Table S21. These differed, but neither gave reliably better agreement with experiment than the other. The XRD structures were used for all of the further calculations.

Initially, CASSCF-SO calculations were performed on each molecule, with the relevant number of electrons in the seven 4f orbitals as the active space. The RAS method was used for further calculations with RAS2 always being the same as the initial CAS active space. The CASSCF-SO orbitals were used for the RASCI-SO calculations with different active spaces. RAS1 was varied, with RAS3 always being the 13 lowest-energy virtual orbitals. The results of these RASCI-SO calculations are summarized in Table S22, with the implementation of the van den Heuvel equation^26^ in the Hyperion^87^ software being used to calculate paramagnetic shifts. The phosphorus 1s and 2s orbitals were core-like and easily identified, but the 3s and 3p orbitals were involved in bonding. The molecular orbitals used to represent these were those with the largest contributions from them. In all cases, these had a significantly larger contribution from the relevant atomic orbital than from any other atomic orbital. There were also no other molecular orbitals with such a large contribution from that atomic orbital, making the identification unambiguous.

RASSCF-SO calculations were then carried out with RAS1 as the three highest-energy phosphorus 3p orbitals and RAS3 as the three lowest-energy virtual orbitals. These suffered from rotation of the RAS1 orbitals during the SCF procedure to become metal 4d and 5p orbitals. Further RASSCF-SO calculations were carried out with RAS1 as all nine phosphorus 3p orbitals and RAS3 as the nine lowest-energy virtual orbitals. These saw significantly less rotation to metal orbitals, and in the case of **1-Nd**, all RAS1 orbitals remained predominantly phosphorus 3p in character. The orbitals from **1-Nd** RASSCF-SO were used for a RASCI-SO calculation, with RAS1 as the phosphorus 3s and 3p orbitals and RAS3 as the 12 lowest-energy virtual orbitals. pNMR shifts calculated from the results of the RASSCF-SO calculations with all of the phosphorus 3p orbitals as RAS1 and this RASCI-SO calculation are shown in Table S23.

### General Procedure for the Synthesis of 1-La

To a precooled (−78 °C) suspension of [LnI_3_(THF)*_*x*_*] (Ln = La, Ce, Pr, *x* = 4; Ln = Nd, Sm, *x* = 3.5) in diethyl ether (10 mL), a suspension of KP(SiMe_3_)_2_ (3 equiv) in diethyl ether (10 mL) was added dropwise. The resultant reaction mixture was stirred for 1 h at −78 °C before being allowed to warm to room temperature over 20 min. All volatiles were removed *in vacuo*, and the solid was extracted with hexane (30 mL) and filtered. The filtrate was concentrated to ca. 5 mL and stored at −30 °C to yield crystals, which were isolated and dried *in vacuo* to afford the title compound.

#### [La{P(SiMe_3_)_2_}_3_(THF)_2_] (1-La)

Prepared according to the general procedure with [LaI_3_(THF)_4_] (0.8083 g, 1.00 mmol) and KP(SiMe_3_)_2_ (0.6494 g, 3.00 mmol) to give orange crystals of **1-La**. Yield = 0.2470 g, 0.37 mmol, 37%. Anal. calcd (%) for C_26_H_70_O_2_P_3_Si_6_La: C, 38.31 H, 8.66. Found (%): C, 37.05; H, 8.23. ^1^H NMR (400 MHz, C_6_D_6_, 298 K): δ 0.30 (s, 54H, PSi(C*H*_3_)_3_), 1.22 (br m, 8H, THF–C*H*_2_), 4.14 (br m, 8H, THF–C*H*_2_O). ^13^C{^1^H} NMR (101 MHz, C_6_D_6_, 298 K): δ 7.25 (PSi(*C*H_3_)_3_), 25.13 (THF–C*H*_2_), 72.96 (THF–C*H*_2_O). ^29^Si DEPT90 NMR (79 MHz, C_6_D_6_, 298 K): δ 2.66, (d, ^1^*J*_SiP_ = 22.4 Hz, *Si*Me_3_). ^31^P{^1^H} NMR (162 MHz, C_6_D_6_, 298 K): δ – 113.0 (br, fwhm ≈1150 Hz, *P*-La). FTIR (ATR, microcrystalline): υ̃/cm^–1^: 2945 (m), 2887 (m), 1441 (w), 1397 (m), 1239 (s), 1013 (s), 815 (s). NIR-UV–vis (2 mM, toluene) υ̃/cm^–1^: no maxima observed.

#### [Ce{P(SiMe_3_)_2_}_3_(THF)_2_] (1-Ce)

Prepared according to the general procedure with [CeI_3_(THF)_4_] (0.8095 g, 1.00 mmol) and KP(SiMe_3_)_2_ (0.6494 g, 3.00 mmol) to give yellow/green crystals of **1-Ce**. Yield = 0.3449 g, 0.51 mmol, 51%. Anal. calcd (%) for C_26_H_70_O_2_P_3_Si_6_Ce: C, 38.25; H, 8.64. Found (%): C, 37.52; H, 8.94. ^1^H NMR (400 MHz, C_6_D_6_, 298 K): δ – 2.17 (s, 54H, PSi(C*H*_3_)_3_), 1.02 (br, 8H, fwhm ≈70 Hz, THF–C*H*_2_), 8.33 (br, 8H, fwhm ≈180 Hz, THF–C*H*_2_O). ^13^C{^1^H} NMR (101 MHz, C_6_D_6_, 298 K): δ 7.93 (PSi(*C*H_3_)_3_), THF resonances not observed. ^29^Si DEPT90 NMR (79 MHz, C_6_D_6_, 298 K): δ 5.30, (br, *Si*Me_3_). ^31^P{^1^H} NMR (162 MHz, C_6_D_6_, 298 K): δ 616.7 (br, fwhm ≈350 Hz, *P*-Ce). FTIR (ATR, microcrystalline): υ̃/cm^–1^: 2945 (m), 2887 (m), 1442 (w), 1397 (m), 1239 (s), 1015 (s), 819 (s). NIR-UV–vis (2 mM, toluene) υ̃/cm^–1^: 22,000 (ε = 750 M^–1^ cm^–1^), 19,500 (ε = 180 M^–1^ cm^–1^).

#### [Pr{P(SiMe_3_)_2_}_3_(THF)_2_] (1-Pr)

Prepared according to the general procedure with [PrI_3_(THF)_4_] (0.8130 g, 1.00 mmol) and KP(SiMe_3_)_2_ (0.6494 g, 3.00 mmol) to give yellow/green crystals of **1-Pr**. Yield = 0.2671 g, 0.33 mmol, 33%. Anal. calcd (%) for C_26_H_70_O_2_P_3_Si_6_Pr: C, 38.22; H, 8.63. Found (%): C, 36.82; H, 8.62. ^1^H NMR (400 MHz, C_6_D_6_, 298 K): δ – 6.83 (s, 54H, PSi(C*H*_3_)_3_), 23.50 (br, 8H, fwhm ≈450 Hz,THF–C*H*_2_), 48.12 (br, 8H, fwhm ≈960 Hz, THF–C*H*_2_O). ^13^C{^1^H} NMR (101 MHz, C_6_D_6_, 298 K): δ 11.17 (PSi(*C*H_3_)_3_), THF resonances not observed. ^29^Si DEPT90 NMR (79 MHz, C_6_D_6_, 298 K): δ 15.65, (br, *Si*Me_3_). ^31^P{^1^H} NMR (162 MHz, C_6_D_6_, 298 K): δ 1894.2 (br, fwhm ≈550 Hz, *P*-Pr). FTIR (ATR, microcrystalline): υ̃/cm^–1^: 2947 (m), 2887 (m), 1446 (w), 1395 (m), 1237 (s), 1013 (s), 813 (s). NIR-UV–vis (2 mM, toluene) υ̃/cm^–1^: no maxima observed.

#### [Nd{P(SiMe_3_)_2_}_3_(THF)_2_] (1-Nd)^38^

Prepared according to the general procedure with [NdI_3_(THF)_3.5_] (0.7774 g, 1 mmol) and KP(SiMe_3_)_2_ (0.6494 g, 3 mmol) to give large green crystals of **1-Nd**. Yield = 0.5085 g, 0.7500 mmol, 75%. Anal. calcd (%) for C_26_H_70_O_2_P_3_Si_6_Nd: C, 38.06; H, 8.60. Found (%): C, 36.30; H, 8.79. ^1^H NMR (400 MHz, C_6_D_6_, 298 K): δ – 2.24 (s, 54H, PSi(C*H*_3_)_3_), 11.75 (br, 8H, fwhm ≈130 Hz, THF–C*H*_2_), 23.52 (br, 8H, fwhm ≈360 Hz, THF–C*H*_2_O). ^13^C{^1^H} NMR (101 MHz, C_6_D_6_, 298 K): δ 23.94 (PSi(*C*H_3_)_3_), THF resonances not observed. ^29^Si DEPT90 NMR (79 MHz, C_6_D_6_, 298 K): δ 42.94 (s, *Si*Me_3_). ^31^P{^1^H} NMR (162 MHz, C_6_D_6_, 298 K): δ 2570.1 (br, fwhm ≈1100 Hz, *P*-Nd). FTIR (ATR, microcrystalline): υ̃/cm^–1^: 2947 (m), 2887 (m), 1438 (w), 1399 (m), 1237 (s), 1087 (w), 1013 (s), 813 (s). NIR-UV–vis (2 mM, toluene) υ̃/cm^–1^: 18,650 (ε = 50 M^–1^ cm^–1^), 16,100–17,300 (ε = 190 M^–1^ cm^–1^).

#### [Sm{P(SiMe_3_)_2_}_3_(THF)_2_] (1-Sm)

Prepared according to the general procedure with [SmI_3_(THF)_3.5_] (0.8200 g, 1 mmol) and KP(SiMe_3_)_2_ (0.6494 g, 3 mmol) to give pink/purple crystals of **1-Sm**. Yield = 0.2338 g, 0.2780 mmol, 28%. Anal. calcd (%) for C_26_H_70_O_2_P_3_Si_6_Sm: C, 37.78; H, 8.54. Found (%): C, 36.37; H, 8.50. ^1^H NMR (400 MHz, C_6_D_6_, 298 K): δ 0.09 (s, 54H, PSi(C*H*_3_)_3_), 2.35 (br, 8H, fwhm ≈10 Hz, THF–C*H*_2_), 6.11 (br, 8H, fwhm ≈20 Hz, THF–C*H*_2_O). ^13^C{^1^H} NMR (101 MHz, C_6_D_6_, 298 K): δ 5.47 (PSi(*C*H_3_)_3_), 26.15 (THF-*C*H_2_), 78.89 (THF-*C*H_2_O). ^29^Si DEPT90 NMR (79 MHz, C_6_D_6_, 298 K): δ 0.52, (s, *Si*Me_3_). ^31^P{^1^H} NMR (162 MHz, C_6_D_6_, 298 K): δ – 259.2 (br, fwhm ≈1500 Hz, *P*-Sm). FTIR (ATR, microcrystalline): υ̃/cm^–1^: 2949 (m), 2887 (m), 1448 (w), 1398 (m), 1237 (s), 1013 (s), 816 (s). NIR-UV–vis (2 mM, toluene) υ̃/cm^–1^: 17,800 (ε = 500 M^–1^ cm^–1^).

## Data Availability

Research data files supporting this publication are available from FigShare at https://figshare.com/doi/10.6084/m9.figshare.26004523.

## Supporting Information Available

All spectroscopic and magnetic data for novel compounds reported in this manuscript; additional experimental details; materials and methods (PDF)

## Accession Codes

CCDC 2284755–2284759 contain the supplementary crystallographic data for this paper. These data can be obtained free of charge via www.ccdc.cam.ac.uk/data_request/cif, or by emailing data_request@ccdc.cam.ac.uk, or by contacting The Cambridge Crystallographic Data Centre, 12 Union Road, Cambridge, CB2 1EZ, UK; fax: + 44 1223 336033.

## Notes

A preprint of this article was previously deposited on *ChemRxiv*.^121^
