# Supplementary material for: 31P NMR Chemical Shift Anisotropy in Paramagnetic Lanthanide Phosphide Complexes
Source: JACS Au. 2025 Feb 21;5(3):1196–212. doi: 10.1021/jacsau.4c01018 (PMC11937968; doi:10.1021/jacsau.4c01018)
Supplement: Supplementary file 1 — au4c01018_si_001.pdf [file au4c01018_si_001.pdf]

Electronic Supplementary Information

^{31}P NMR chemical shift anisotropy in paramagnetic lanthanide phosphide complexes

Jack Baldwin,[1] Katherine L. Bonham,[2] Toby R. C. Thompson,[1] Gemma K. Gransbury,[1] George F. S. Whitehead,[1] Iñigo J. Vitorica-Yrezabal,[1] Daniel Lee,[2,*] Nicholas F. Chilton[1,3,*] and David P. Mills[1,*]

[1]*Department of Chemistry, The University of Manchester, Oxford Road, Manchester, M13 9PL, U.K.*
[2]*Department of Chemical Engineering, The University of Manchester, Oxford Road, Manchester, M13 9PL, U.K.*
[3]*Research School of Chemistry, The Australian National University, Sullivans Creek Road, Canberra, ACT, 2601, Australia.*
**Email: daniel.lee@manchester.ac.uk; nicholas.chilton@anu.edu.au; david.mills@manchester.ac.uk*

Contents

1. **ATR-IR Spectroscopy: 1-Ln** S2
2. **Crystallographic Details: 1-Ln** S5
3. **Molecular Structures: 1-Ln** S8
4. **Powder X-ray Diffraction: 1-Ln** S10
5. **Solution NMR Spectroscopy: 1-Ln** S21
6. **Solid-state NMR Spectroscopy: 1-Ln** S32
7. **UV-Vis-NIR Spectroscopy: 1-Ln** S41
8. **Magnetism: 1-Ce, 1-Pr, 1-Nd, 1-Sm** S44
9. **EPR Spectroscopy: 1-Ce, 1-Nd** S48
10. **Density Functional Theory (DFT) Calculations** S49
11. **Complete Active Space Self-Consistent Field (CASSCF) Calculations** S63
12. **pNMR Calculations** S69
13. **References** S72

1. ATR-IR Spectroscopy: 1-Ln

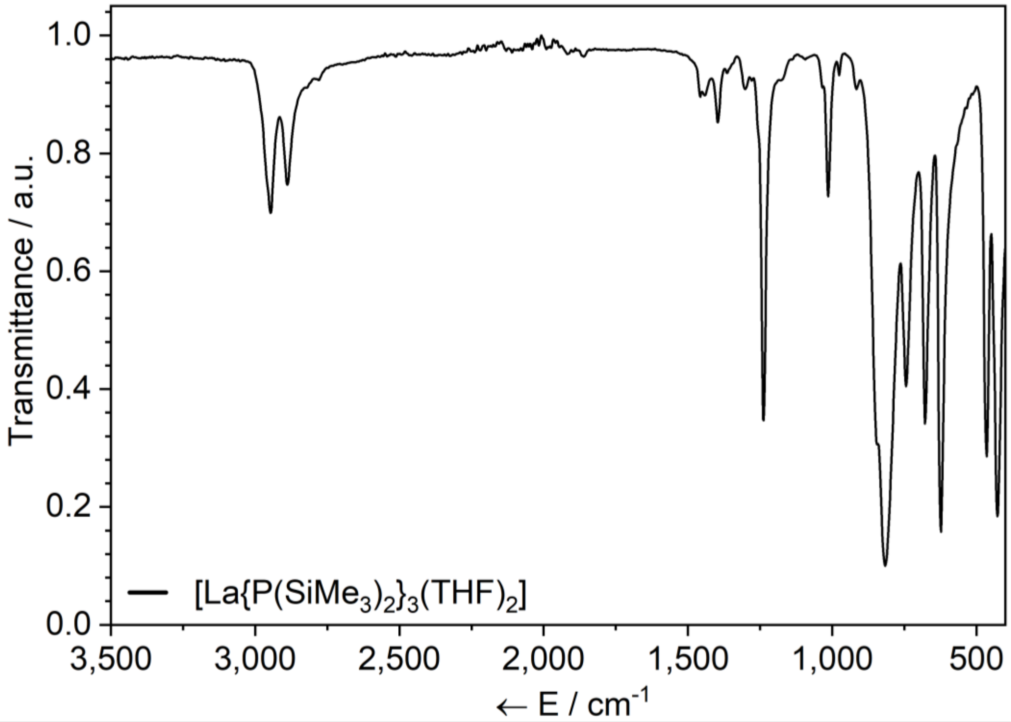

Figure S1. ATR-IR spectrum of **1-La** between 398-4000 cm^{-1}.

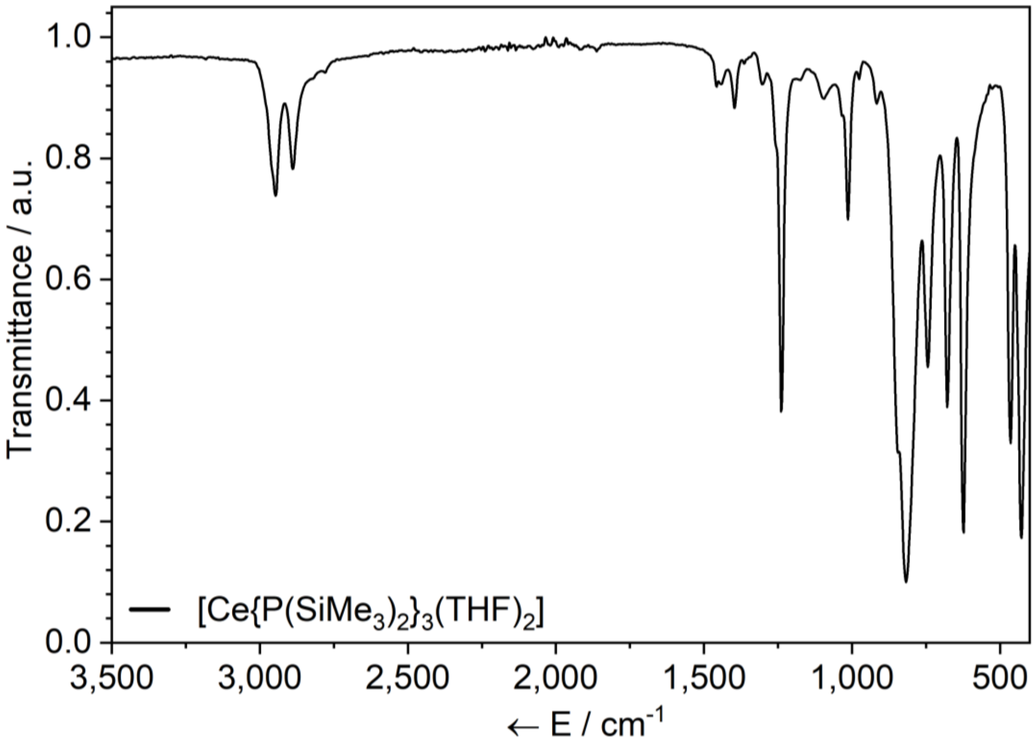

Figure S2. ATR-IR spectrum of **1-Ce** between 398-4000 cm^{-1}.

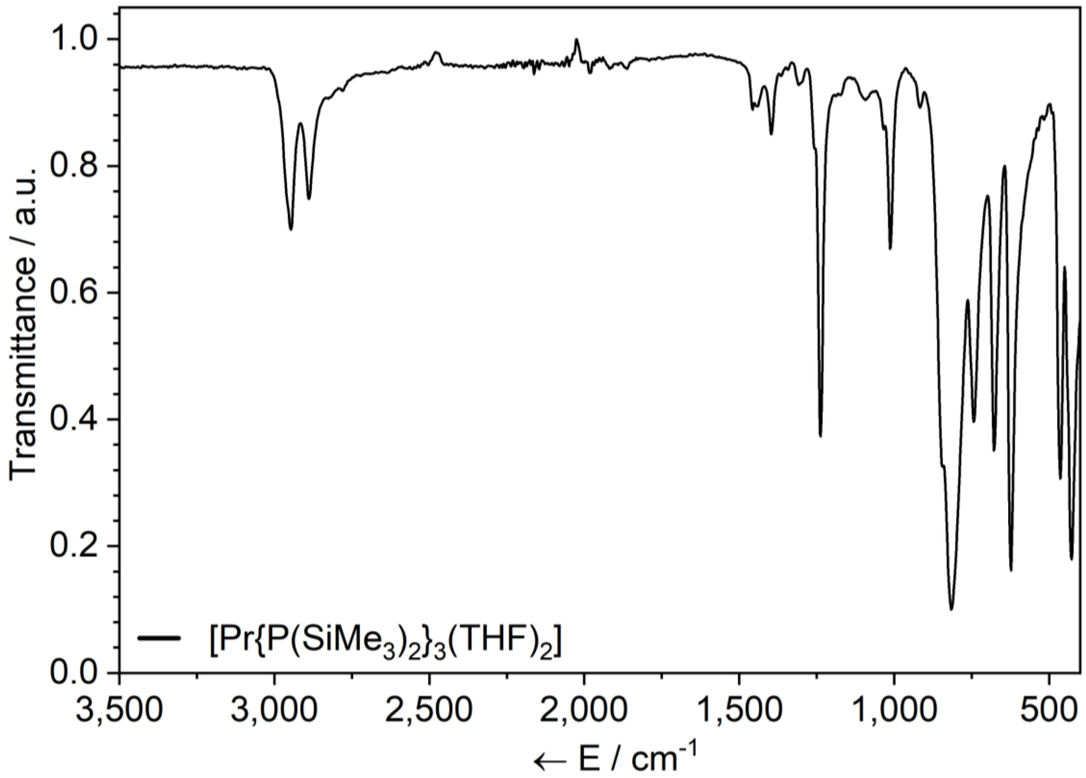

Figure S3. ATR-IR spectrum of **1-Pr** between 398-4000 cm^{-1}.

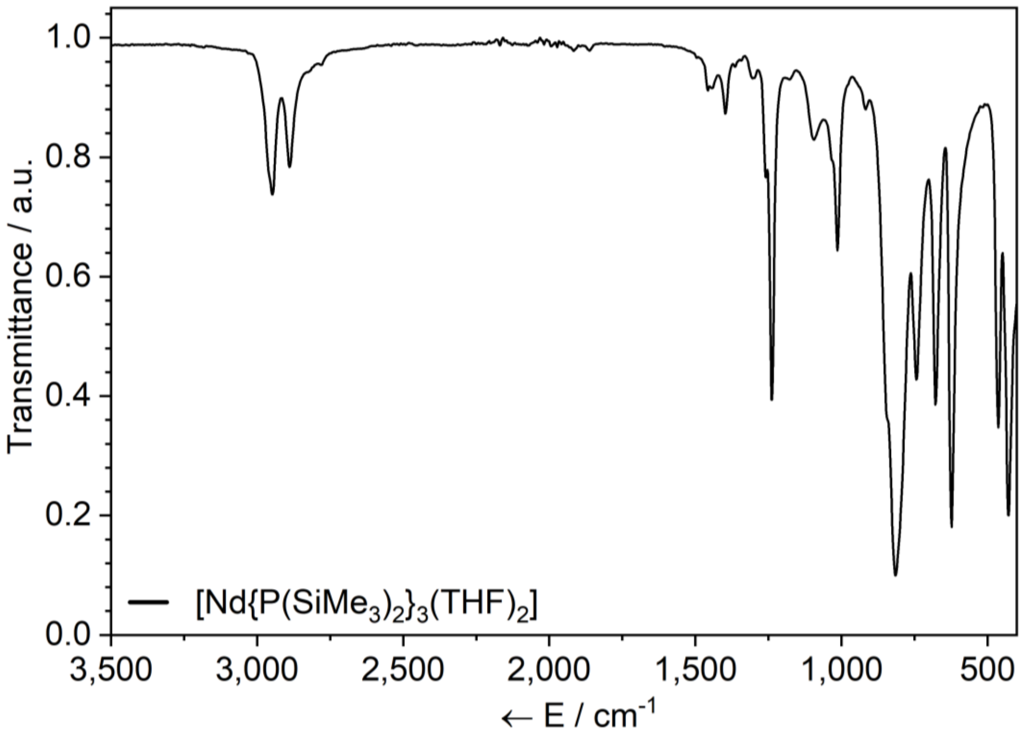

Figure S4. ATR-IR spectrum of **1-Nd** between 398-4000 cm^{-1}.

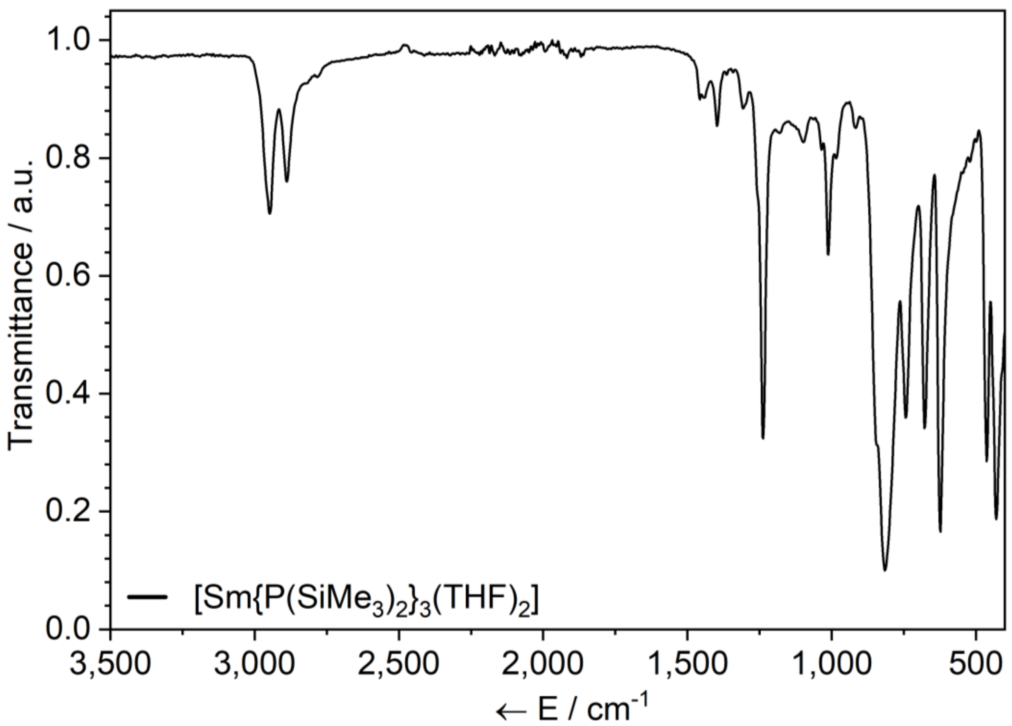

Figure S5. ATR-IR spectrum of **1-Sm** between 398-4000 cm^{-1}.

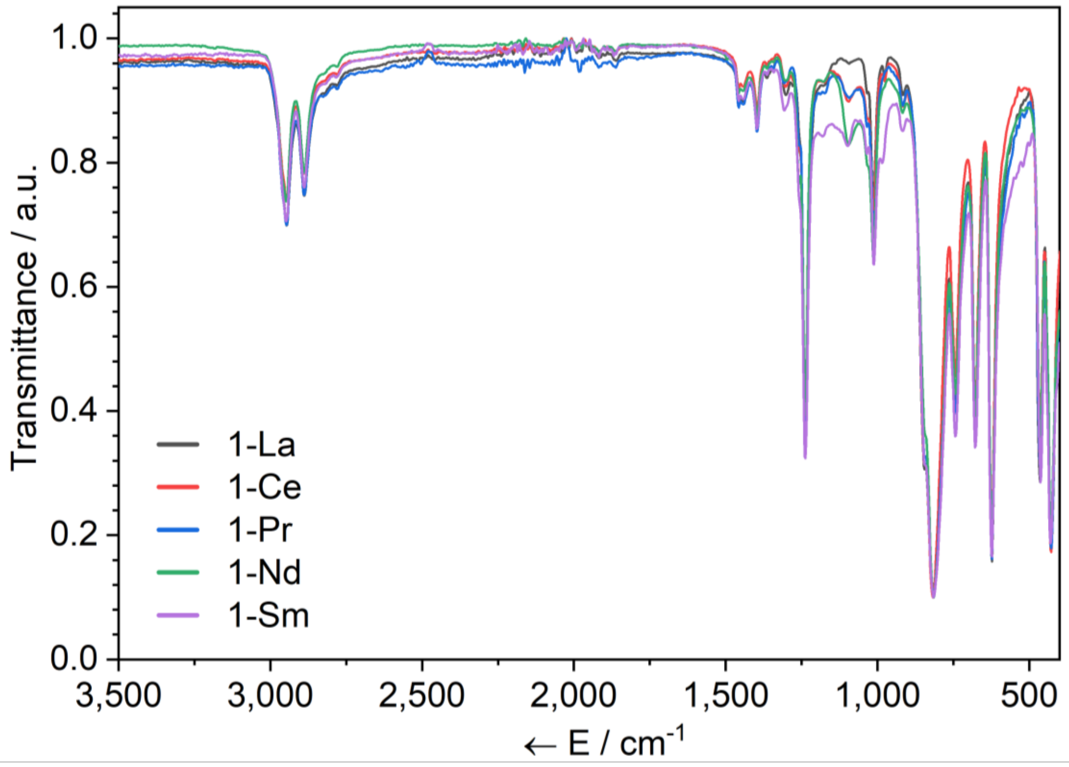

Figure S6. ATR-IR spectra of **1-La**, **1-Ce**, **1-Pr**, **1-Nd** and **1-Sm** between 398-4000 cm^{-1}.

2. Crystallographic Details: 1-Ln

Table S1. Crystallographic data for [Ln{P(SiMe$_3$)$_2$}$_3$(THF)$_2$] (**1-Ln**; Ln = La, Ce). [a]Conventional $R = \Sigma||F_o| - |F_c||/\Sigma|F_o|$; $R_w = [\Sigma w(F_o^2 - F_c^2)^2/\Sigma w(F_o^2)^2]^{1/2}$; $S = [\Sigma w(F_o^2 - F_c^2)^2$ /no. data – no. params)$]^{1/2}$ for all data.

	**1-La**	**1-Ce**
Formula	$C_{26}H_{70}LaO_2P_3Si_6$	$C_{26}H_{70}CeO_2P_3Si_6$
Fw	815.18	816.39
crystal size, mm	0.332 × 0.148 × 0.079	0.324 × 0.231 × 0.191
crystal system	Monoclinic	Monoclinic
space group	$P2_1/c$	$P2_1/c$
a, Å	17.2283(4)	17.58981(16)
b, Å	13.4889(2)	13.52717(14)
c, Å	19.8611(3)	39.0970(3)
α, °	90	90
β, °	103.585(2)	100.9419(9)
γ, °	90	90
V, Å^3	4486.41(15)	9133.62(15)
Z	4	4
ρ_{calc}, g/cm^3	1.207	1.187
μ, mm^{-1}	10.053	10.349
F(000)	1712	3432
no. of unique reflns	9056	18497
R, *Rwa* ($F^2 > 2\sigma(F^2)$)	0.0393, 0.1037	0.0395, 0.1070
S[a]	1.045	1.060
Rint	0.0425	0.0450
max., min. diff map, e Å^{-3}	1.065, –1.362	1.570, –1.917

Table S2. Crystallographic data for [Ln{P($SiMe_3$)$_2$}$_3$(THF)$_2$] (**1-Ln**; Ln = Pr, Nd). [a]Conventional $R = \Sigma||F_o| - |F_c||/\Sigma|F_o|$; $R_w = [\Sigma w(F_o^2 - F_c^2)^2/\Sigma w(F_o^2)^2]^{1/2}$; S = $[\Sigma w(F_o^2 - F_c^2)^2$ /no. data – no. params)]$^{1/2}$ for all data.

	**1-Pr**	**1-Nd**
Formula	$C_{26}H_{70}PrO_2P_3Si_6$	$C_{26}H_{70}NdO_2P_3Si_6$
Fw	817.18	820.51
crystal size, mm	0.393 × 0.200 × 0.132	0.352 × 0.194 × 0.095
crystal system	Monoclinic	Monoclinic
space group	$P2_1/c$	$P2_1/c$
a, Å	17.2349(8)	17.4728(2)
b, Å	13.4465(6)	13.47880(10)
c, Å	19.7410(11)	38.8791(4)
α, °	90	90
β, °	103.704(5)	100.9230(10)
γ, °	90	90
V, Å^3	4444.7(4)	8990.62(16)
Z	4	4
ρ_{calc}, g/cm^3	1.221	1.212
μ, mm^{-1}	1.386	11.501
F(000)	1720	3448
no. of unique reflns	10199	66434
R, *Rwa* ($F^2 > 2\sigma(F^2)$)	0.0603, 0.1397	0.0405, 0.1070
S[a]	1.041	1.016
Rint	0.0588	0.0897
max., min. diff map, e Å^{-3}	2.002, –1.435	0.619, –1.497

Table S3. Crystallographic data for [Ln{P(SiMe$_3$)$_2$}$_3$(THF)$_2$] (**1-Ln**; Ln = Sm). [a]Conventional $R = \Sigma||F_o| - |F_c||/\Sigma|F_o|$; $R_w = [\Sigma w(F_o^2 - F_c^2)^2/\Sigma w(F_o^2)^2]^{1/2}$; $S = [\Sigma w(F_o^2 - F_c^2)^2$ /no. data – no. params)]$^{1/2}$ for all data.

	**1-Sm**
Formula	$C_{26}H_{70}SmO_2P_3Si_6$
Fw	826.62
crystal size, mm	0.229 × 0.157 × 0.143
crystal system	Monoclinic
space group	$P2_1/c$
a, Å	17.3648(2)
b, Å	13.4530(2)
c, Å	38.6450(5)
α, °	90
β, °	100.8200(10)
γ, °	90
V, Å^3	8867.3(2)
Z	4
ρ_{calc}, g/cm^3	1.238
μ, mm^{-1}	1.615
F(000)	3464
no. of unique reflns	48311
R, *Rwa* ($F^2 > 2\sigma(F^2)$)	0.0503, 0.0727
S[a]	1.272
Rint	0.0510
max., min. diff map, e Å^{-3}	0.939, –0.634

3. Molecular Structures: 1-Ln

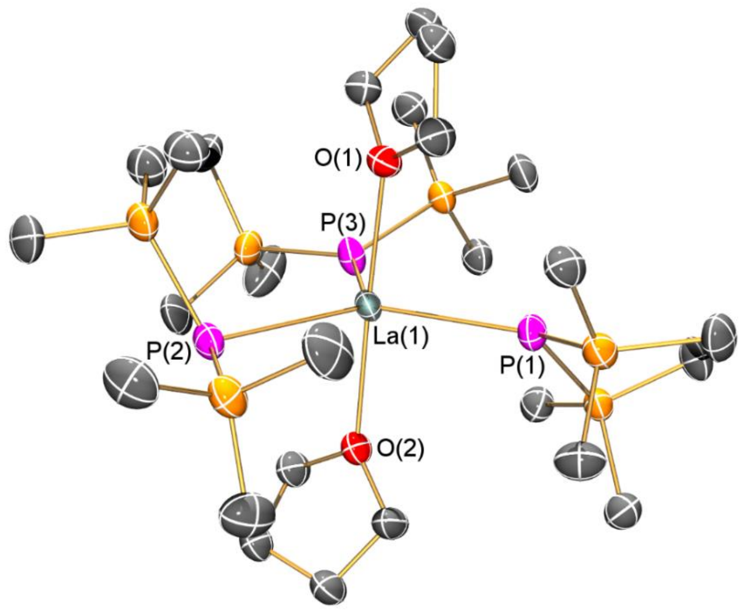

Figure S7. Solid state structure of **1-La**. Displacement ellipsoids set at 50% probability level, hydrogen atoms omitted for clarity.

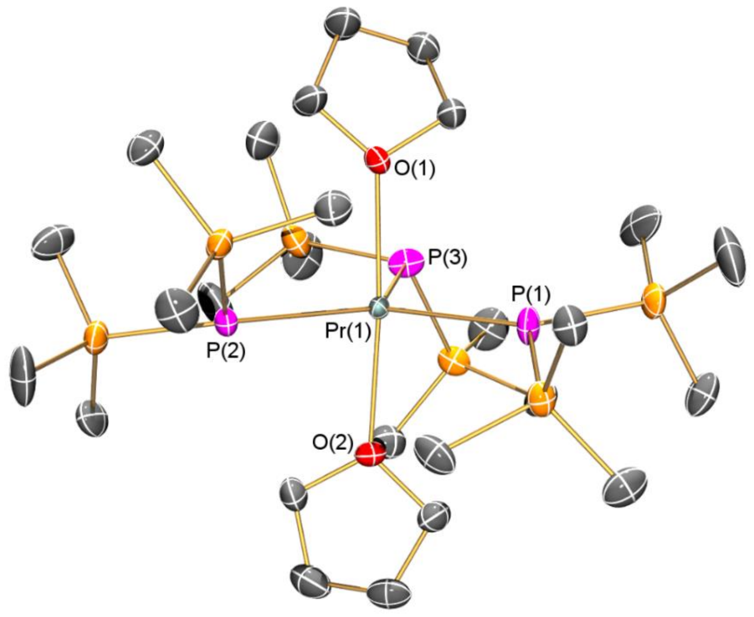

Figure S8. Solid state structure of **1-Pr**. Displacement ellipsoids set at 50% probability level, hydrogen atoms omitted for clarity.

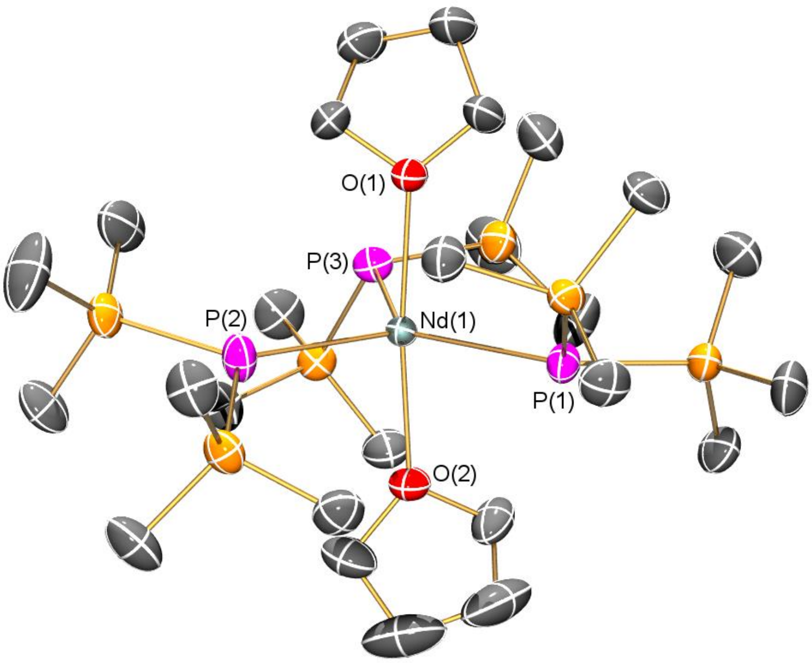

Figure S9. Solid state structure of **1-Nd**. Displacement ellipsoids set at 50% probability level, hydrogen atoms omitted for clarity.

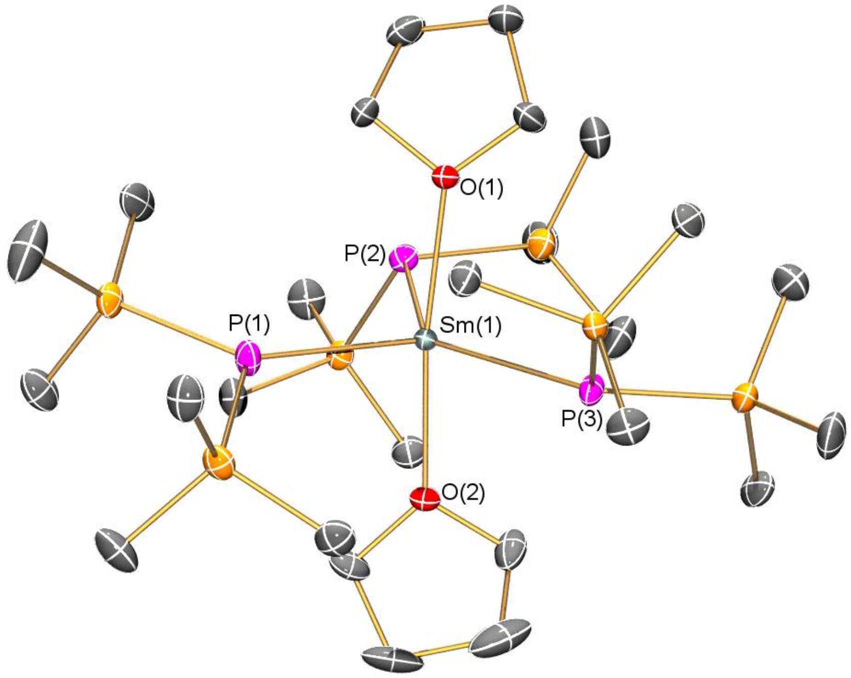

Figure S10. Solid state structure of **1-Sm**. Displacement ellipsoids set at 50% probability level, hydrogen atoms omitted for clarity.

4. Powder X-ray Diffraction: 1-Ln

(a) **(b)**

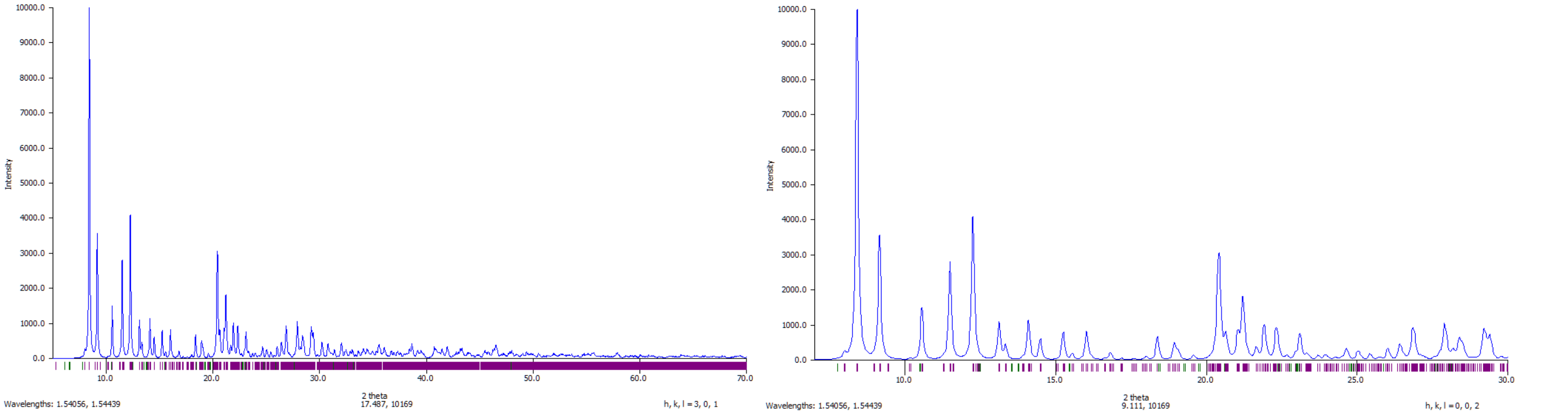

Figure S11. Calculated powder X-ray diffraction pattern of **1-La** derived from crystallographic model. (a) 5–70°, (b) 7–30°. *x*-axis 2θ (°), *y*-axis intensity (a.u.).

(a)

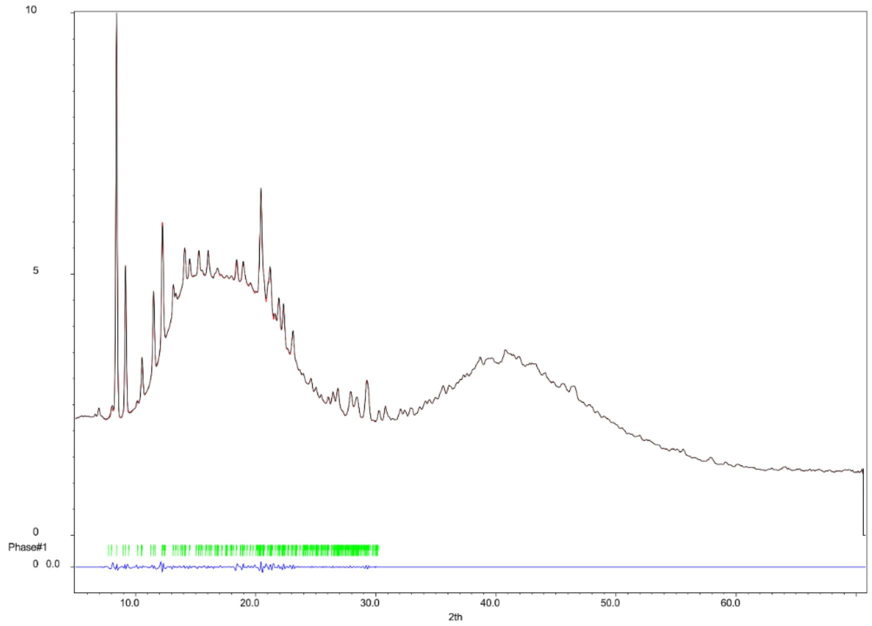

(b)

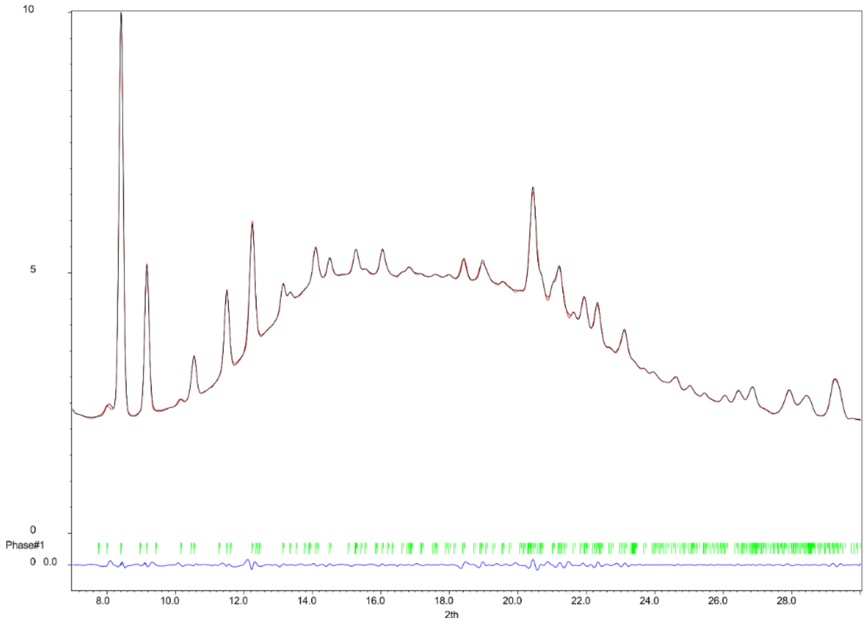

Figure S12. (a) 5–70° full range PXRD pattern of **1-La**, (b) Selected range 7–30° for Le Bail method profile fitting of **1-La**; experimental data (black), calculated fit from crystallographic parameters (red) and the difference (blue). Le Bail method profile fitting Rp = 0.37; wRp = 0.53. Single crystal unit cell values were used for the initial starting unit cell. *x*-axis 2θ (°), *y*-axis intensity (a.u.).

(a) (b)

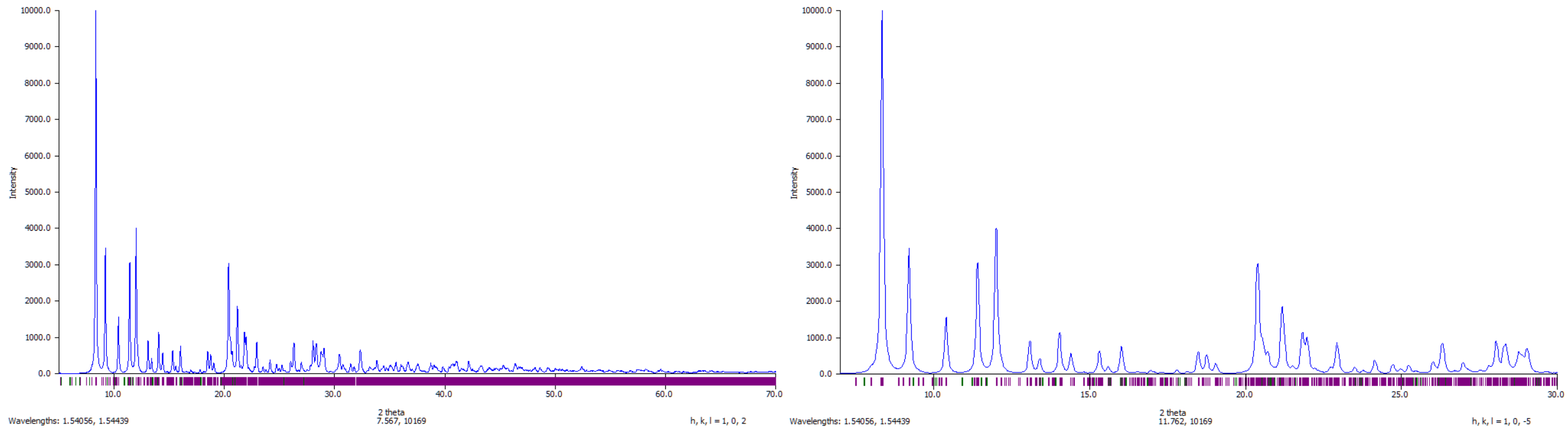

Figure S13. Calculated powder X-ray diffraction pattern of **1-Ce** derived from crystallographic model. (a) 5–70°, (b) 7–30°. *x*-axis 2θ (°), *y*-axis intensity (a.u.).

(a) **(b)**

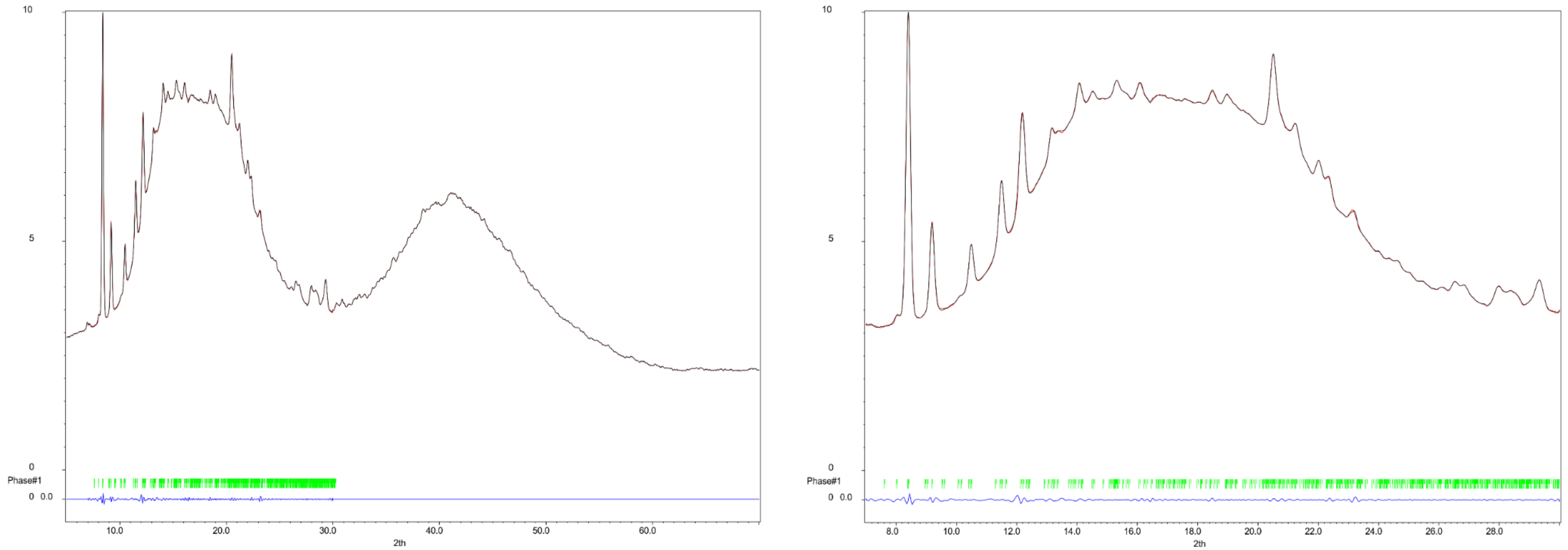

Figure S14. (a) 5–70° full range PXRD pattern of **1-Ce** , (b) Selected range 7–30° for Le Bail method profile fitting of **1-Ce**; experimental data (black), calculated fit from crystallographic parameters (red) and the difference (blue). Le Bail method profile fittingRp = 0.19; wRp = 0.31. Single crystal unit cell values were used for the initial starting unit cell. *x*-axis 2θ (°), *y*-axis intensity (a.u.).

(a) (b)

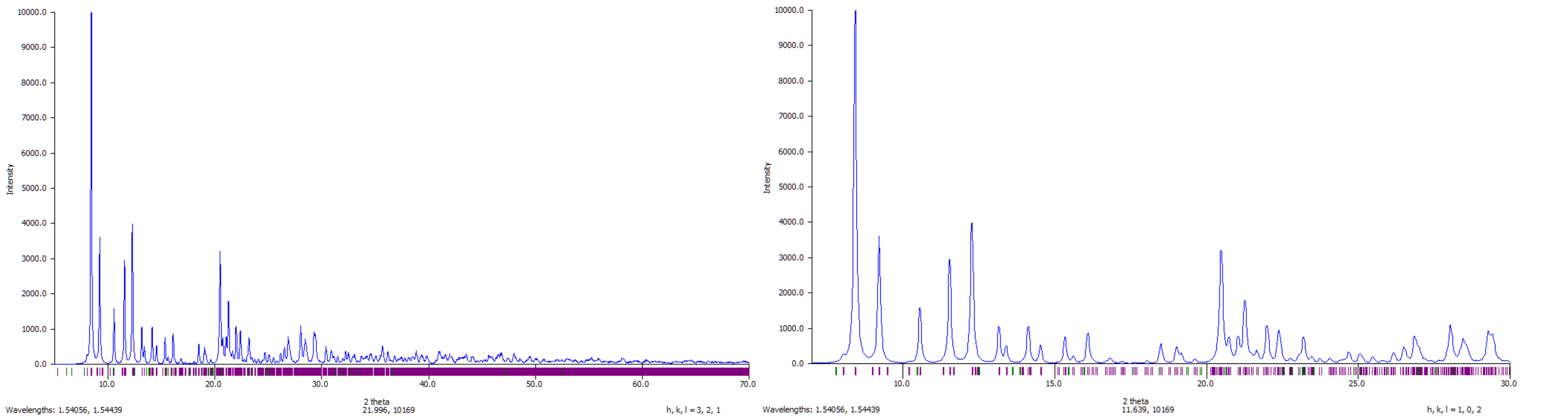

Figure S15. Calculated powder X-ray diffraction pattern of **1-Pr** derived from crystallographic model. (a) 5–70°, (b) 7–30°. *x*-axis 2θ (°), *y*-axis intensity (a.u.).

(a)

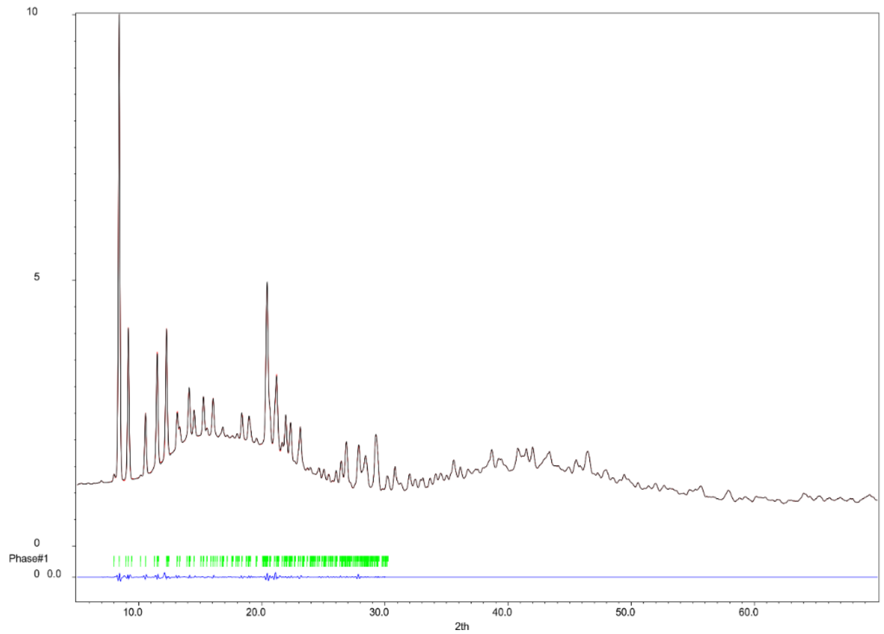

(b)

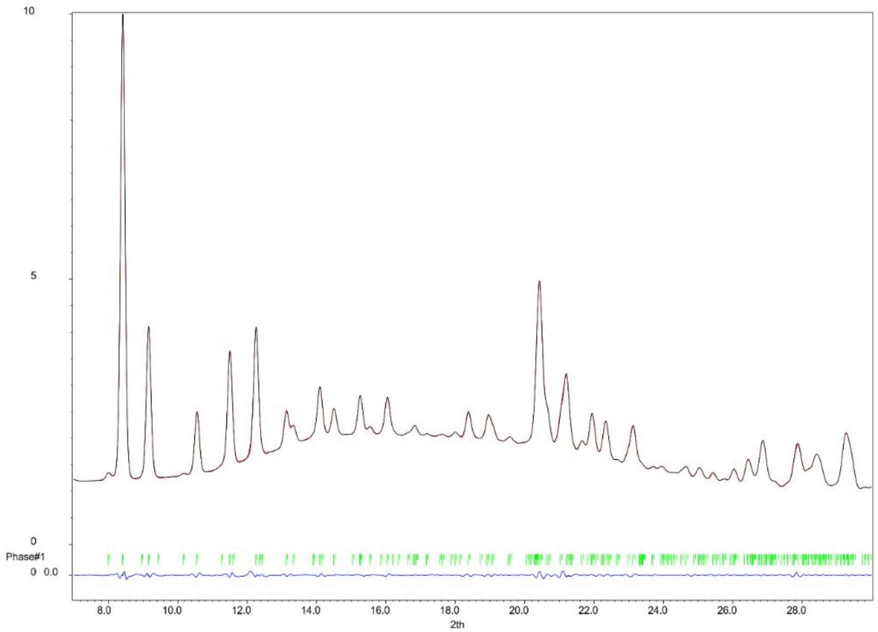

Figure S16. (a) 5–70° full range PXRD pattern of **1-Pr**, (b) Selected range 7–30° for Le Bail method profile fitting of **1-Pr**; experimental data (black), calculated fit from crystallographic parameters (red) and the difference (blue). Le Bail method profile fitting Rp = 0.47; wRp = 0.69. Single crystal unit cell values were used for the initial starting unit cell. *x*-axis 2θ (°), *y*-axis intensity (a.u.).

(a) **(b)**

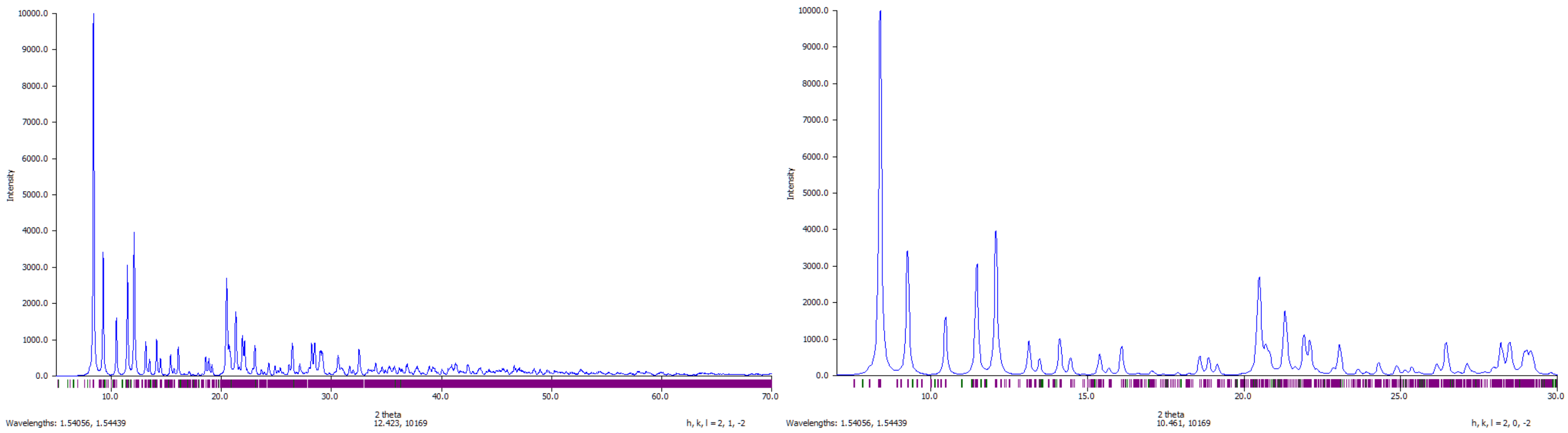

Figure S17. Calculated powder X-ray diffraction pattern of **1-Nd** derived from crystallographic model. (a) 5–70°, (b) 7–30 °. *x*-axis 2θ (°), *y*-axis intensity (a.u.).

(a)

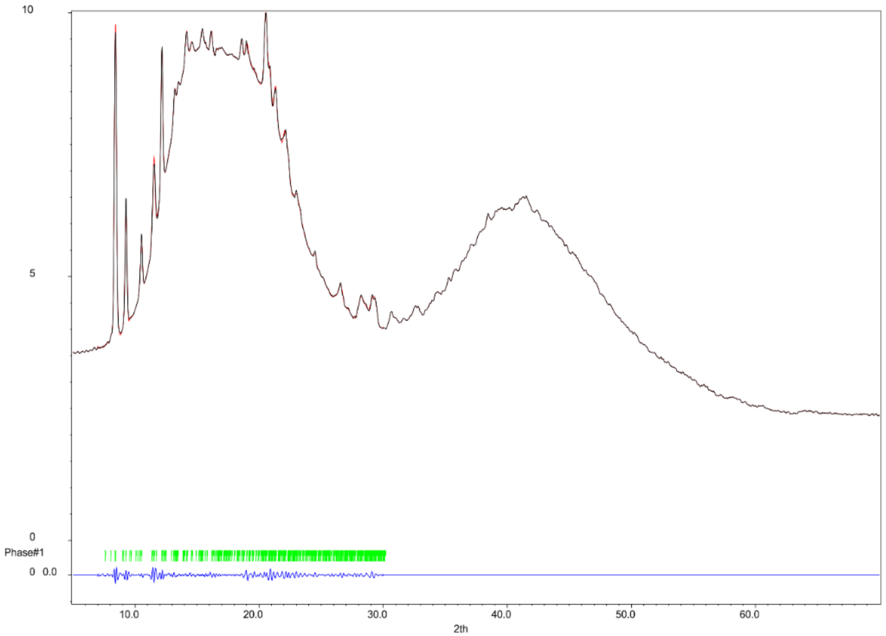

(b)

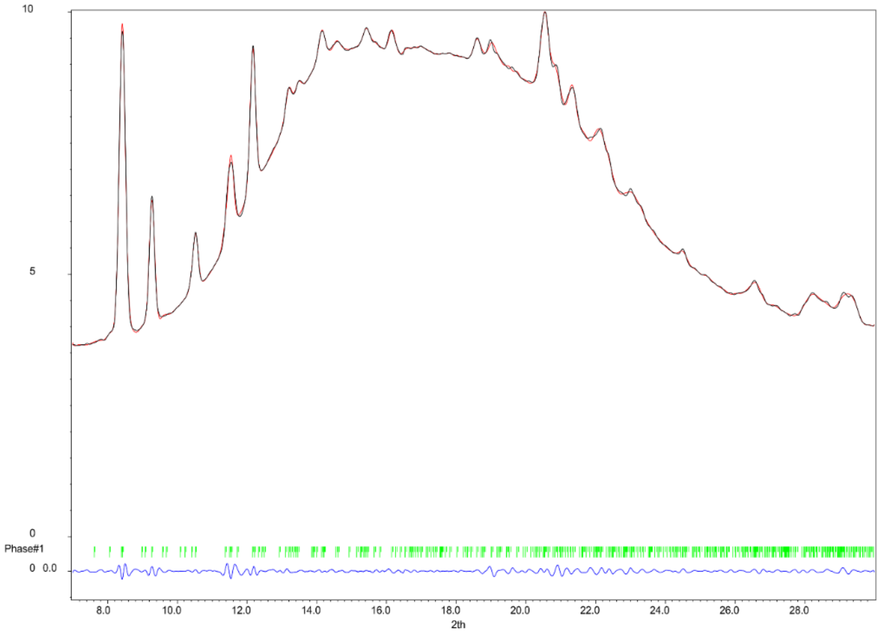

Figure S18. (a) 5–70° full range PXRD pattern of **1-Nd**, (b) Selected range 7–30° for Le Bail method profile fitting of **1-Nd**; experimental data (black), calculated fit from crystallographic parameters (red) and the difference (blue). Le Bail method profile fitting Rp = 0.32; wRp = 0.46. Single crystal unit cell values were used for the initial starting unit cell. *x*-axis 2θ (°), *y*-axis intensity (a.u.).

(a) (b)

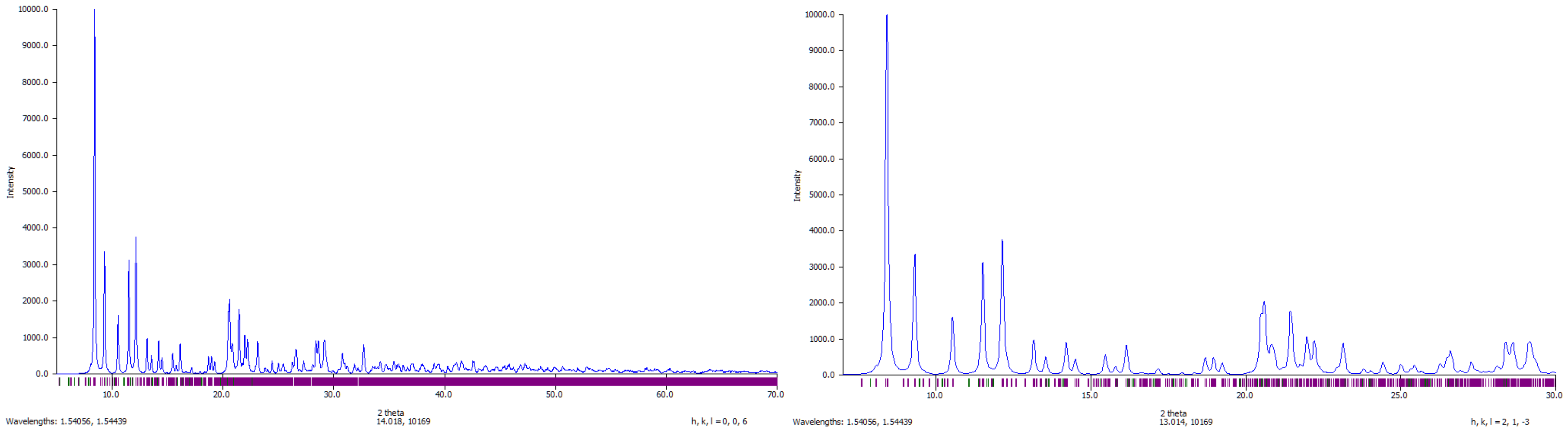

Figure S19. Calculated powder X-ray diffraction pattern of **1-Sm** derived from crystallographic model. (a) 5–70°, (b) 7–30 °. *x*-axis 2θ (°), *y*-axis intensity (a.u.).

(a)

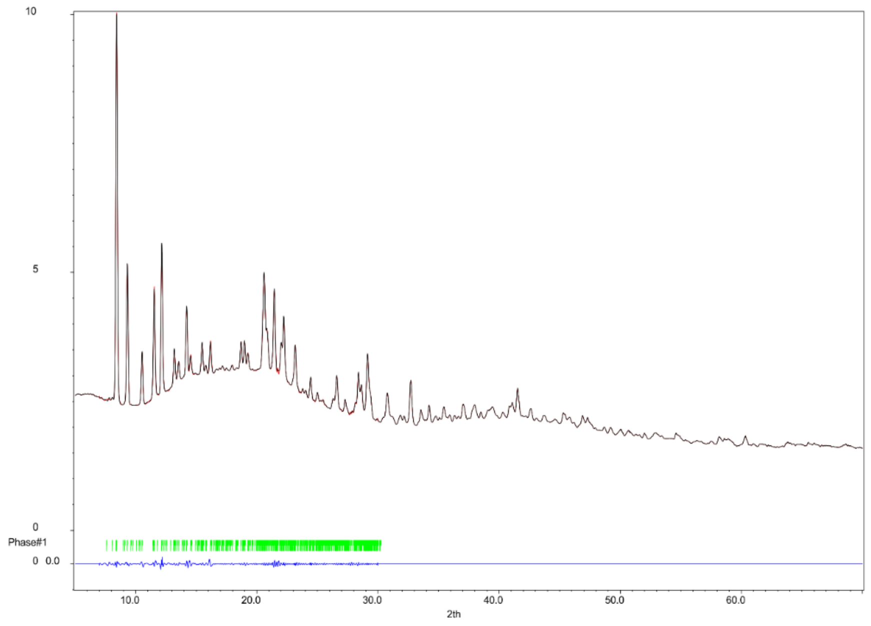

(b)

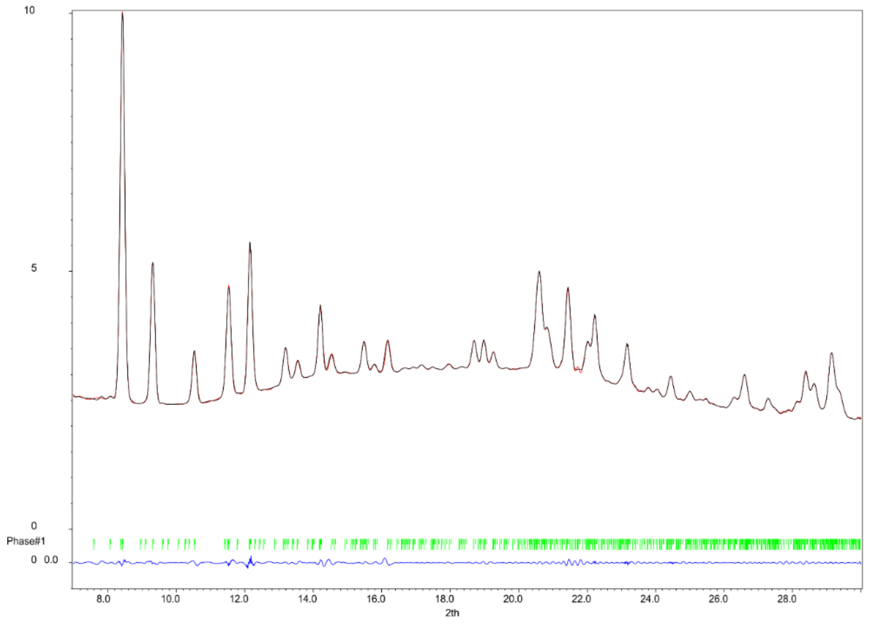

Figure S20. (a) 5–70° full range PXRD pattern of **1-Sm**, (b) Selected range 7–30° for Le Bail method profile fitting of **1-Sm**; experimental data (black), calculated fit from crystallographic parameters (red) and the difference (blue). Le Bail method profile fitting Rp = 0.35; wRp = 0.55. Single crystal unit cell values were used for the initial starting unit cell. *x*-axis 2θ (°), *y*-axis intensity (a.u.).

Table S4. Unit cell values obtained from Le Bail refinement results.

**Complex**	A	B	C	α	β	γ
**1-La**	17.2572(5)	13.4714(4)	19.8460(5)	90	103.6463(16)	90
**1-Ce**	17.3645(7)	13.4694(6)	39.3432(18)	90	101.745(3)	90
**1-Pr**	17.2367(3)	13.4751(2)	19.8506(3)	90	103.5509(9)	90
**1-Nd**	17.3337(12)	13.4142(11)	38.946(3)	90	101.449(4)	90
**1-Sm**	17.3568(3)	13.4060(2)	38.6497(6)	90	100.9714(11)	90

5. Solution NMR Spectroscopy: 1-Ln

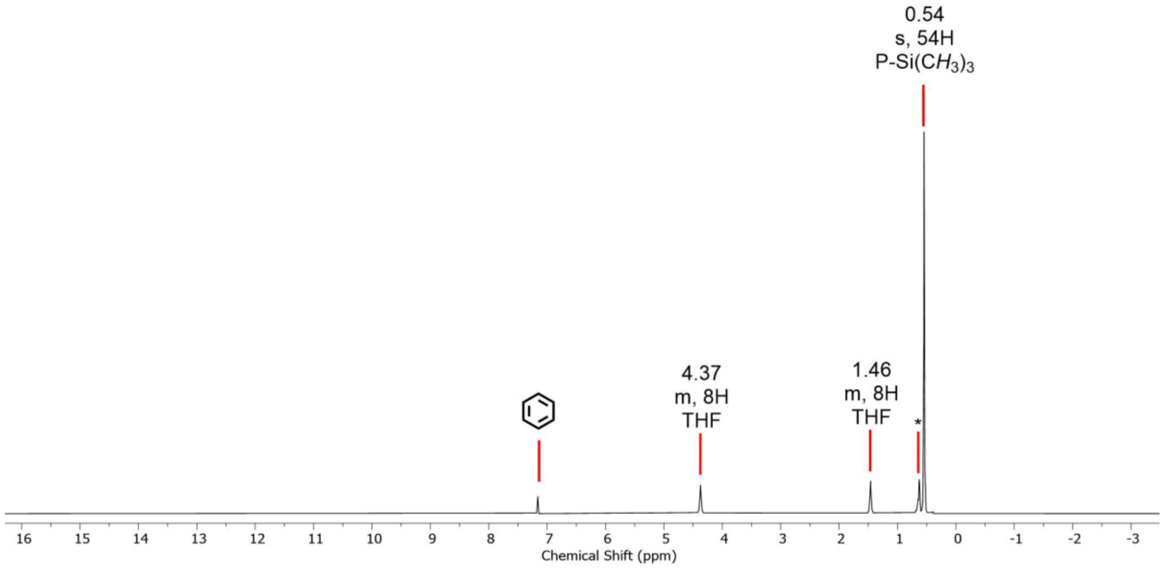

Figure S21. ^{1}H NMR spectrum (400 MHz) of **1-La** in C_6D_6. * denotes silicon grease impurity.

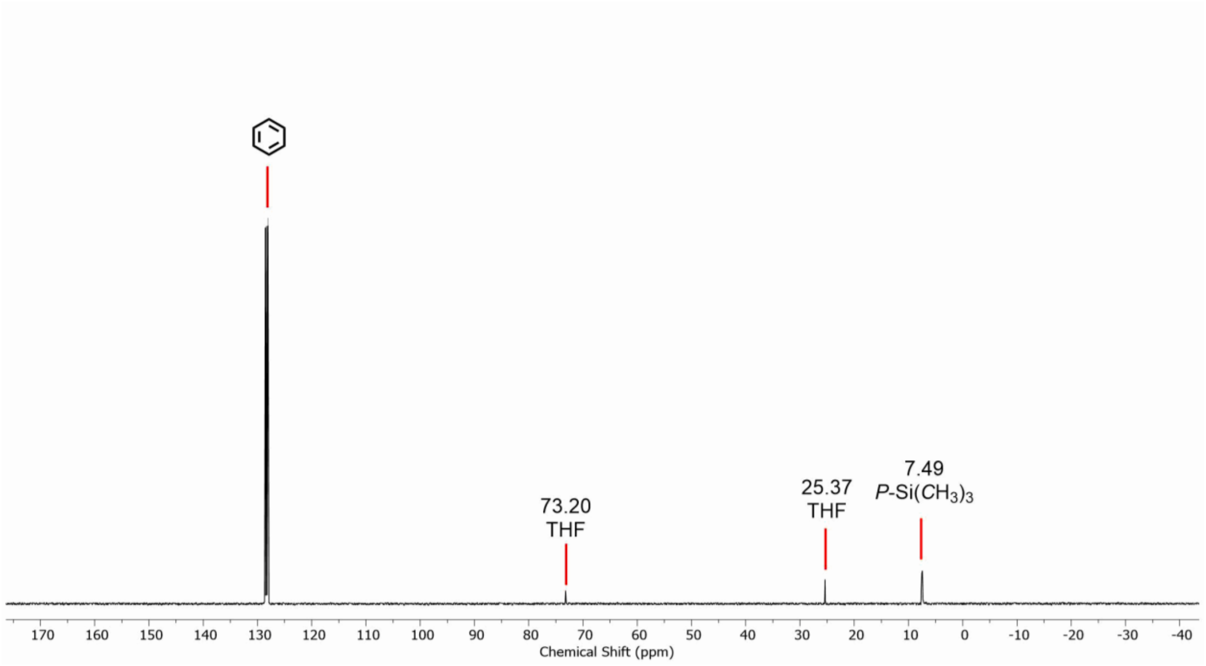

Figure S22. $^{13}C\{^{1}H\}$ NMR spectrum (101 MHz) of **1-La** in C_6D_6.

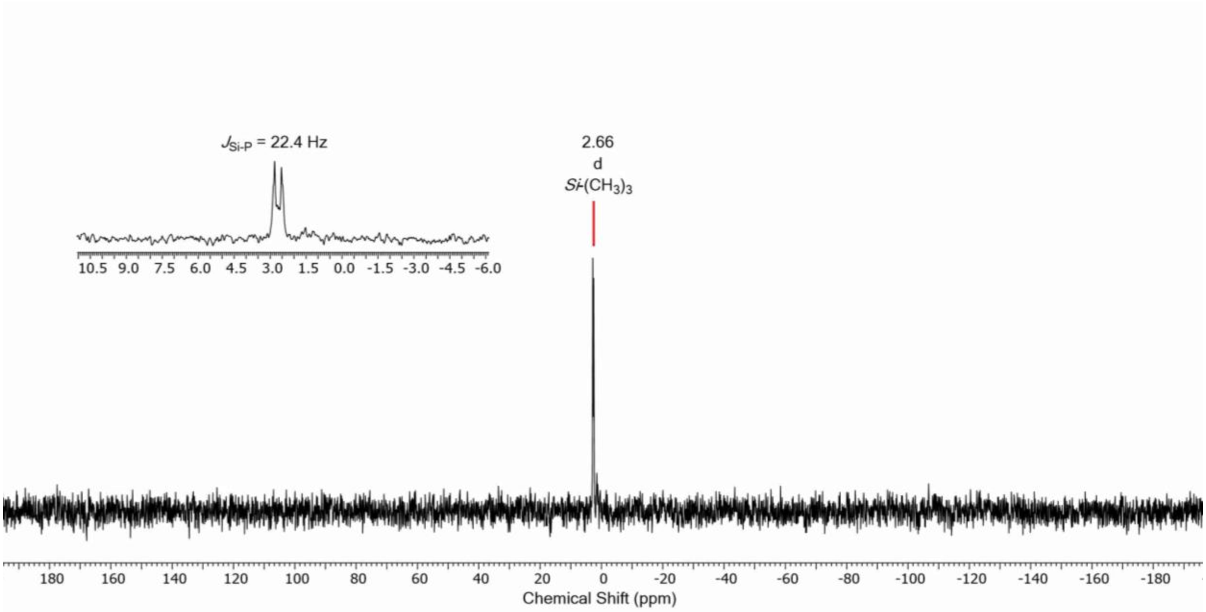

Figure S23. ^{29}Si DEPT90 NMR spectrum (79 MHz) of **1-La** in C_6D_6.

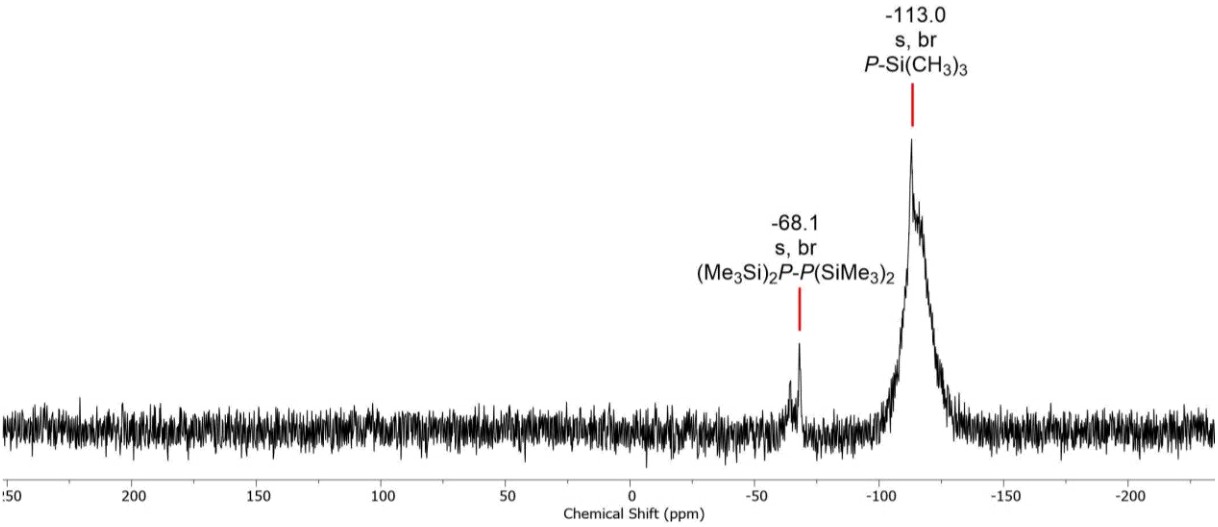

Figure S24. ^{31}P{^{1}H} NMR spectrum (162 MHz) of **1-La** in C_6D_6, zoomed in the region +250 to –230 ppm.

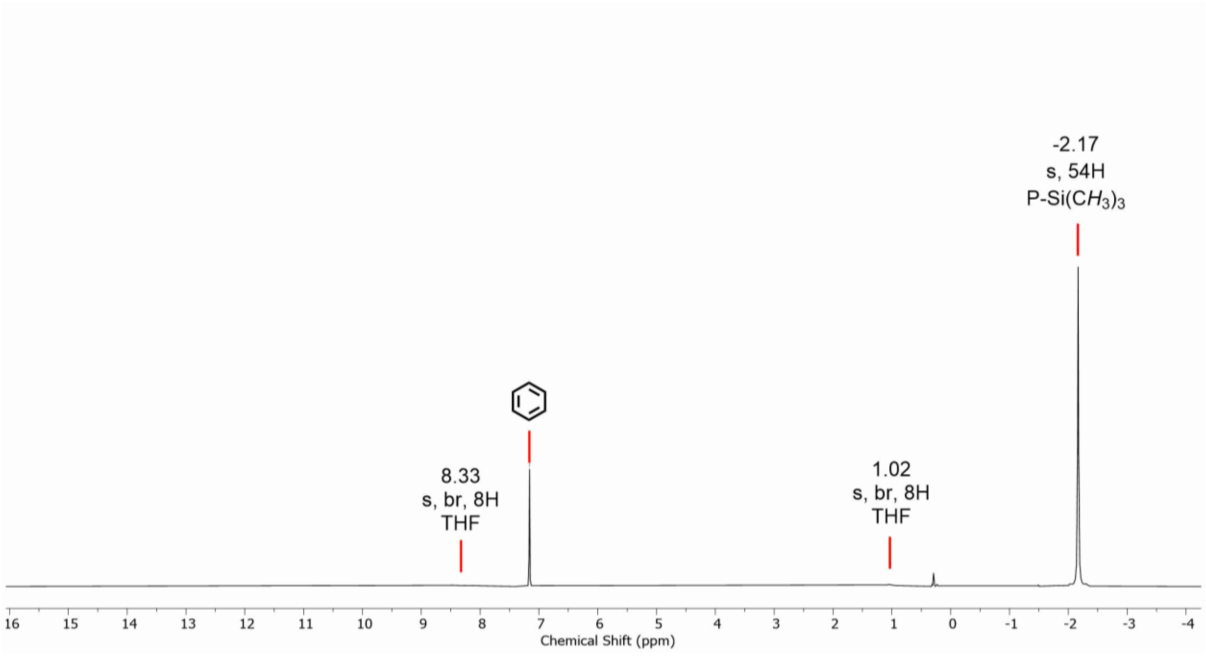

Figure S25. ^{1}H NMR spectrum (400 MHz) of **1-Ce** in C_6D_6.

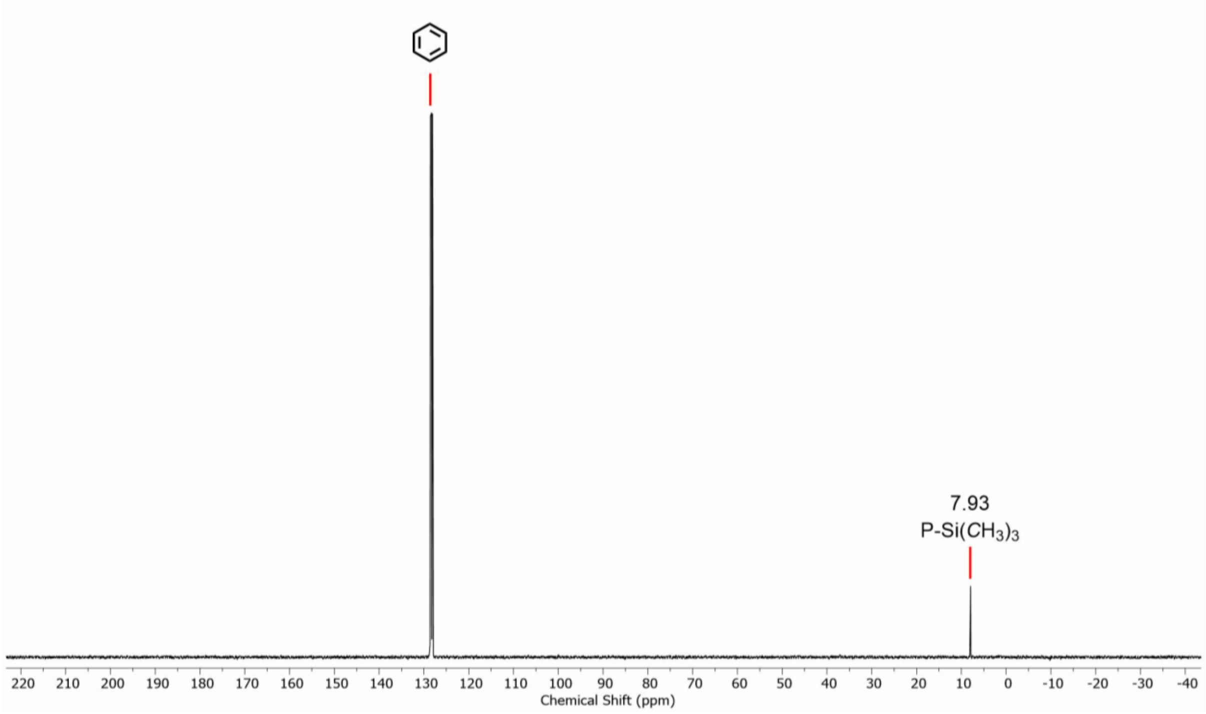

Figure S26. $^{13}C\{^{1}H\}$ NMR spectrum (101 MHz) of **1-Ce** in C_6D_6**.**

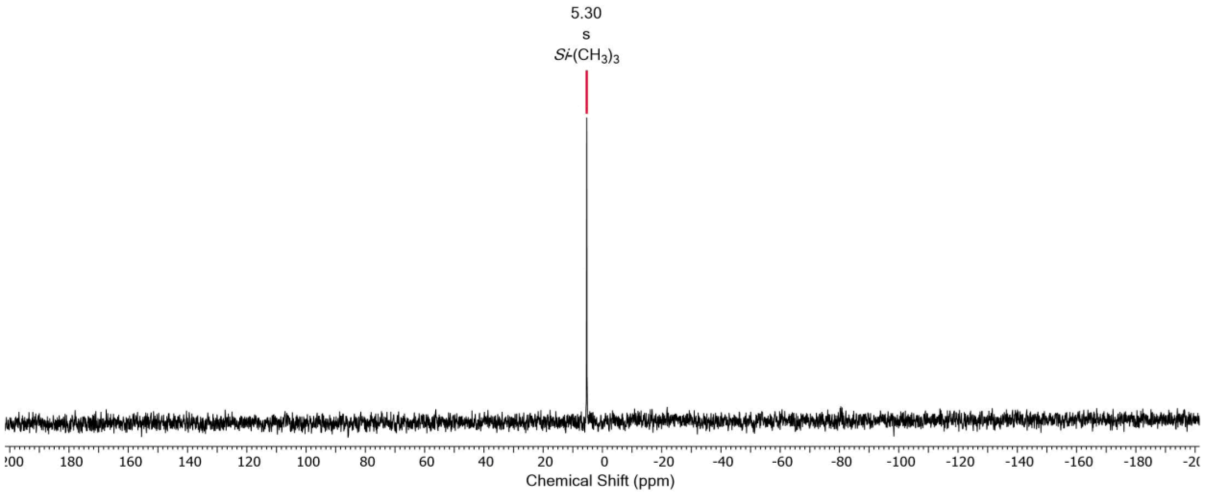

Figure S27. ^{29}Si DEPT90 NMR spectrum (79 MHz) of **1-Ce** in C_6D_6.

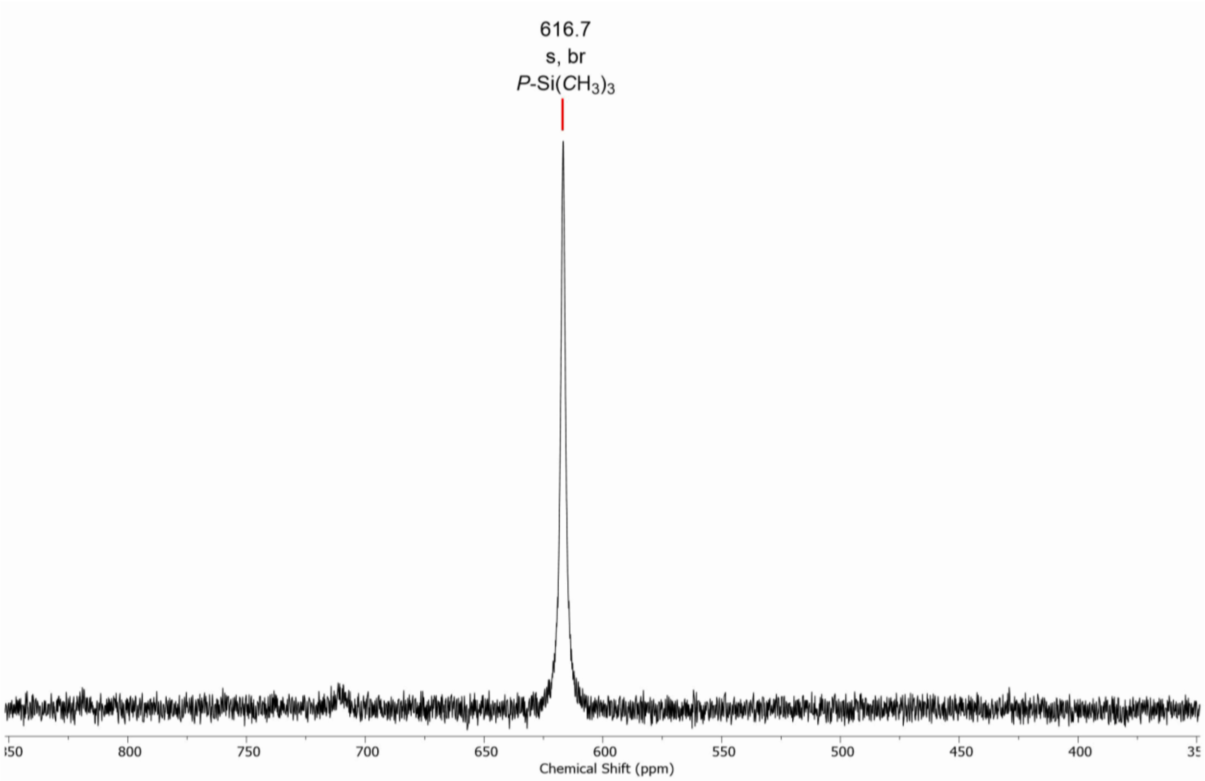

Figure S28. ^{31}P{^{1}H} NMR spectrum (162 MHz) of **1-Ce** in C_6D_6, zoomed in the region +950 to +350 ppm.

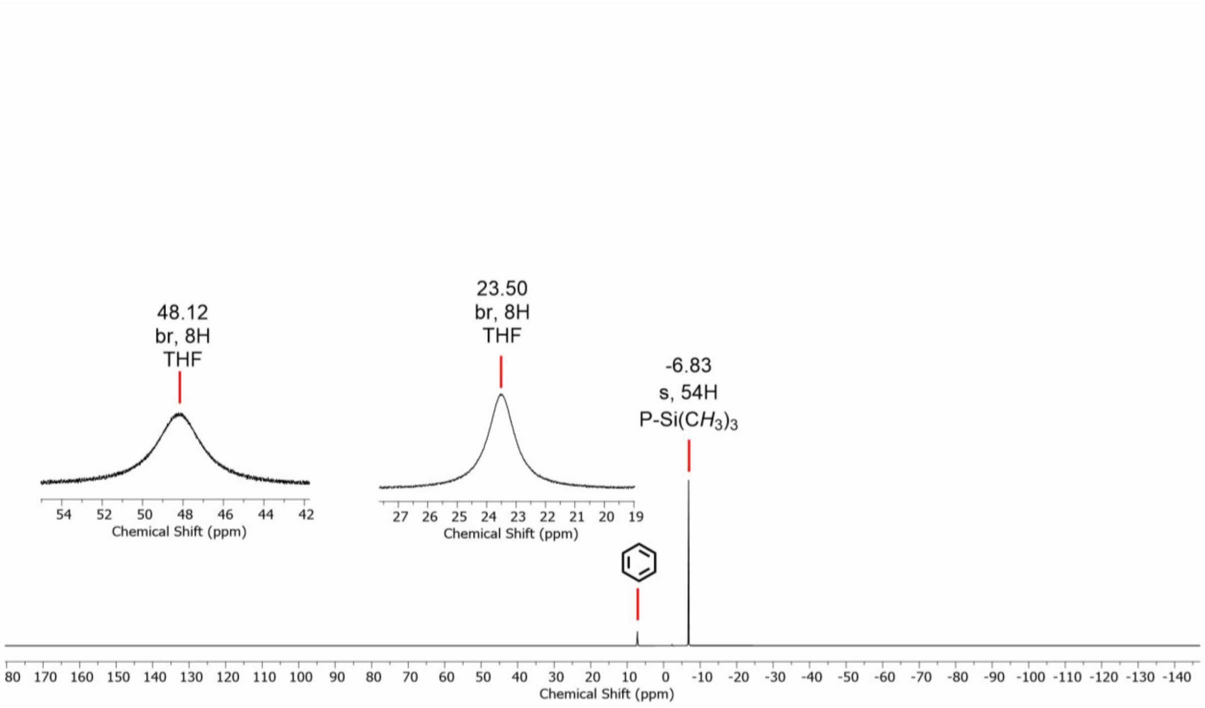

Figure S29. ^{1}H NMR spectrum (400 MHz) of **1-Pr** in C_6D_6.

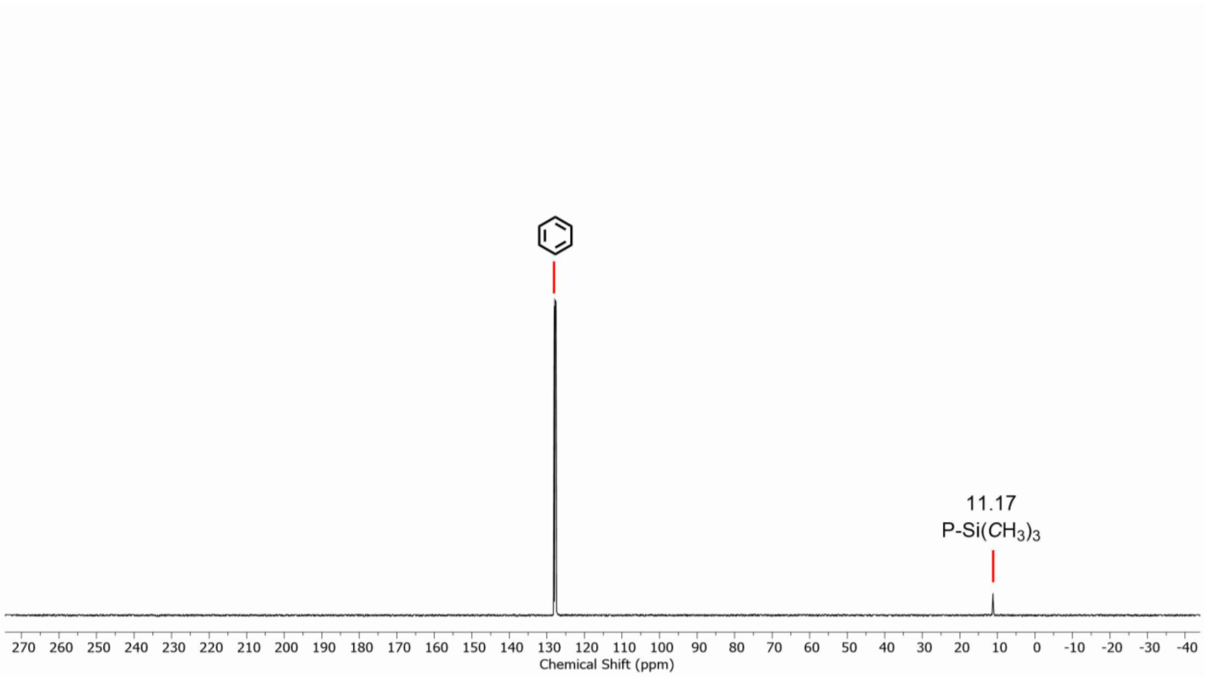

Figure S30. $^{13}C\{^{1}H\}$ NMR spectrum (101 MHz) of **1-Pr** in C_6D_6.

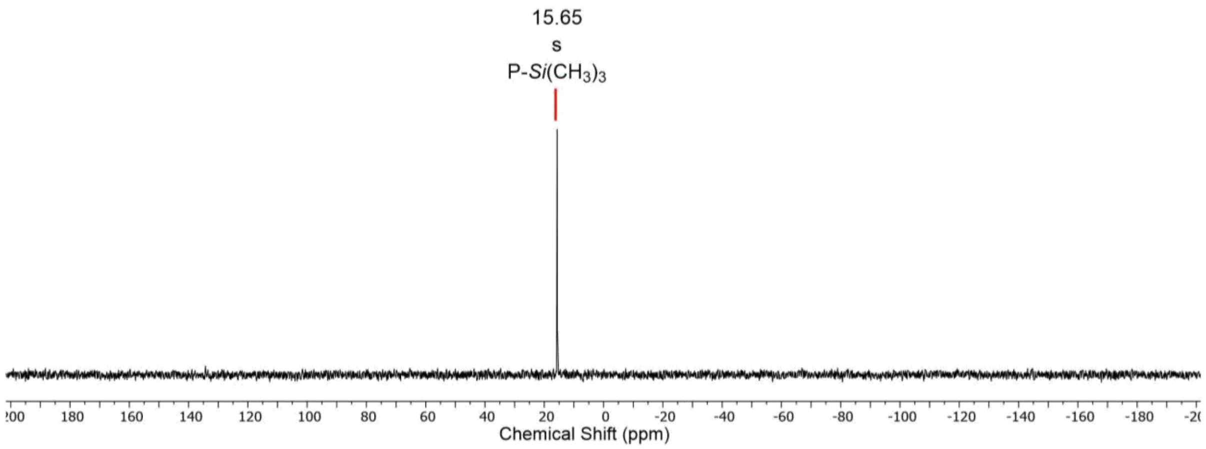

Figure S31. ^{29}Si DEPT90 NMR spectrum (79 MHz) of **1-Pr** in C_6D_6.

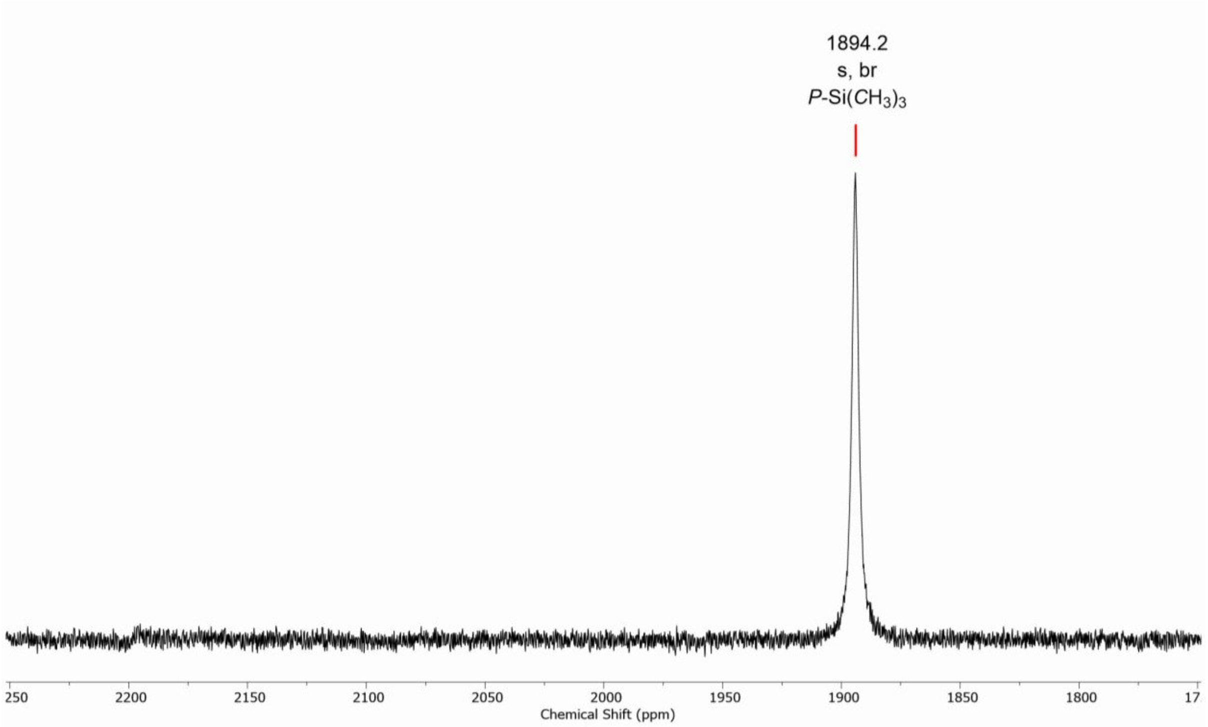

Figure S32. ^{31}P{^{1}H} NMR spectrum (162 MHz) of **1-Pr** in C_6D_6, , zoomed in the region +2250 to +1750 ppm.

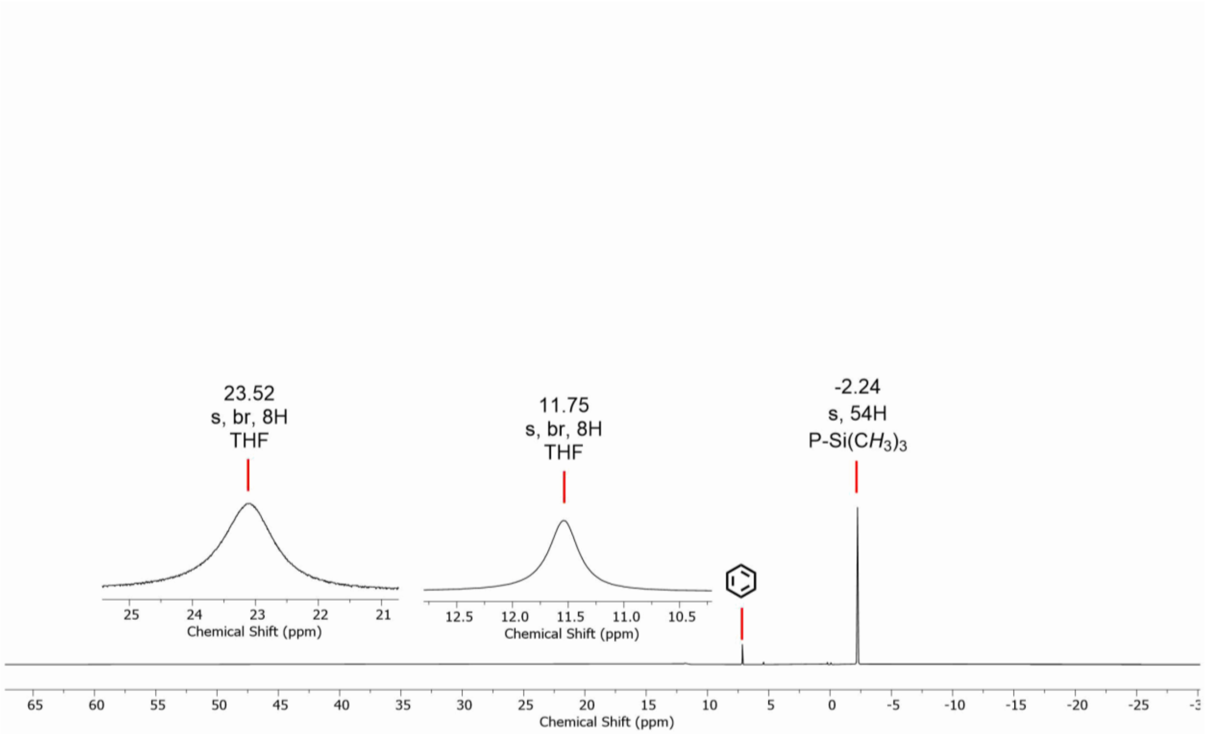

Figure S33. ^{1}H NMR spectrum (400 MHz) of **1-Nd** in C_6D_6.

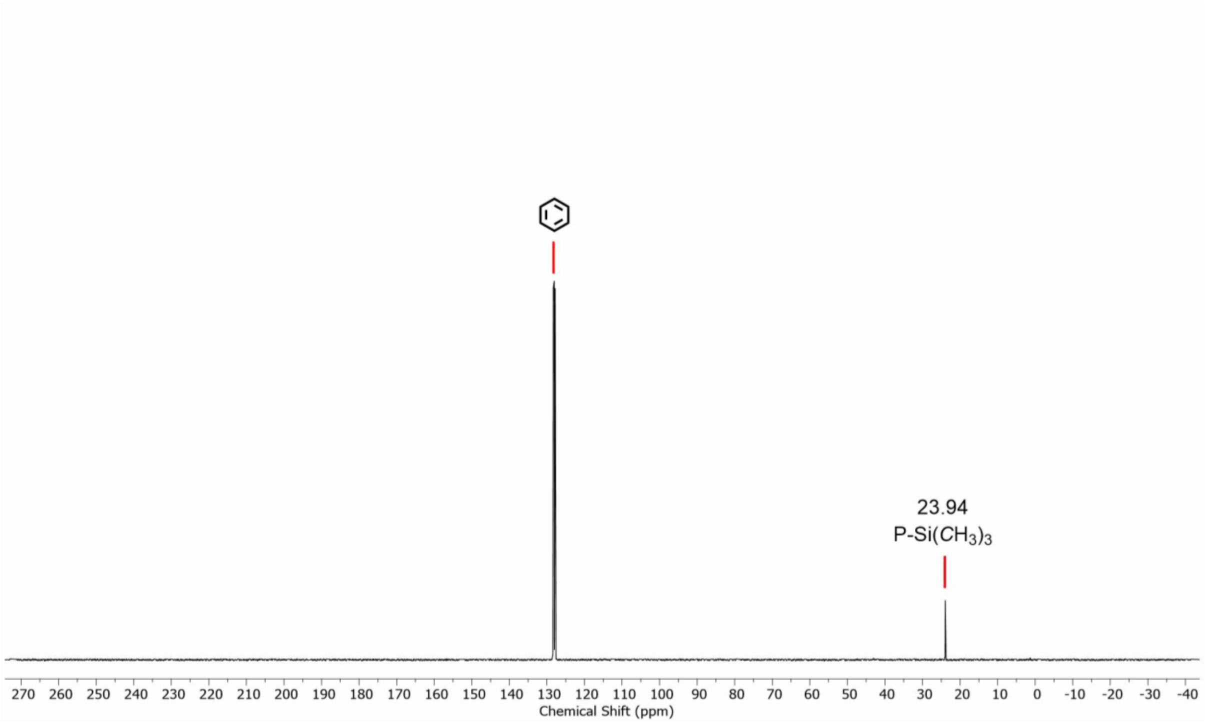

Figure S34. ^{13}C{^{1}H} NMR spectrum (101 MHz) of **1-Nd** in C_6D_6.

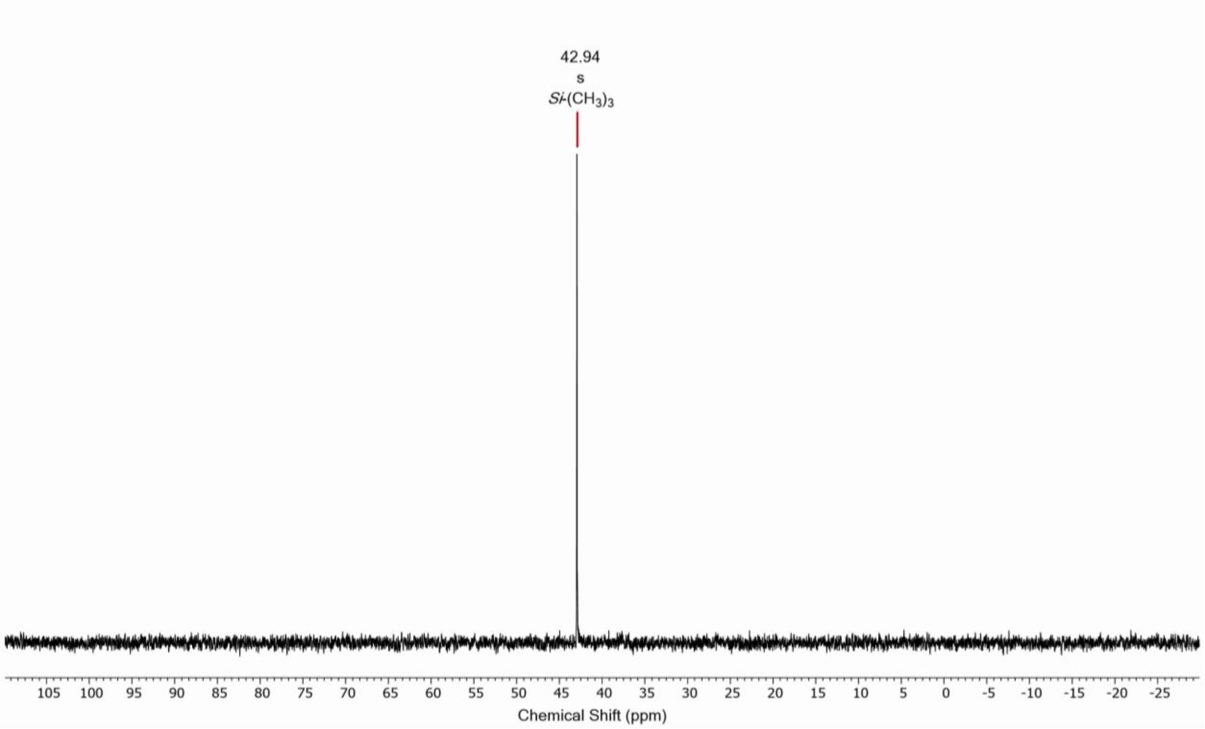

Figure S35. ^{29}Si DEPT90 NMR spectrum (79 MHz) of **1-Nd** in C_6D_6.

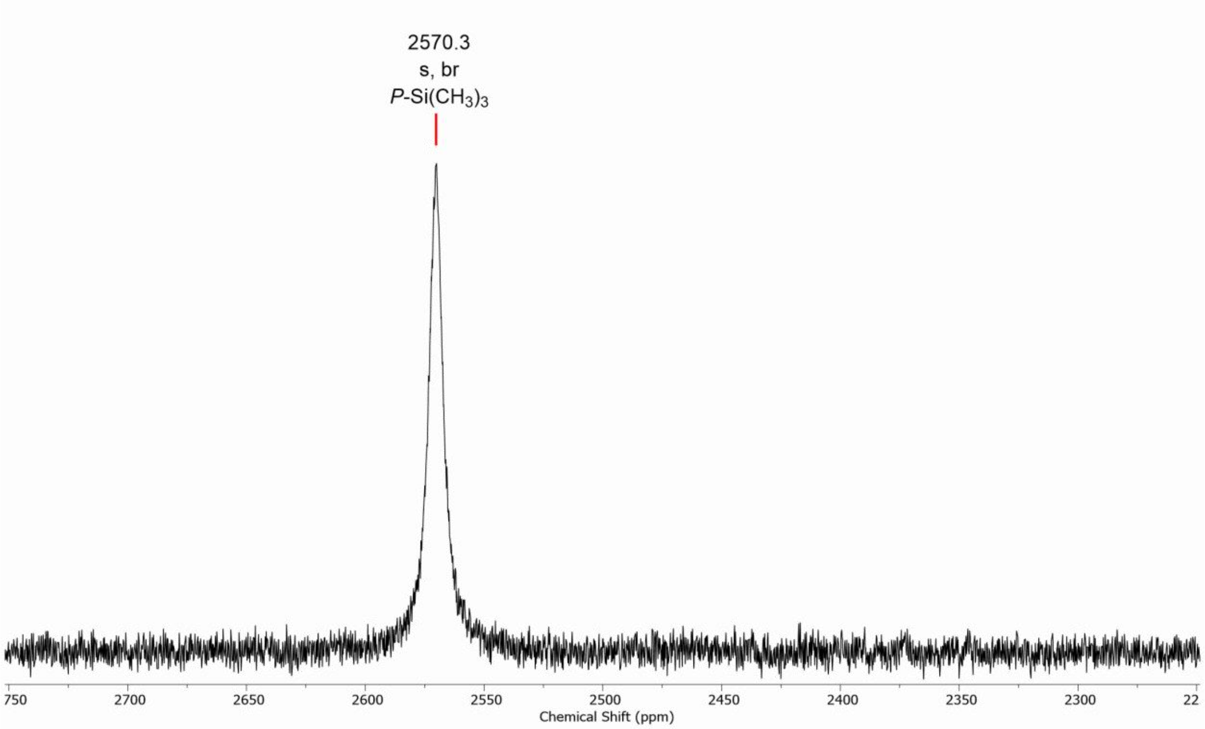

Figure S36. ^{31}P{^{1}H} NMR spectrum (162 MHz) of **1-Nd** in C_6D_6, zoomed in the region +2750 to +2250 ppm.

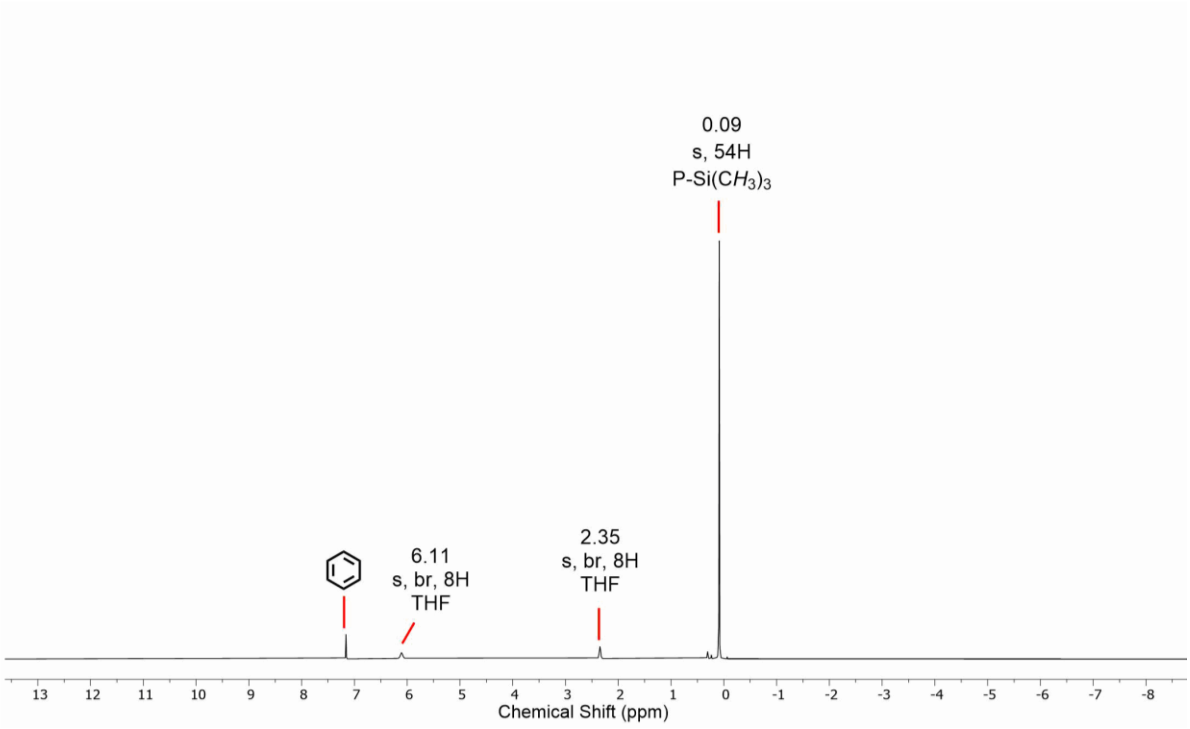

Figure S37. ^{1}H NMR spectrum (400 MHz) of **1-Sm** in C_6D_6.

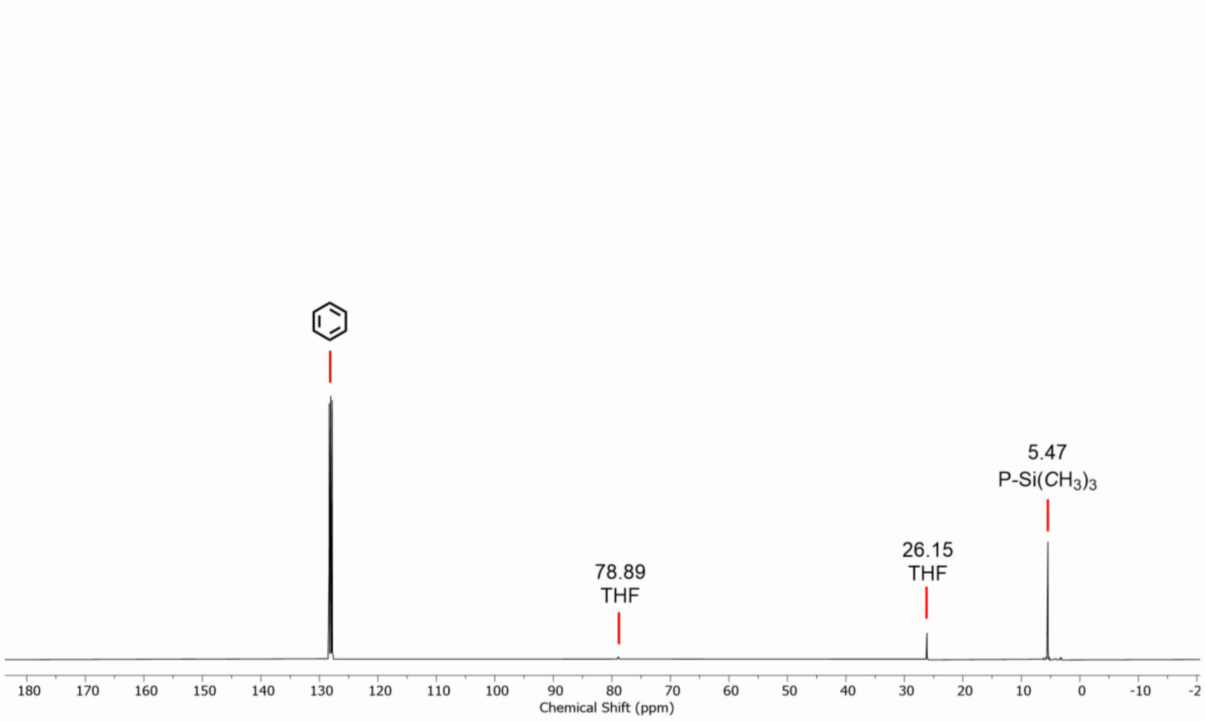

Figure S38. $^{13}C\{^{1}H\}$ NMR spectrum (101 MHz) of **1-Sm** in C_6D_6.

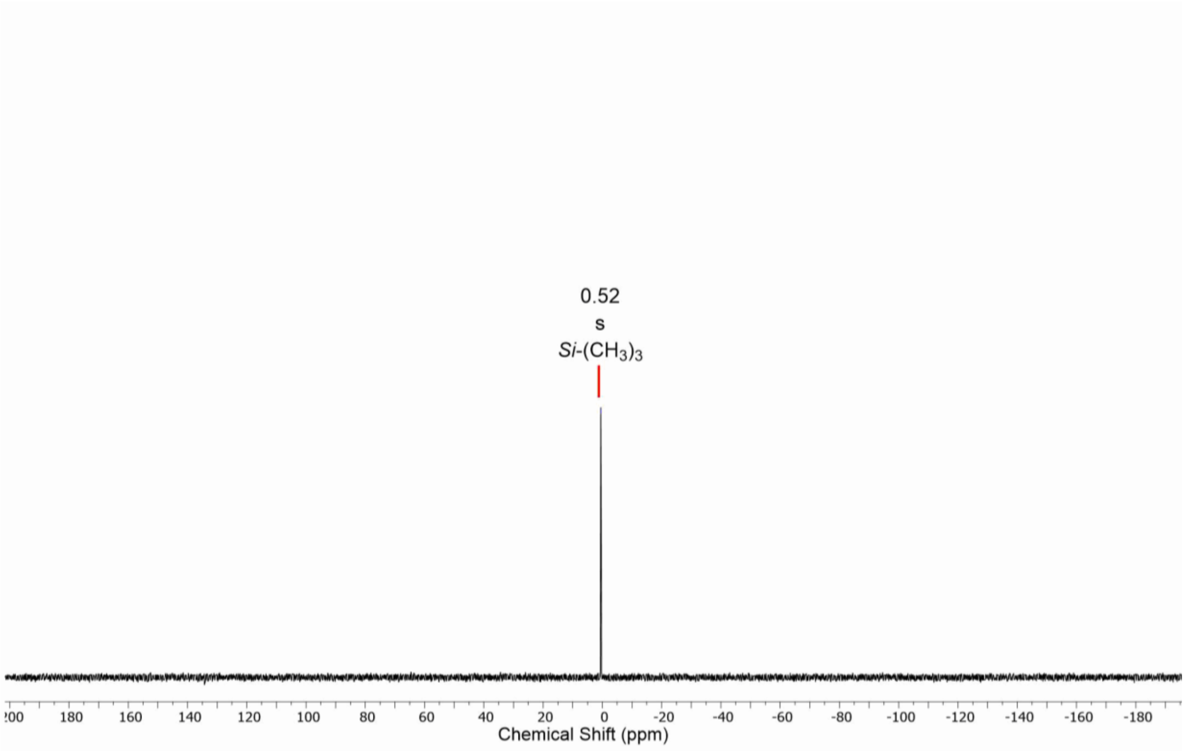

Figure S39. ^{29}Si DEPT90 NMR spectrum (79 MHz) of **1-Sm** in C_6D_6.

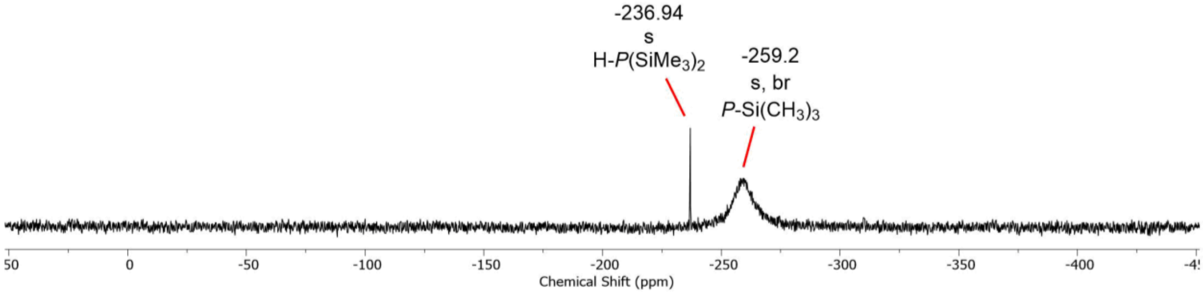

Figure S40. ^{31}P{^{1}H} NMR spectrum (162 MHz) of **1-Sm** in C_6D_6, zoomed in the region +50 to –450 ppm.

Table S5. Experimental parameters for ^{1}H, $^{13}C\{^{1}H\}$ and ^{29}Si DEPT90 NMR spectra reported herein.

Parameter	Nuclei		
	^{1}H	^{13}C	^{29}Si
NS[a]	4	16	32
D1[b]	1.0	2.0	5.0
SW[c]	400	320	400
O1P[d]	0	115	0

[a] NS = number of scans.

[b] D1 = relaxation decay time in s.

[c] SW = sweep width in ppm.

[d] O1P = irradiation (carrier) frequency offset in ppm.

Table S6. Experimental parameters for the $^{31}P\{^{1}H\}$ NMR spectra reported in this study.

Parameter	Complex				
	**1-La**	**1-Ce**	**1-Pr**	**1-Nd**	**1-Sm**
NS[a]	16	16	16	16	16
D1[b]	2.0	2.0	2.0	2.0	2.0
SW[c]	500	500	500	500	500
O1P[d]	0	600	2000	2500	-200

[a] NS = number of scans.

[b] D1 = relaxation decay time in s.

[c] SW = sweep width in ppm.

[d] O1P = irradiation (carrier) frequency offset in ppm.

6. Solid-state NMR Spectroscopy: 1-Ln

Table S7. Experimental parameters used for ^{31}P MAS NMR spectroscopy of **1-Ln**. "Expt" defines the pulse sequence used.

Parameter	Complex				
	**1-La**	**1-Ce**	**1-Pr**	**1-Nd**	**1-Sm**
Expt	DE[a]-echo	DE	WCPMG-MAS*	WCPMG-MAS*	DE-echo
NS[b]	128	128	131072	8192	4096
D1[c]	10.0	3.0	0.01	0.005	0.1
SW[d]	1111	400	1538	1538	2000
MAS[e]	7	8	20	20	7
O1P[f]	−140	600	1800	2500	−250
N[g]			20	20	
Echoes[h]			11	11	

[a] DE = direct excitation.

[b] NS = number of scans.

[c] D1 = relaxation decay time in s.

[d] SW = spectral width in kHz.

[e] MAS = magic angle spinning frequency in kHz.

[f] O1P = irradiation (carrier) frequency offset in ppm.

[g] N = WURST pulse N-parameter.

[h] Echoes = Number of rotor-synchronized CPMG echoes acquired per experimental scan.

*WCPMG-MAS NMR spectra are presented as spikelets and are magnitude phase-corrected. Isotropic shifts for lineshape fitting are taken from summed 1D projections from sheared 2D pjMATPASS experiments, which were recorded with 2000 scans for **1-Pr** and 3600 scans for **1-Nd** for each of 32 rotor synchronized t_1 values, and with 2.5 μs pulses.

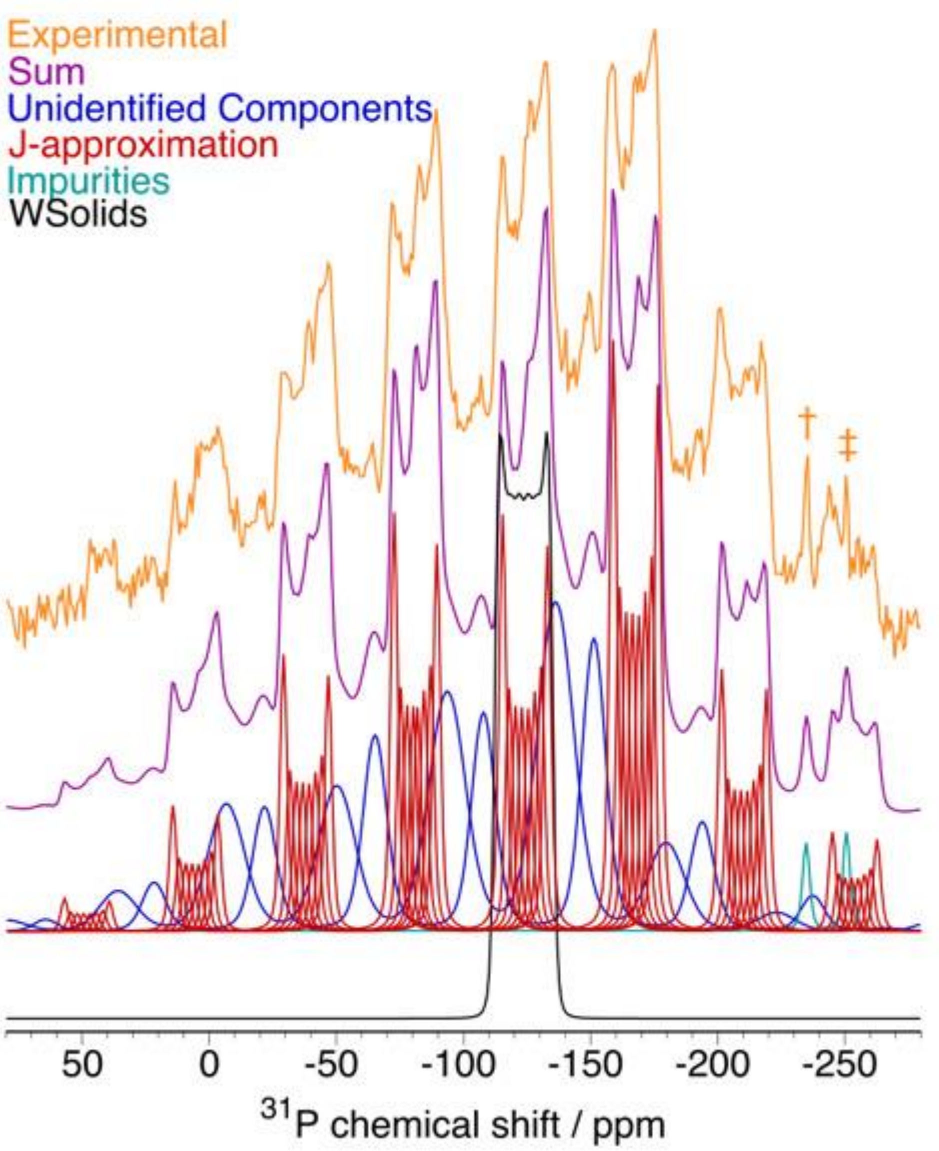

Figure S41. ^{31}P MAS NMR spectrum of **1-La** and corresponding simulated fits: WSolids[1] fit ($^{1}J_{LaP}$ 500 Hz, T_{1Q} = 2 ms) of the isotropic shifts (black) is approximated by 8 Gaussian lines of varying linewidth (red) to fit the CSA pattern. Unidentified components (dark blue) are observed along with known degradation products $HP(SiMe_3)_2$ and/or H_2PSiMe_3 and $P(SiMe_3)_3$, denoted with a dagger and double-dagger, respectively.

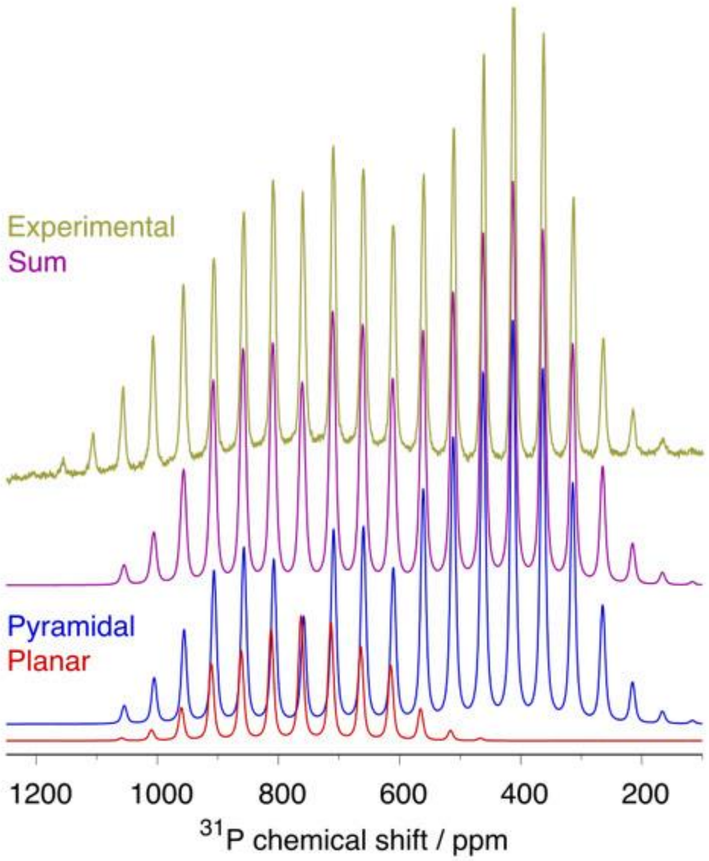

Figure S42. ^{31}P MAS NMR spectrum of **1-Ce** and corresponding simulated fit.

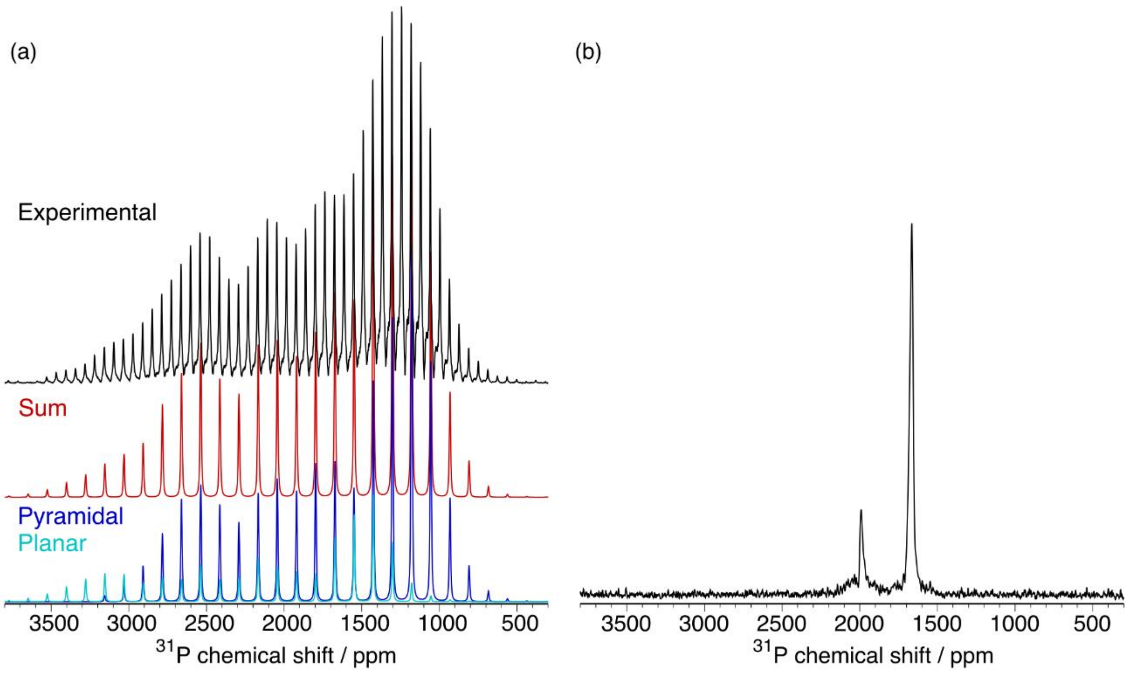

Figure S43. (a) ^{31}P WCPMG-MAS NMR spectrum with corresponding simulated fit and (b) 1D sum projection from a sheared 2D pjMATPASS NMR spectrum, providing the isotropic chemical shifts, of **1-Pr**, both recorded using a MAS frequency of 20 kHz. WURST spikelets are spaced at 10 kHz ($\nu_r/2$) intervals whereas the fit used the MAS frequency (ν_r) for accuracy, thus fitting every other spikelet.

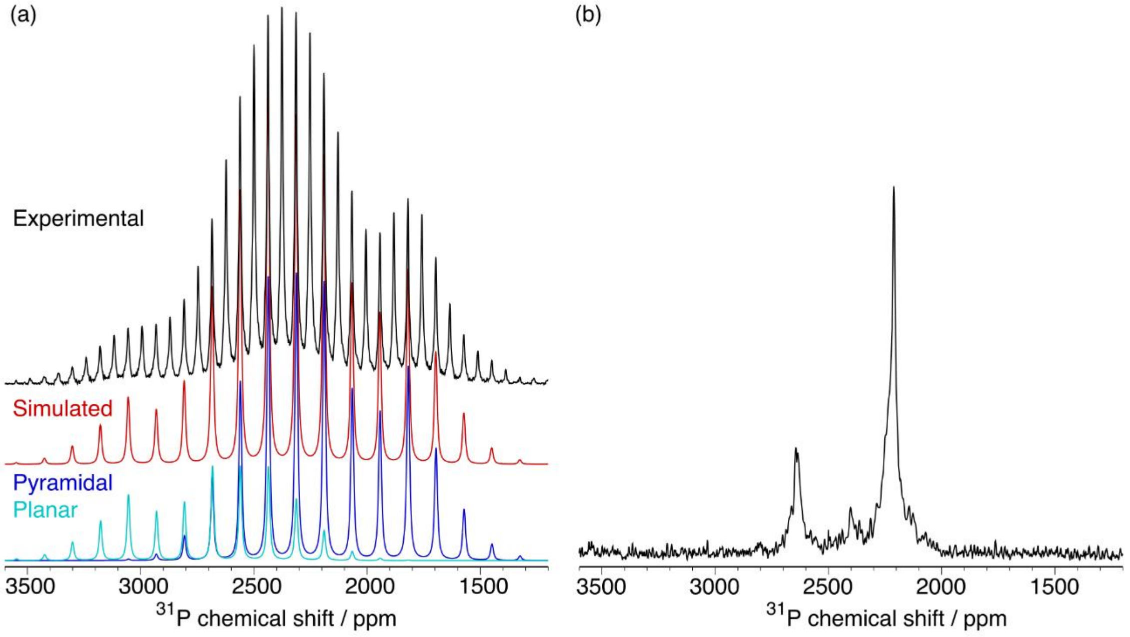

Figure S44. (a) ^{31}P WCPMG-MAS NMR spectrum with corresponding simulated fit and (b) 1D sum projection from a sheared 2D pjMATPASS NMR spectrum, providing the isotropic chemical shifts, of **1-Nd**, both recorded using a MAS frequency of 20 kHz. WURST spikelets are spaced at 10 kHz ($\nu_r/2$) intervals whereas the fit used the MAS frequency (ν_r) for accuracy, thus fitting every other spikelet.

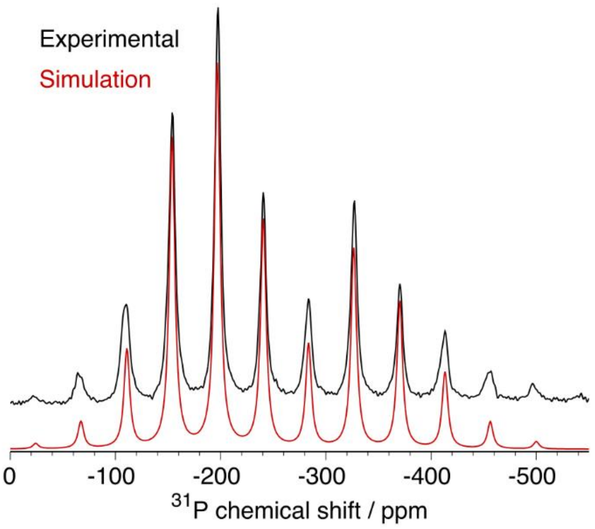

Figure S45. ^{31}P MAS NMR spectrum of **1-Sm** and corresponding simulated fit.

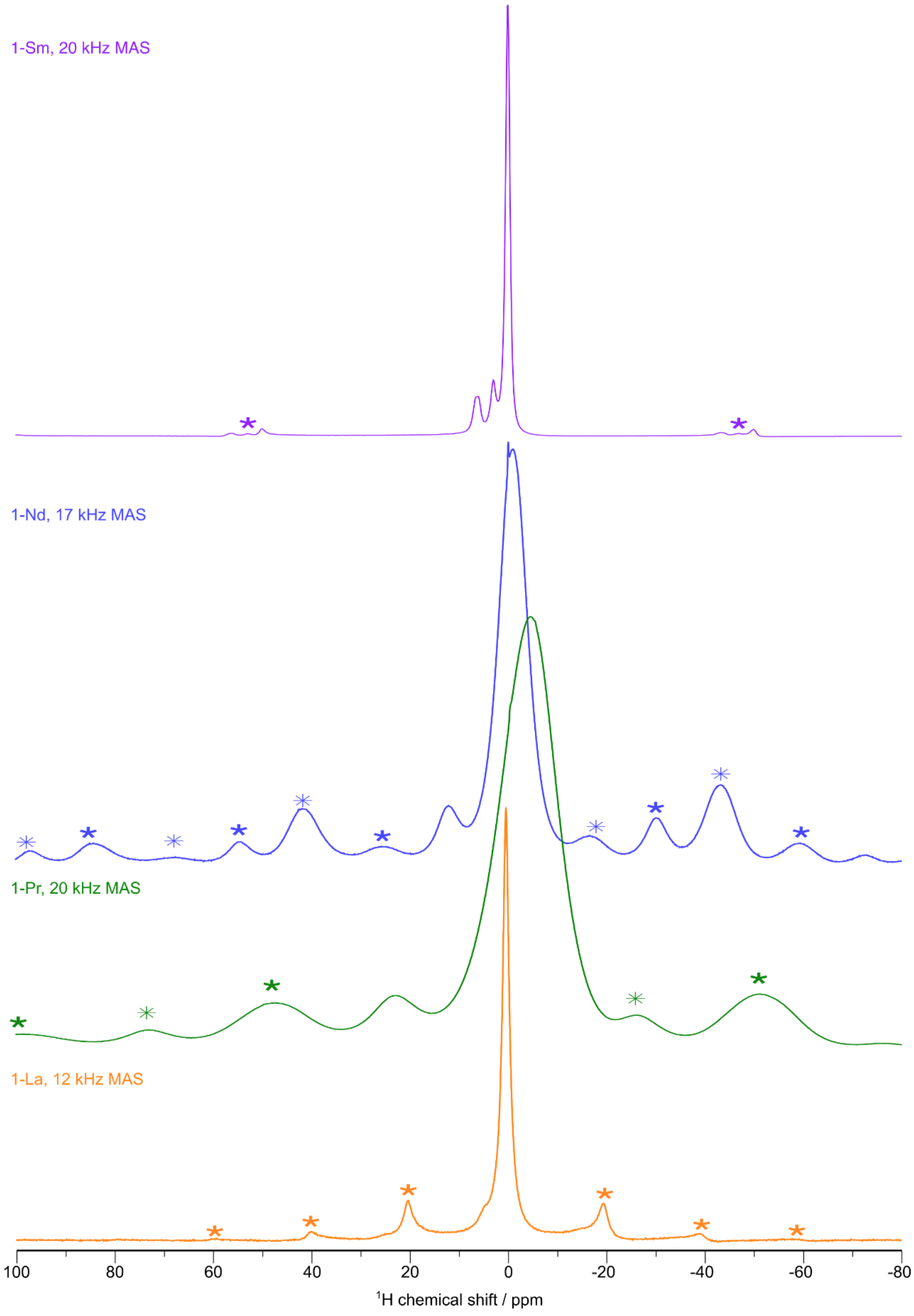

Figure S46. ^{1}H MAS NMR spectra of **1-Ln**. Asterisks (*) denote spinning side bands.

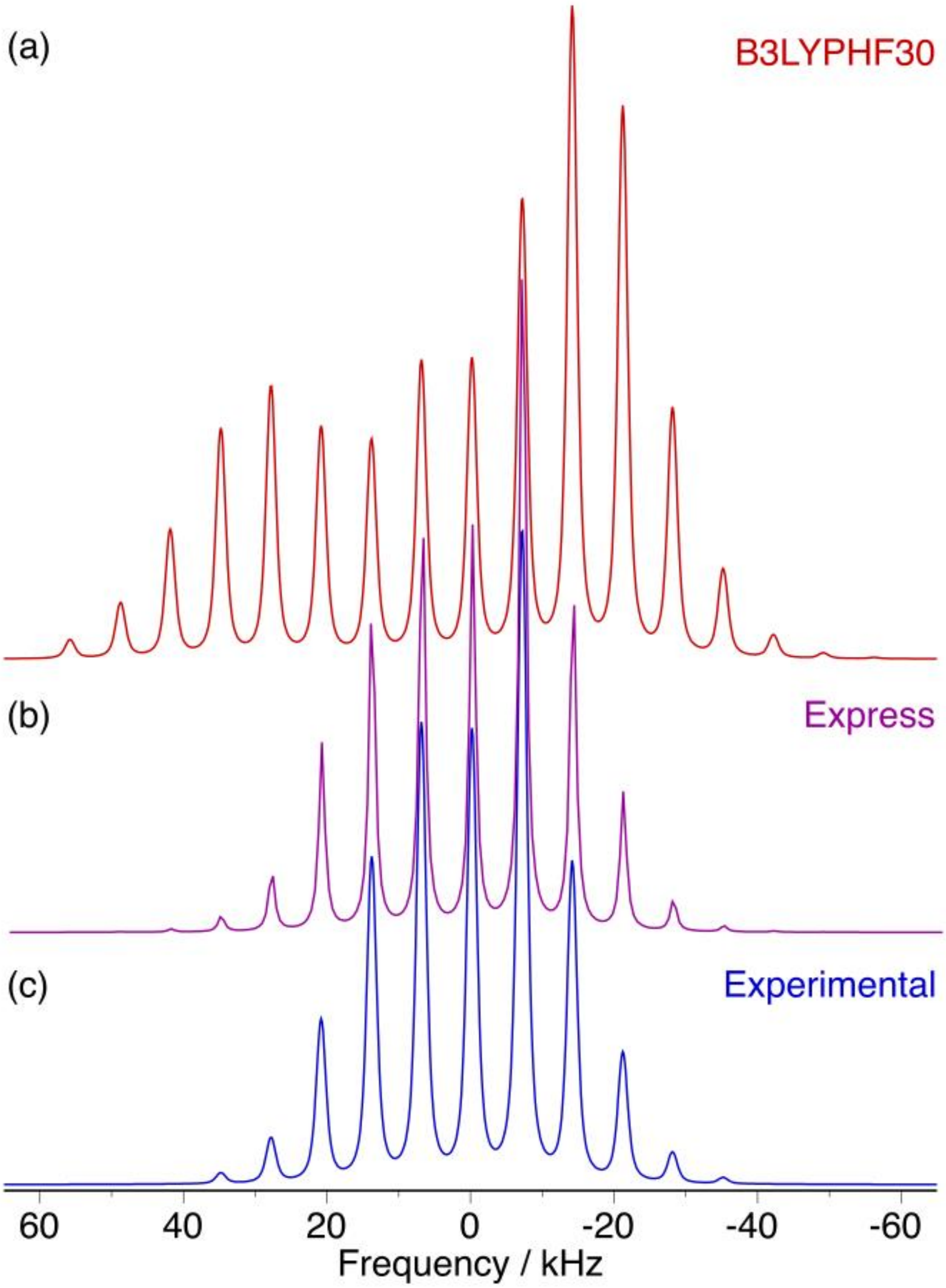

Figure S47. Simulated ^{31}P MAS NMR spectra of **1-La** using the DFT calculated CSA parameters only (a), the DFT calculated CSA parameters input to the EXPRESS[2] software and averaged assuming a libration of ±32° (α = 30°, 30°, β = 90°, 90°, γ = 32°, –32°) in the fast limit (10^{12} s^{-1}) (b), and the experimentally measured CSA parameters (c).

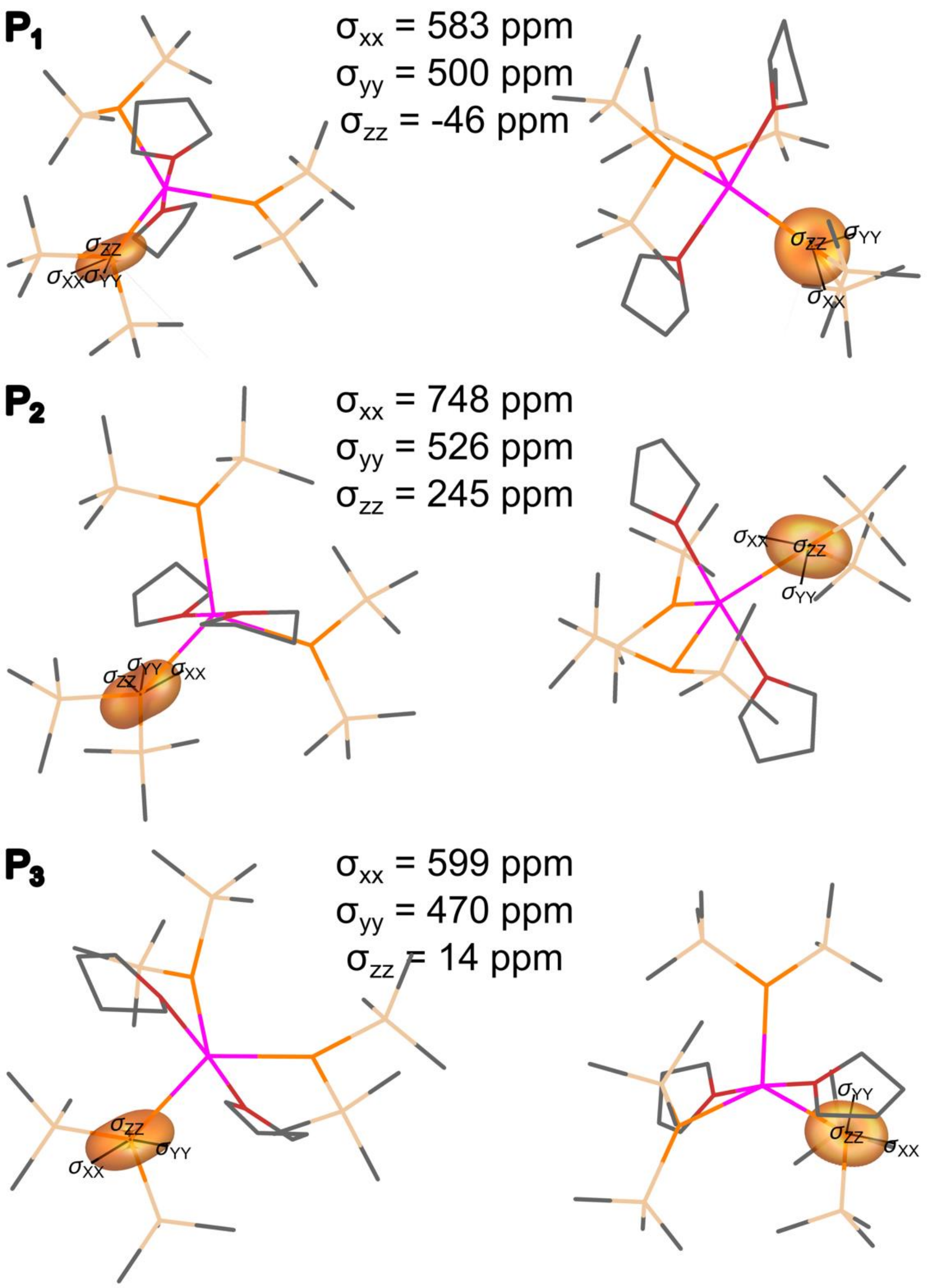

Figure S48. TensorView[3] plots of the σ_{xx}, σ_{yy}, and σ_{zz} ^{31}P tensor components for the three P of **1-La** as a shielding surface. The shielding surfaces are represented using the ovaloid convention where the distance from the P atom to a point on the surface is proportional to the chemical shift when the magnetic field is aligned along that direction in space. The shading of the surface denotes the sign of the shift where orange is positive and light orange is negative. Key: pink = La; orange = P; beige = Si; red = O; gray = C. Hydrogen atoms are omitted for clarity.

Table S8. Calculated ^{31}P chemical shift anisotropy parameters for **1-La**, using a structure with optimized H positions and B3LYPHF30. δ_{11}, δ_{22}, δ_{33} = principal components of the chemical shift tensor; Span, $\Omega = \delta_{11}-\delta_{33}$; Skew, $\kappa = [3(\delta_{22}-\delta_{iso})]/\delta_{11}-\delta_{33}$).

	δ_{iso} (ppm)	δ_{11} (ppm)	δ_{22} (ppm)	δ_{33} (ppm)	Span Ω (ppm)	Skew κ
P_1	-50.3	341.5	-204.5	-288.0	629.5	-0.73
P_2	-211.0	50.3	-230.9	-452.4	502.7	-0.12
P_3	-65.7	281.1	-174.4	-303.9	585.0	-0.56

Despite the relative accuracy of average DFT calculated shifts, values for individual nuclei fail to accurately distinguish the differing degrees of pyramidalization present owing to the dynamics of the system.

7. UV-Vis-NIR Spectroscopy: 1-Ln

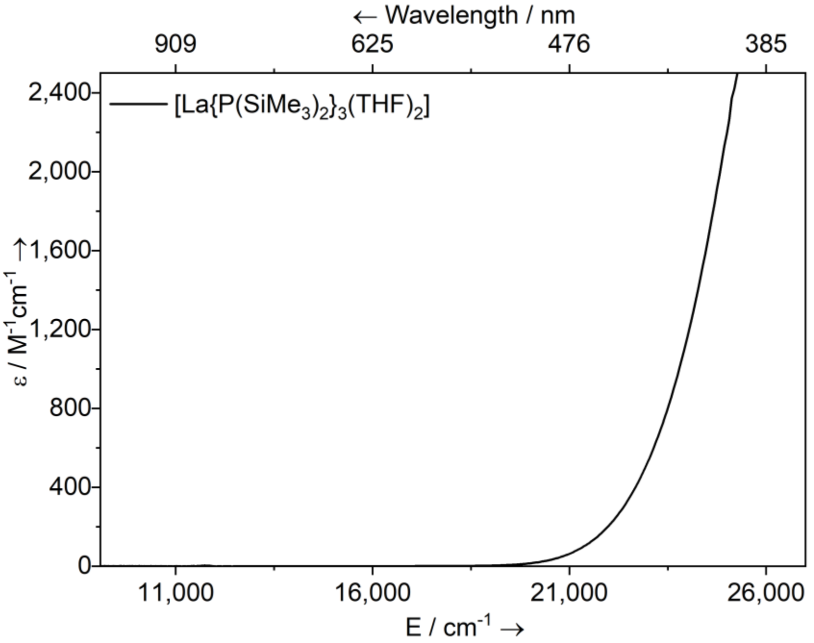

Figure S49. UV-Vis-NIR spectrum of **1-La** in toluene (2 mM) between 9091–27000 cm^{-1} (1100–370 nm).

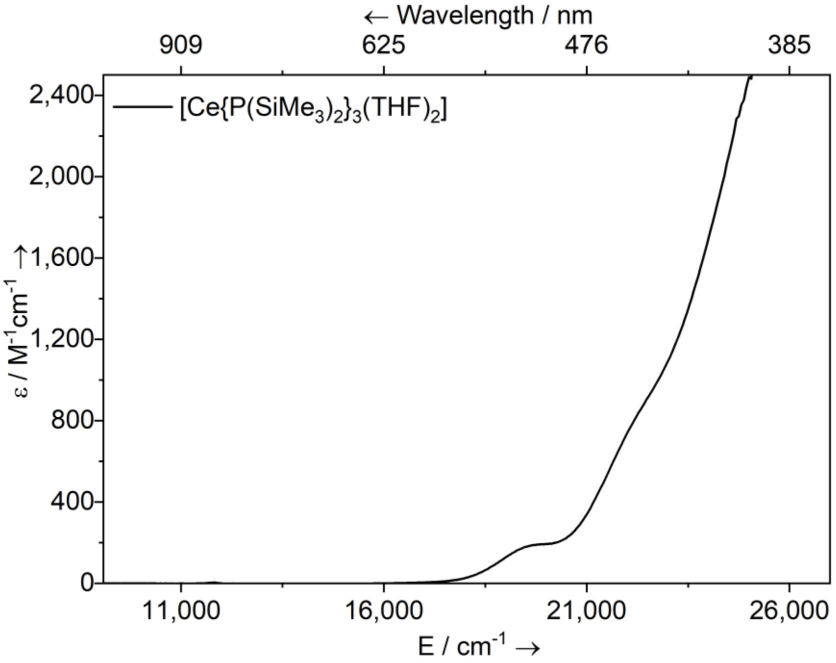

Figure S50. UV-Vis-NIR spectrum of **1-Ce** in toluene (2 mM) between 9091–27000 cm^{-1} (1100–370 nm).

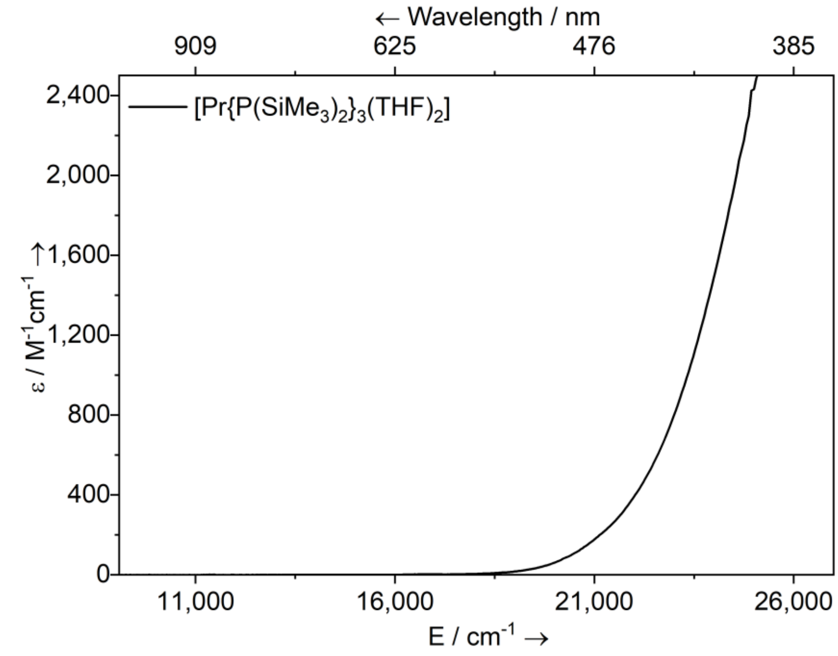

Figure S51. UV-Vis-NIR spectrum of **1-Pr** in toluene (2 mM) between 9091–27000 cm^{-1} (1100–370 nm).

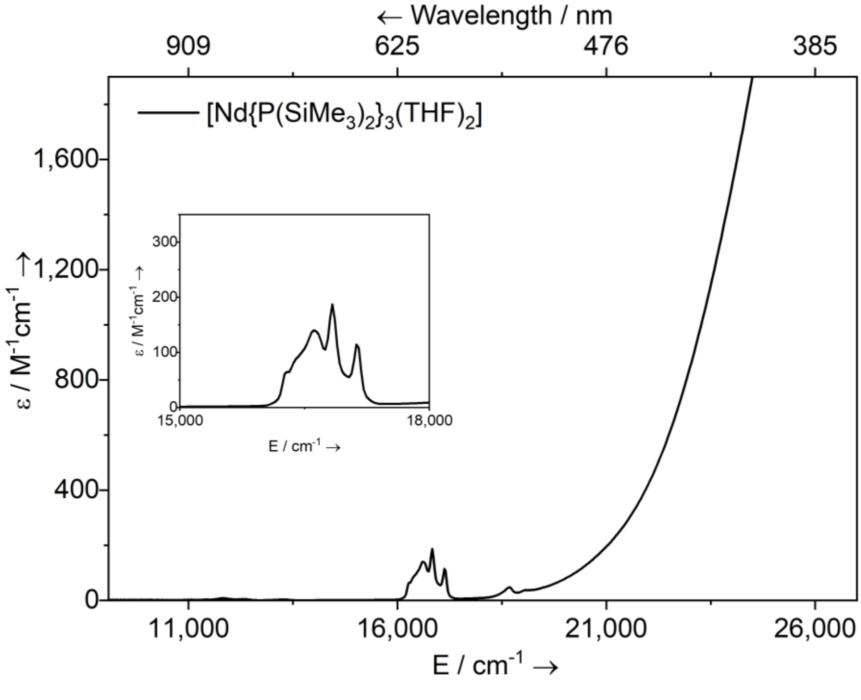

Figure S52. UV-Vis-NIR spectrum of **1-Nd** in toluene (2 mM) between 9091–27000 cm^{-1} (1100–370 nm). Inset shows region between 15000–18000 cm^{-1} (667–556 nm).

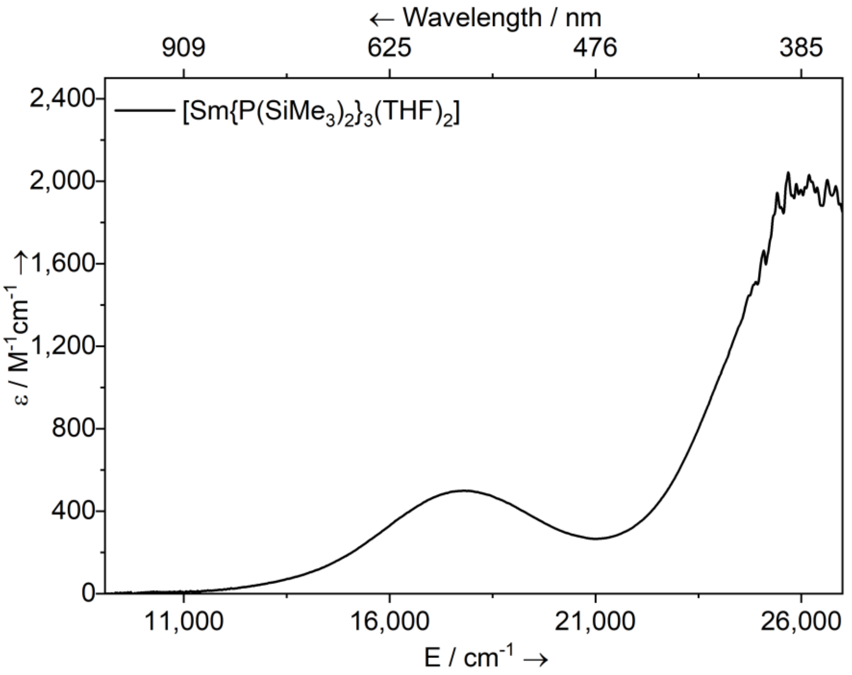

Figure S53. UV-Vis-NIR spectrum of **1-Sm** in toluene (2 mM) between 9091–27000 cm^{-1} (1100– 370 nm).

8. Magnetism: 1-Ce, 1-Pr, 1-Nd, 1-Sm

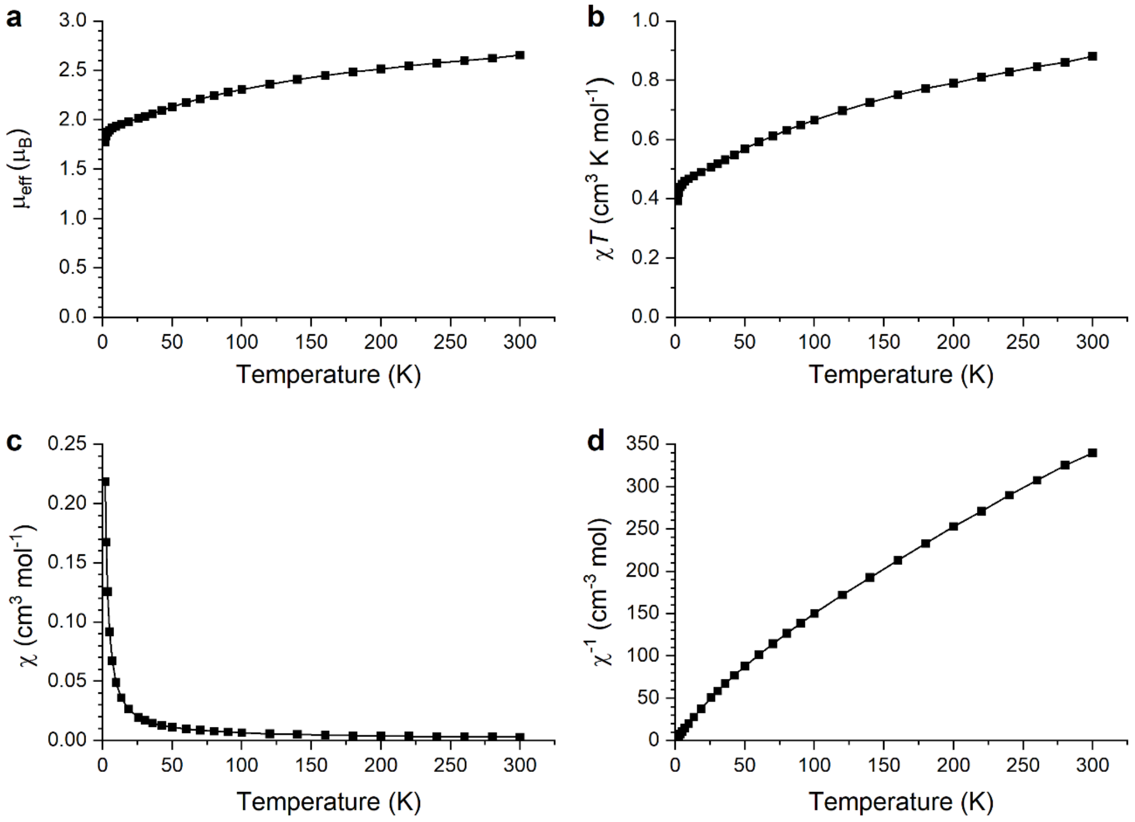

Figure S54. Variable-temperature SQUID magnetic data for powdered **1-Ce** in a 0.5 T applied magnetic field, presented as: **a** – μ_{eff} vs T; **b** – χT vs T; **c** – χ vs T; **d** – χ^{-1} vs T.

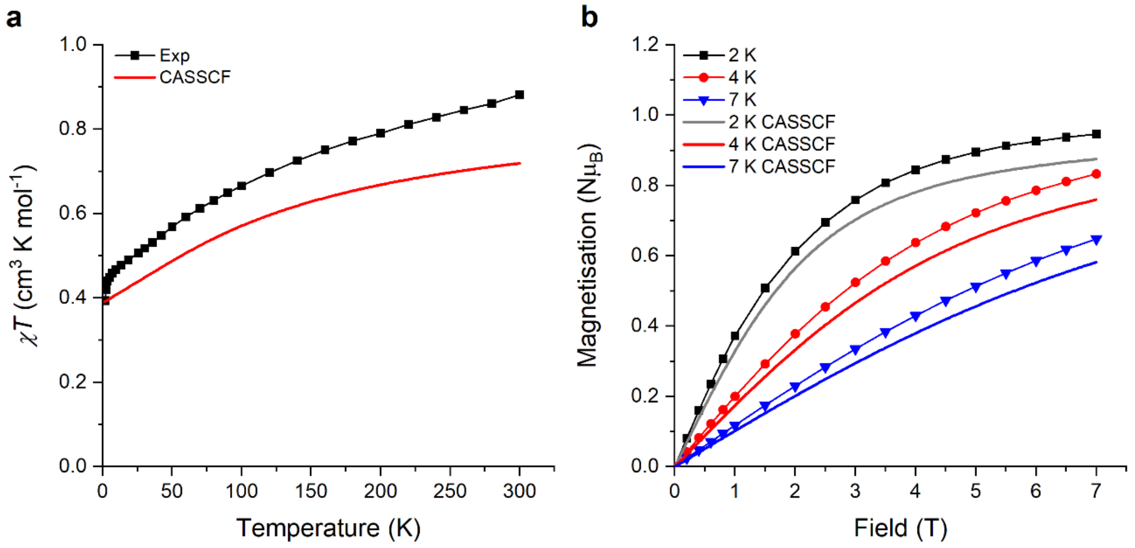

Figure S55. a – Magnetic susceptibility plot and **b** - Magnetization vs. field plot for **1-Ce**. Solid lines show CASSCF results.

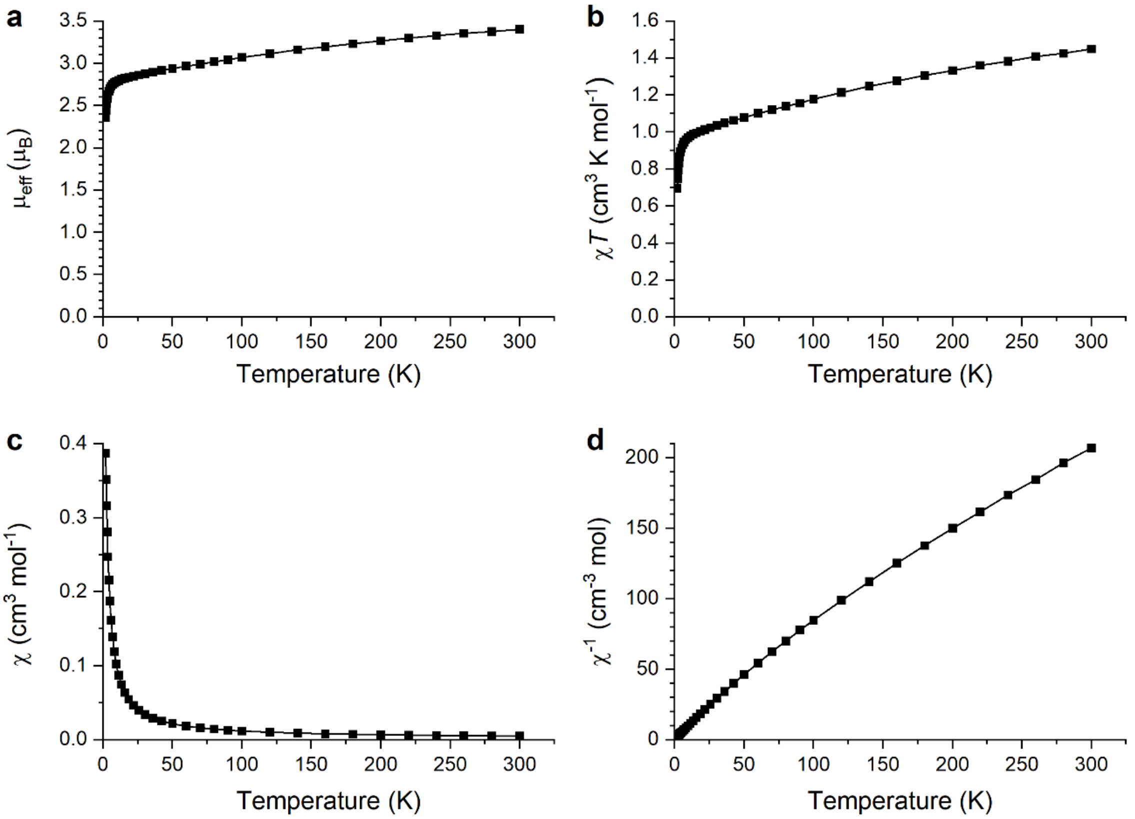

Figure S56. Variable-temperature SQUID magnetic data for powdered **1-Pr** in a 0.1 T applied magnetic field, presented as: **a** – μ_{eff} vs T; **b** – χT vs T; **c** – χ vs T; **d** – χ^{-1} vs T.

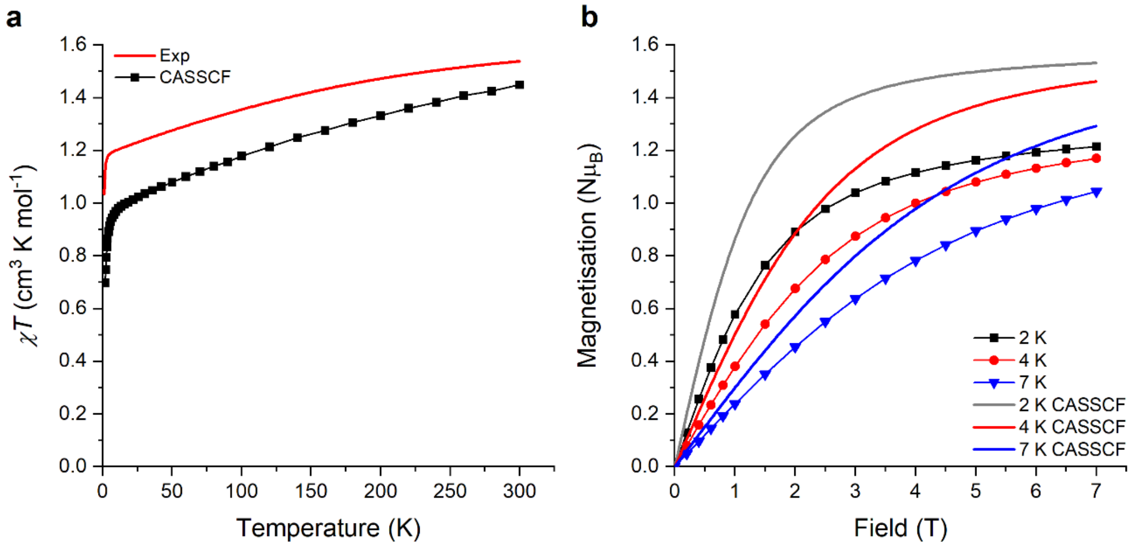

Figure S57. a – Magnetic susceptibility plot and **b** - Magnetization vs. field plot for **1-Pr**. Solid lines show CASSCF results.

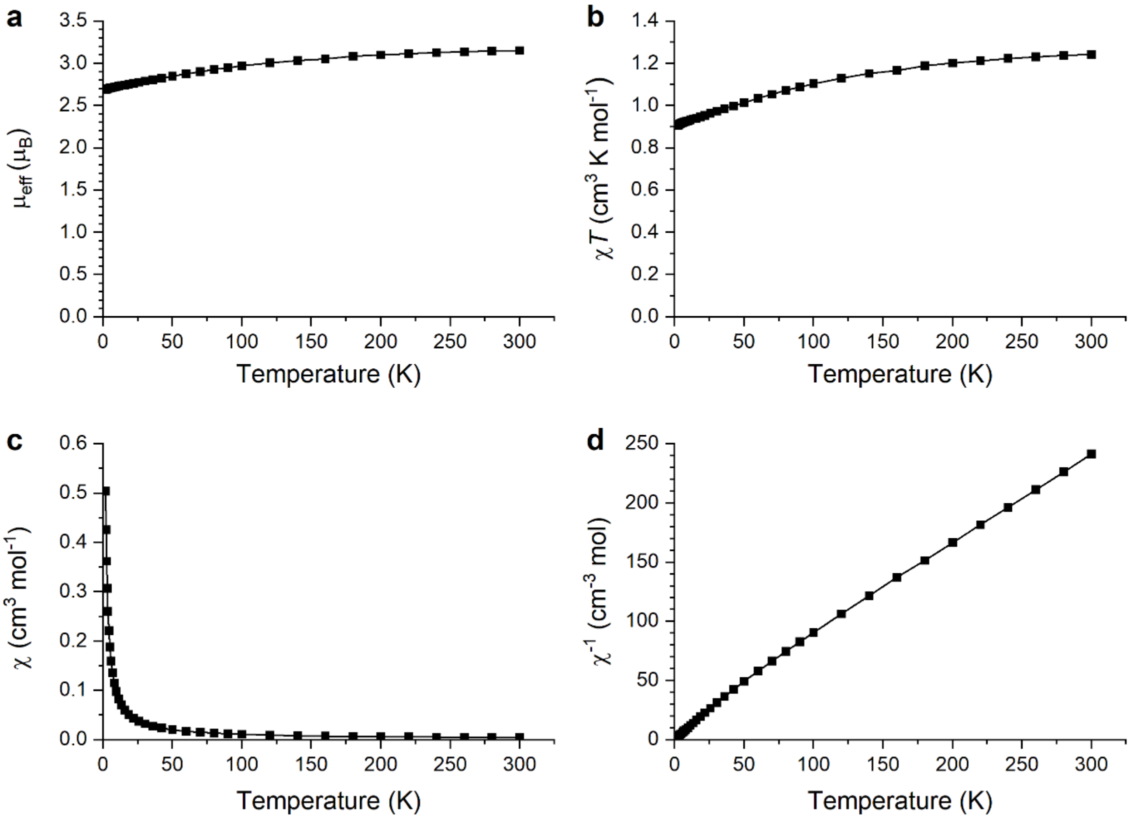

Figure S58. Variable-temperature SQUID magnetic data for powdered **1-Nd** in a 0.1 T applied magnetic field, presented as: **a** – μ_{eff} vs T; **b** – χT vs T; **c** – χ vs T; **d** – χ^{-1} vs T.

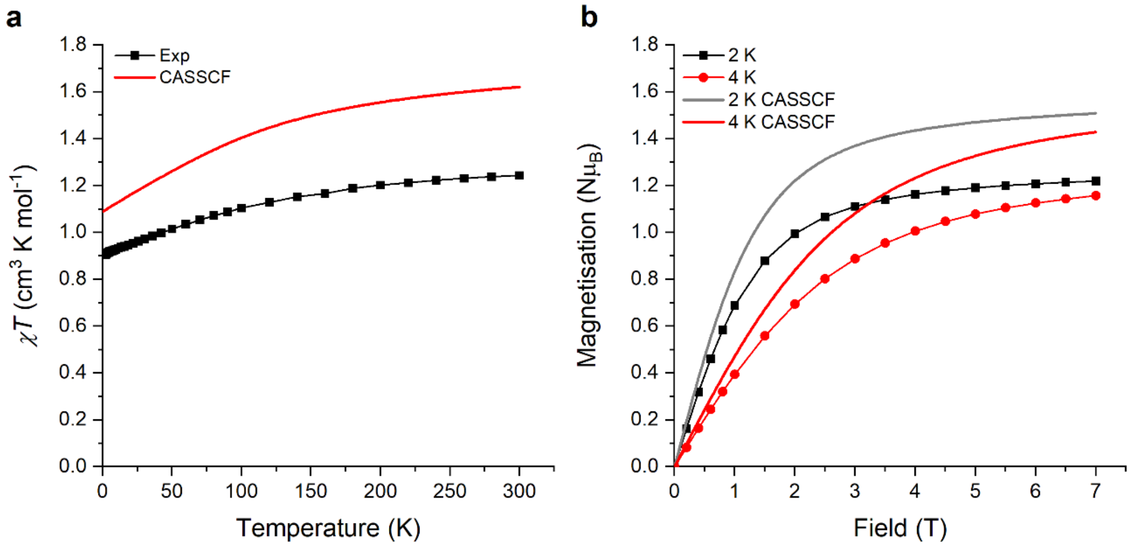

Figure S59. a – Magnetic susceptibility plot and **b** - Magnetization vs. field plot for **1-Nd**. Solid lines show CASSCF results.

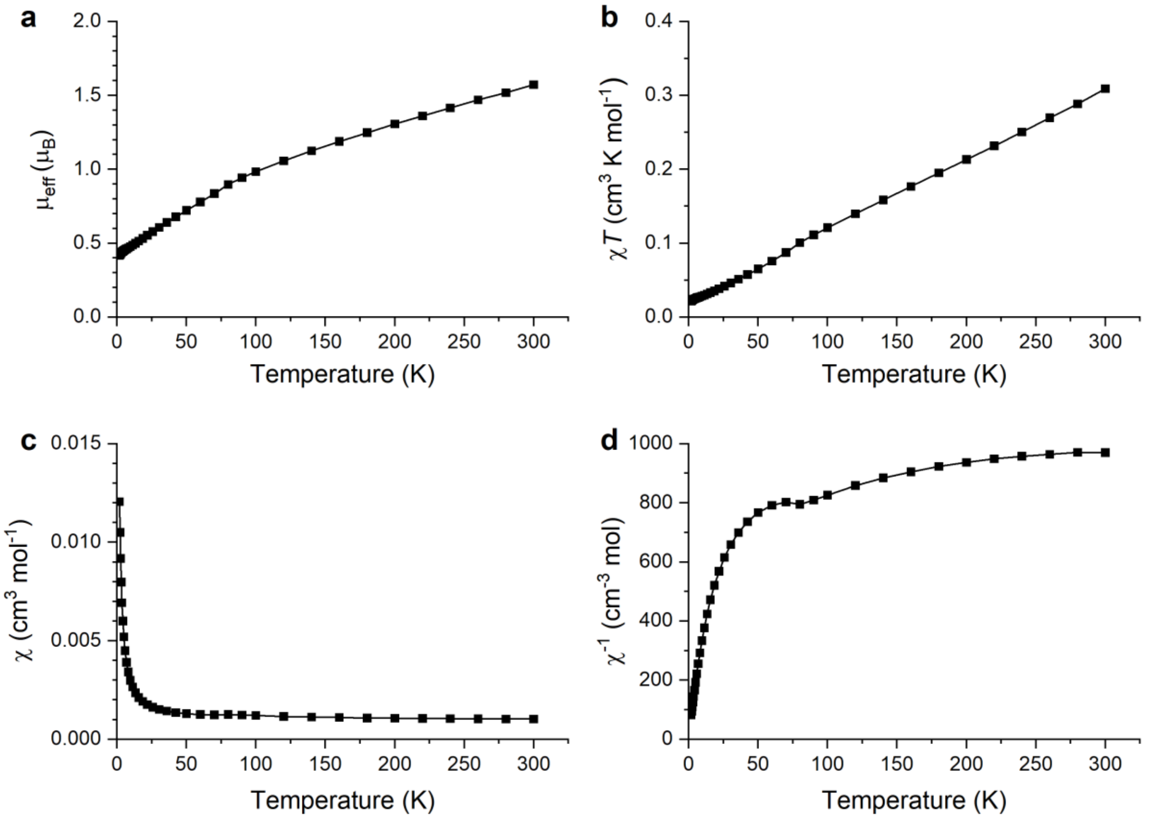

Figure S60. Variable-temperature SQUID magnetic data for powdered **1-Sm** in a 1 T applied magnetic field, presented as: **a** – μ_{eff} vs T; **b** – χT vs T; **c** – χ vs T; **d** – χ^{-1} vs T.

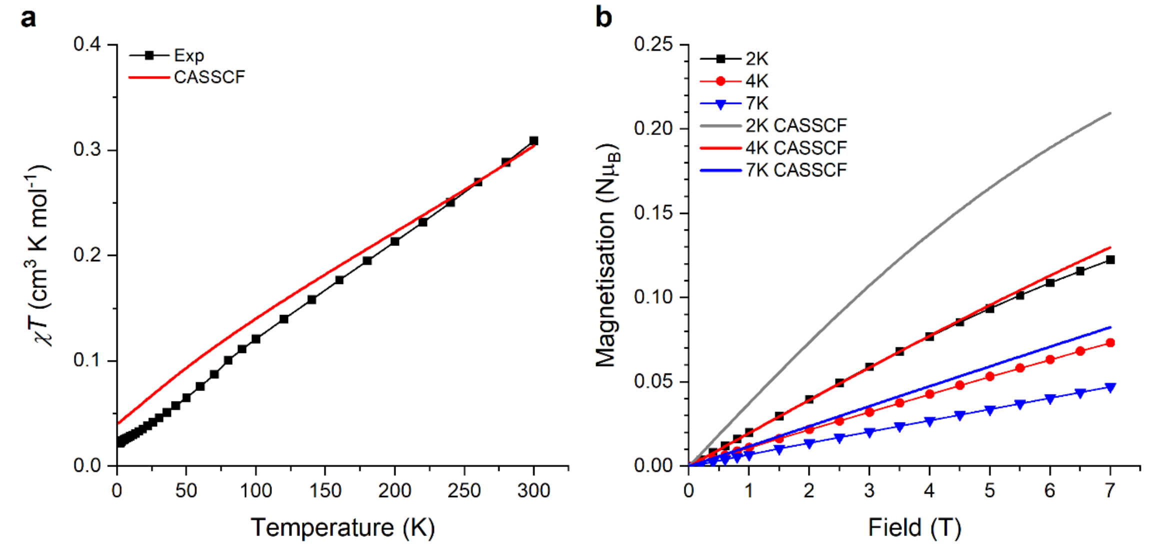

Figure S61. a – Magnetic susceptibility plot and **b** - Magnetization vs. field plot for **1-Sm**. Solid lines show CASSCF results.

9. EPR Spectroscopy: 1-Ce, 1-Nd

Table S9. Parameters used in EPR simulations of **1-Ce** and **1-Nd**.

	g_{xy}	g_z	$gStrain_{xy}$ [b]	$gStrain_z$	A_z / Mz
**1-Ce**	0.31[a]	3.598	0.1	0.165	n/a
**1-Nd**	0.27[a]	5.959	0.05	0.6	1795

[a] *g*-feature lies outside field window and value is approximate.

[b] Strain parameters for *xy* *g*-values are purely phenomenological to reproduce to broad rises towards high-field in the spectra: these values do not represent real distributions.

10. Density Functional Theory (DFT) Calculations

Table S10. Coordinates of the XRD structure of $[La\{P(SiMe_3)_3\}_2(THF)_2]$ (**1-La**) used for calculations, in .xyz format with all coordinates in angstrom.

```
108
[La{P(SiMe3)2}3(thf)2]
La     1.98411     3.68436     9.75774
P      0.74407     2.15054     7.65789
P      3.38068     2.76010    12.16590
P      1.64527     6.50664     9.71836
Si     1.18683     7.62285     7.86253
Si     0.92497     0.08093     6.87718
Si    -1.37888     2.73649     7.38085
Si     2.20713     7.94577    11.29021
Si     5.52342     3.32272    12.01165
Si     3.40751     0.53996    12.12188
O      0.00355     3.32528    11.23210
O      3.97938     3.83516     8.24246
C     -1.64873     4.34343     8.30578
H     -0.93975     4.97778     8.07257
H     -1.63018     4.17198     9.27051
H     -2.51918     4.72039     8.05807
C      2.73421    -0.38039     6.65150
H      3.20343    -0.29063     7.50702
H      3.14326     0.21588     5.99015
H      2.79829    -1.30730     6.33940
C     -2.61727     1.44061     7.95770
H     -3.52297     1.81333     7.91702
H     -2.41264     1.18151     8.88087
H     -2.56130     0.65354     7.37748
C     -0.53951     8.34288     7.91484
H     -0.72591     8.80752     7.07235
H     -0.60727     8.97663     8.65930
H     -1.19114     7.62120     8.04046
C     -0.13131     4.15188    12.42112
H     -0.86817     4.80514    12.31754
H      0.70843     4.64127    12.61033
C      5.28429     4.75484     6.52196
H      6.13115     5.23422     6.33842
H      4.60489     5.02881     5.85665
C      2.40200     9.01733     7.52719
H      3.31158     8.65473     7.48022
H      2.35047     9.67758     8.24906
H      2.17879     9.44776     6.67464
C      4.80726     5.00843     7.92565
H      5.57157     5.08089     8.55107
H      4.27382     5.84088     7.97251
C      1.29362     6.44230     6.40323
H      1.00992     6.90432     5.58713
H      0.70780     5.67207     6.56250
H      2.21761     6.13296     6.29956
```

C	-1.35987	2.17981	12.82113
H	-2.30639	2.44355	12.94024
H	-1.23662	1.26391	13.17762
C	-0.43866	3.15505	13.52191
H	0.38327	2.70714	13.84265
H	-0.89201	3.59128	14.28670
C	4.32822	2.76118	7.31290
H	3.56334	2.55268	6.72007
H	4.57732	1.94065	7.80837
C	-1.79443	3.07007	5.57927
H	-2.74259	3.30277	5.50033
H	-1.60860	2.26835	5.04745
H	-1.24681	3.81396	5.25052
C	0.16661	-1.19512	8.01562
H	-0.76355	-0.95230	8.20537
H	0.67348	-1.22885	8.85309
H	0.19211	-2.07489	7.58281
C	6.41209	3.07142	13.64701
H	6.45344	2.11372	13.85304
H	5.92625	3.53759	14.35875
H	7.32213	3.43044	13.58346
C	3.90163	8.68415	11.03885
H	4.12992	9.24625	11.80865
H	3.90285	9.22886	10.22460
H	4.56140	7.96490	10.95248
C	5.49006	3.25892	6.51172
H	5.46774	2.90100	5.58838
H	6.34953	3.01194	6.93482
C	-0.96486	2.24320	11.35758
H	-0.56185	1.38527	11.07140
H	-1.75807	2.42329	10.79371
C	0.10924	-0.06879	5.18737
H	0.34632	-0.93060	4.78545
H	0.42231	0.65808	4.60832
H	-0.86321	-0.01076	5.28739
C	5.59890	5.16895	11.65083
H	6.52248	5.42513	11.44702
H	5.28678	5.66837	12.43440
H	5.02571	5.37577	10.88286
C	6.49232	2.42126	10.69135
H	7.41905	2.74194	10.68961
H	6.08742	2.59193	9.81477
H	6.47997	1.45947	10.87520
C	1.81702	-0.12275	12.84970
H	1.84101	-1.10246	12.85001
H	1.05806	0.18508	12.31127
H	1.71807	0.20187	13.76906
C	4.80890	-0.21312	13.09488
H	4.79126	0.13024	14.01239
H	5.66195	0.02112	12.67408
H	4.70890	-1.18851	13.10951
C	2.23470	7.02502	12.92499
H	2.76310	6.20595	12.83018
H	1.31766	6.79405	13.18498
H	2.63487	7.59507	13.61437
C	1.02032	9.38153	11.42689
H	1.32256	9.98862	12.13362

H	0.12583	9.04606	11.64326
H	0.99119	9.86264	10.57305
C	3.49702	-0.13624	10.37474
H	3.49485	-1.11646	10.40312
H	4.31954	0.17604	9.94446
H	2.72129	0.17621	9.86305

Table S11. Coordinates of the B3LYPHF20 H-atom geometry optimized structure of $[La\{P(SiMe_3)_3\}_2(THF)_2]$ (**1-La**) , in .xyz format with all coordinates in angstrom.

108
Coordinates from ORCA-job La_optH

La	1.98446678292785	3.68397619177786	9.75982153187895
P	0.74500809809602	2.14969052952542	7.65996850010999
P	3.38128148180161	2.76024169916862	12.16804125537402
P	1.64466935630527	6.50614010586394	9.72037187436349
Si	1.18590355316057	7.62215494331181	7.86450501176129
Si	0.92663341099526	0.08012550515102	6.87930762162738
Si	-1.37813284691370	2.73491355347260	7.38285511092362
Si	2.20599539171153	7.94549422318148	11.29220744058771
Si	5.52383469835302	3.32358616281719	12.01384082742769
Si	3.40886678150222	0.54011000731081	12.12406916116319
O	0.00398652739214	3.32425481694063	11.23413230138705
O	3.97972894624339	3.83542171628096	8.24459560859090
C	-1.64855513864158	4.34178144377009	8.30774324416555
H	-1.01392172466708	5.13105579526695	7.90467845465974
H	-1.43575696180324	4.27449580864021	9.37449714779407
H	-2.68566276474489	4.67137655188733	8.19487421012348
C	2.73603648846252	-0.38058468737406	6.65368938126616
H	3.29939665367806	-0.30856550356540	7.58354932774513
H	3.21954802182464	0.25141455712773	5.90605931302513
H	2.80166861122151	-1.41543821075818	6.30554356835453
C	-2.61609921162172	1.43862525964773	7.95969705135529
H	-3.63358666994337	1.83788341534212	7.90808043116892
H	-2.43237582822883	1.10081412407084	8.97987794727917
H	-2.57616136256517	0.56015677948919	7.31229703341489
C	-0.54068240842277	8.34159966145723	7.91675016725710
H	-0.77477049409452	8.87740277620422	6.99169981604591
H	-0.64771863756289	9.04037332444144	8.74870120408711
H	-1.28375414858877	7.55363666169484	8.05171883814805
C	-0.13118834962004	4.15083420250635	12.42313087522261
H	-0.93712057241677	4.87007303554828	12.25433603151194
H	0.80597939282832	4.68306334060319	12.55872939326665
C	5.28437591256426	4.75550838128259	6.52411354473015
H	6.18886318349654	5.31847942491393	6.29351263419942
H	4.51336645155329	5.04410283222009	5.80581375089618
C	2.40060948289664	9.01704047348619	7.52917028848895
H	3.42761155485076	8.64802780977627	7.49455653402359
H	2.35223851726708	9.79132440437770	8.29705176271798
H	2.17657257470687	9.48883280348425	6.56777175513870
C	4.80721949365365	5.00896611679376	7.92778446484682
H	5.63167874325546	5.03814867547254	8.64212426912755
H	4.17780545073487	5.88513196071178	8.06686727120222

C	1.29313641834324	6.44161030872389	6.40523314532582
H	0.92771914342542	6.93108170410782	5.49789046684453
H	0.71110038763460	5.53002198729825	6.54864434541945
H	2.32662049212651	6.13817836601232	6.22355616763339
C	-1.35908997541268	2.17835554136116	12.82314748978158
H	-2.40138133923953	2.47855746007468	12.94291615553776
H	-1.26515696705464	1.16690935763388	13.21726373350643
C	-0.43823136613854	3.15392323115721	13.52393321521102
H	0.49272204233265	2.67617503953338	13.83191282177655
H	-0.89283270201729	3.62610550119356	14.39403732392586
C	4.32896039347512	2.76154054631569	7.31506842869272
H	3.44327880311342	2.57492148066169	6.70789815460822
H	4.54469273691871	1.87032176089811	7.90297604122449
C	-1.79374424432951	3.06831413423473	5.58125606367673
H	-2.83480373096442	3.39168414911595	5.48255375335840
H	-1.65683762939700	2.18083928337288	4.96388426685043
H	-1.15543345879217	3.85579531282082	5.17501452192889
C	0.16867416005818	-1.19615777068917	8.01775300476846
H	-0.88881038191933	-0.99541050511779	8.19565927263164
H	0.67353691754774	-1.19859190468563	8.98627188670614
H	0.25538888447730	-2.20033708415365	7.59139933485226
C	6.41254301865428	3.07262271997027	13.64923168574315
H	6.47818143192638	2.01929512086571	13.92356811478953
H	5.88266857613272	3.59189572776564	14.45027418129284
H	7.43060203265941	3.47142310406118	13.60013553147144
C	3.90025172160264	8.68444437752494	11.04088040641309
H	4.13217116888992	9.38894824003796	11.84596526780040
H	3.96504106816506	9.22535071193605	10.09509678226659
H	4.67333965391747	7.91448133195283	11.03675093208303
C	5.49065427556462	3.25965814050653	6.51391121502542
H	5.50148329666260	2.83116352793912	5.51202646663650
H	6.43649878059060	3.00324057212529	6.99737442057815
C	-0.96405950191685	2.24184862817929	11.35960748453494
H	-0.47613200378355	1.34395902862623	10.98677379738580
H	-1.80596748230640	2.46812911362592	10.70695876138810
C	0.11100283141864	-0.06990744514614	5.18947739039076
H	0.32796312516975	-1.05002428074764	4.75421848472653
H	0.47975250473944	0.69702875030353	4.50656192747976
H	-0.97378538672018	0.02948776192222	5.25173726145176
C	5.59869791628212	5.16983403677586	11.65298378948206
H	6.62708743595641	5.48821594239242	11.45937535924384
H	5.23411595303922	5.73307563475259	12.51426690584084
H	4.98727062831029	5.46386998588967	10.79828399063710
C	6.49307870997547	2.42242727515815	10.69358776923056
H	7.53540815675874	2.75548197417855	10.68636408400411
H	6.07270494645905	2.60984872845052	9.70368743493499
H	6.49258150820383	1.34292641099557	10.85200653069564
C	1.81858099526170	-0.12312455389197	12.85185757223489
H	1.83064393926306	-1.21669963815209	12.85649950993160
H	0.95122830107398	0.19132035390428	12.26807859027934
H	1.67197774188516	0.21842206093519	13.87858916525283
C	4.81048446097674	-0.21247350131604	13.09712535958858
H	4.79789420008562	0.12651011263072	14.13473493482956
H	5.78844482053741	0.03126146978097	12.68020324697547
H	4.71507018821714	-1.30262820869239	13.09821973498050
C	2.23383117330221	7.02478833375994	12.92700778261810
H	2.79132781002661	6.08800941530036	12.86291182545809

H	1.22353694134267	6.79641079983117	13.27202796151482
H	2.71076008878866	7.64010841831698	13.69544892477815
C	1.01869388518431	9.38085394742420	11.42882288092539
H	1.30701643692377	10.02927542173086	12.26167345709323
H	-0.00620199592062	9.04499863056323	11.59863556377994
H	1.02090767657708	9.98944306421792	10.52196150122260
C	3.49865659180963	-0.13609663837797	10.37694609303404
H	3.46541094743357	-1.22971355576673	10.36048141348232
H	4.41973464671052	0.17788259361704	9.88129945061182
H	2.65904975833313	0.22268778095044	9.77519350516197

Table S12. Coordinates of the B3LYPHF20 fully geometry optimized structure of [La{P($SiMe_3$)$_3$}$_2$(THF)$_2$] (**1-La**) , in .xyz format with all coordinates in angstrom.

108
Coordinates from ORCA-job La_opt

La	1.97930156567226	3.64441257762859	9.85043676952973
P	0.71041169906020	2.22086103399364	7.69428717868860
P	3.30672920708405	2.71994686388200	12.30514310751626
P	1.48750398667229	6.43398918728047	9.77586680766779
Si	1.35753727561694	7.35846877210310	7.75797069525856
Si	0.80535111786045	0.09118902716057	7.05476229938122
Si	-1.42339092878857	2.81126402435495	7.43995973599686
Si	2.16047146156923	7.99077816057168	11.20966422202049
Si	5.43178620437254	3.36261940269234	12.12585549032349
Si	3.42321367674589	0.53330893917033	11.91443975991577
O	-0.04438606106914	3.25182006451914	11.31922193683496
O	4.03158823529937	3.90973852554446	8.34997525802659
C	-1.76064312489364	4.38940801828587	8.41663981493947
H	-1.15636717536984	5.22203356036633	8.05631126095370
H	-1.55430342939023	4.28769806043161	9.48180727392738
H	-2.81111527366482	4.67252521346009	8.30136519345376
C	2.58148215943740	-0.31201268717666	6.56634131071987
H	3.28706879984655	-0.13148153117612	7.37617086486473
H	2.88257072877240	0.29375291075161	5.70869363497925
H	2.66379199460534	-1.36450176485532	6.28006840238291
C	-2.64786209631371	1.49256421560202	8.01646350202327
H	-3.67008179387870	1.87681555097101	7.94877029754091
H	-2.46615062579822	1.19122290100820	9.04810668667392
H	-2.59218129106444	0.59555562139041	7.39844731698772
C	-0.25637303035079	8.29996083907743	7.52883873208681
H	-0.32307981245860	8.72139223175089	6.52125505063186
H	-0.33787579592465	9.11926319437871	8.24549885909358
H	-1.11442129132876	7.64265095359474	7.68105308608259
C	-0.14015250860419	3.98562590430925	12.56259554365061
H	-0.97559822873218	4.68772594902064	12.48913327640571
H	0.78705481999756	4.53876500421543	12.69357995031561
C	5.34706351375556	5.16839567460252	6.85469843676928
H	6.27452020713325	5.73172030274781	6.75898268399938
H	4.59164087258841	5.64035298300865	6.22520671623653
C	2.77280561564772	8.55410675813033	7.40042636629631
H	3.74639214733534	8.07995954002707	7.53602353917249
H	2.73449563497360	9.42821441090581	8.05249895240804
H	2.70871874278598	8.90853963718331	6.36724828220493

C	4.87278008297480	5.09887717320171	8.29303236371556
H	5.70479938495024	4.96052835989455	8.98486058496799
H	4.25981772033455	5.93594215907259	8.62183018574202
C	1.43292704239616	5.99155639637024	6.46211341845060
H	1.33544639812004	6.41940362367756	5.46003007342995
H	0.64350428936128	5.25054252805299	6.58560761835209
H	2.37929184082855	5.45190281518615	6.50476383790406
C	-1.24955443010178	1.88396934364220	12.86194698602634
H	-2.30125480484516	2.03512740221124	13.10512838445580
H	-0.99015076346878	0.85995872476475	13.12286753354850
C	-0.35753512372577	2.90089144814545	13.59907977464640
H	0.60746208105010	2.46171372128709	13.85281301422476
H	-0.81727125485009	3.28388600903217	14.50933602226662
C	4.34108461848319	3.02831915333269	7.22404424031897
H	3.43863955424198	2.95588302851102	6.61749001089314
H	4.57966372693668	2.04352925257067	7.62243789719512
C	-1.80492535169407	3.18572916807780	5.63388846570369
H	-2.84731575346007	3.49566554963766	5.51189717674561
H	-1.63439205363769	2.31244858462700	5.00315056255823
H	-1.16504838257589	3.99039178991728	5.26645578651812
C	0.25101955699068	-1.09243479885436	8.41449673231157
H	-0.78862922892510	-0.89821242378414	8.68539151237247
H	0.85791051223680	-0.97337936543568	9.31353577175045
H	0.32695466596929	-2.13453656111551	8.08986274135726
C	6.41742704855542	2.97405493702536	13.68313236876868
H	6.49252928612837	1.89928808265917	13.85319608284109
H	5.93700050756399	3.41672622226289	14.55792344793994
H	7.43209227082636	3.37725874153671	13.60980226982237
C	3.92394861404059	8.60026260863547	10.94311443590244
H	4.17049431993508	9.36221586537050	11.68908691056983
H	4.04257618424228	9.04832214984385	9.95615979871807
H	4.64858409241410	7.79222166510994	11.03884870692480
C	5.50687302374108	3.68687654440792	6.49680699679199
H	5.46814714135468	3.49954212927007	5.42461475300729
H	6.45778529957595	3.30727183789514	6.87506283474524
C	-1.00491702393383	2.16867728287728	11.37094070564575
H	-0.57673349040577	1.33884283275066	10.81356121438849
H	-1.91176873548827	2.49635522848243	10.86216273455523
C	-0.24542016306705	-0.24445406180482	5.52552574120431
H	-0.09578668816026	-1.27809044529417	5.19948550909725
H	0.04473279276478	0.41409539093613	4.70492558984829
H	-1.31124785621257	-0.10593254689314	5.70748600214379
C	5.40510213997630	5.23810409240890	11.92822057494562
H	6.40453100556519	5.63443265667376	11.72782313179109
H	5.03671519397828	5.70904292405957	12.84177187903009
H	4.74722370989795	5.55477431790291	11.11835916767641
C	6.37938236975275	2.61519262120622	10.66949201041760
H	7.36644112283889	3.07861350507017	10.57849023729128
H	5.84527997187360	2.75458954889307	9.73019426168605
H	6.53111292284446	1.54352903109061	10.80938346179116
C	1.79259987131709	-0.25270096690483	12.44406647068021
H	1.81589216118247	-1.33478921927976	12.28891634428916
H	0.95952304202441	0.14374944388368	11.86198920414776
H	1.58864829087009	-0.06380424445213	13.49973024662508
C	4.81320634537593	-0.33547083475310	12.84201093809061
H	4.74441358077780	-0.14123177867962	13.91398789120211
H	5.79718309020775	-0.00586771103310	12.50560903280913

H 4.75212812542740 -1.41651821146801 12.68625554310041
C 2.03815406610522 7.23882888616031 12.93363842075725
H 2.60475297703211 6.30839153842978 13.00766083004875
H 0.99786382324924 7.02487810512628 13.18706962539665
H 2.43174560828737 7.92971884477276 13.68421796889896
C 1.03202894578215 9.49650640064237 11.11797571740796
H 1.30338913620893 10.23271929626769 11.88028851592749
H -0.01147887689051 9.21378362965122 11.26717448855746
H 1.10911981775942 9.98366493954979 10.14358425494396
C 3.61940353737044 0.16012178658630 10.07249976474861
H 3.64011996511363 -0.91511188184738 9.87354420202351
H 4.53729611905883 0.59514857942312 9.67547619253772
H 2.77673378634562 0.56528712261129 9.50409452884438

Table S13. Coordinates of the B3LYPHF30 H-atom geometry optimized structure of [La{P(SiMe$_3$)$_3$}$_2$(THF)$_2$] (**1-La**) , in .xyz format with all coordinates in angstrom.

108
Coordinates from ORCA-job La_optH
La 1.98453200099605 3.68395595990058 9.75980366103502
P 0.74506237975077 2.14967154102223 7.65995618152773
P 3.38138344252128 2.76021982096823 12.16800143498016
P 1.64470865815497 6.50611704276489 9.72037470449766
Si 1.18591089319316 7.62213871922371 7.86451985570591
Si 0.92669715379284 0.08011277775761 6.87928092222194
Si -1.37808710852657 2.73487706113848 7.38287131304187
Si 2.20604025928864 7.94546692571196 11.29221216057898
Si 5.52392975725686 3.32358451969406 12.01377905165281
Si 3.40898825040778 0.54008863764282 12.12401588878562
O 0.00407238315208 3.32420800785905 11.23413567198454
O 3.97977492316052 3.83542843635850 8.24455508838381
C -1.64851299014691 4.34173704381179 8.30777213082627
H -1.02077882661093 5.12940111800717 7.90157953883581
H -1.43218869775130 4.27646920007497 9.36987205666164
H -2.68371777856173 4.66722731698546 8.19981727771614
C 2.73610171857811 -0.38057978367532 6.65363859627440
H 3.29902681219706 -0.30754829114053 7.57910830596014
H 3.21697068738894 0.24767017862892 5.90632554567220
H 2.80140523478617 -1.41270147288105 6.30822317525093
C -2.61603505068357 1.43857425045232 7.95972016725935
H -3.62937634668310 1.83973156075998 7.91541989021410
H -2.42941501187066 1.09590664935922 8.97379695497027
H -2.58262753015451 0.56650162446146 7.30944394453487
C -0.54068093984079 8.34156755754107 7.91678963831977
H -0.77423988173077 8.87362497827997 6.99355681968293
H -0.64699210979745 9.04046453774321 8.74399181641182
H -1.28173717284878 7.55699330084631 8.05297041769818
C -0.13109592000284 4.15077914284713 12.42314072986646
H -0.93486234687503 4.86588559357255 12.25472212294357
H 0.80149346752320 4.68129208536442 12.55962872852091
C 5.28439328878276 4.75553703891439 6.52406307034574
H 6.18629796180917 5.31633024030099 6.29547324986378
H 4.51670655268585 5.04303307460385 5.80791108735671

C	2.40060028683658	9.01703718714519	7.52917905022815
H	3.42434702807090	8.64944594337999	7.49475966238199
H	2.35200551725033	9.78983370808177	8.29332858800612
H	2.17770276432895	9.48626386944477	6.57024548502850
C	4.80725115031857	5.00898217069958	7.92774112111268
H	5.62855374102843	5.03681020586409	8.63884612582517
H	4.18178366792513	5.88246512058690	8.06455837390317
C	1.29313718672683	6.44160368247669	6.40523974181843
H	0.93230211703068	6.93103039835091	5.50027026591058
H	0.70951505266592	5.53539782174731	6.54621357128032
H	2.32248860849065	6.13632522460275	6.22563018968031
C	-1.35897503360983	2.17828704037131	12.82316016694833
H	-2.39746510692629	2.47852398925260	12.94183031052145
H	-1.26600375175794	1.17027396182577	13.21560231635327
C	-0.43811696076169	3.15385889631727	13.52394079878121
H	0.48842328213178	2.67762373403157	13.83254112098767
H	-0.89278059593147	3.62515780891935	14.38990441525865
C	4.32900508703214	2.76155591071931	7.31501743567752
H	3.44835219605085	2.57727292115827	6.70861475884511
H	4.54403627451812	1.87410880999106	7.90061089757570
C	-1.79372279558710	3.06828457083999	5.58127915162709
H	-2.83032963284961	3.39456895248881	5.48375987683871
H	-1.66185589159438	2.18305819889830	4.96614846192735
H	-1.15541834834805	3.85080887559986	5.17499627973865
C	0.16876284886881	-1.19618408758081	8.01772767882121
H	-0.88719144098234	-1.00117113836394	8.18869550910507
H	0.66652410249057	-1.19464045132063	8.98581437490660
H	0.26208925453251	-2.19771042399262	7.59554057650936
C	6.41265963563636	3.07261941634030	13.64915793981717
H	6.48148965773660	2.02281412013727	13.92164819034165
H	5.88293182601822	3.58807965487532	14.44802178955354
H	7.42634938659126	3.47313597271982	13.60134947727467
C	3.90028695958866	8.68443384569202	11.04086950644916
H	4.13187035778186	9.38530645712411	11.84449324299715
H	3.96471566584748	9.22550361685733	10.09938454158012
H	4.67096895007868	7.91739919526608	11.03531492565139
C	5.49068502414956	3.25968871847741	6.51384945952271
H	5.50053612464244	2.83272341352717	5.51537945781675
H	6.43317493764766	3.00405439269657	6.99544608747233
C	-0.96396240419576	2.24179234749452	11.35961587558719
H	-0.47884672551824	1.34673786509715	10.98918111237709
H	-1.80290897373175	2.46851570891247	10.71094536191662
C	0.11104798142864	-0.06991750830934	5.18945942874967
H	0.32906736675135	-1.04629040612745	4.75510088545284
H	0.47753279213330	0.69519230447829	4.50894190447670
H	-0.97029868894406	0.02667078872433	5.25121859197387
C	5.59877209519710	5.16983520386884	11.65293205457791
H	6.62337630202990	5.48631623650697	11.45608190295605
H	5.23968498497110	5.73117087685865	12.51312604444623
H	4.98702699425627	5.46326734484564	10.80338351950635
C	6.49316633035050	2.42244218407257	10.69350923346047
H	7.53095515615806	2.75810722296316	10.68466644442402
H	6.07325491548664	2.60549330674907	9.70693057971440
H	6.49723977254749	1.34703500087336	10.85417682573626
C	1.81871705048338	-0.12316460721861	12.85181914534189
H	1.82935797531835	-1.21325944945690	12.85376594621224
H	0.95387548709250	0.19233330887173	12.27212778100250

H 1.67453429548042 0.21455479169938 13.87640731139076
C 4.81062422704731 -0.21248792584795 13.09705110255815
H 4.79733406673343 0.12501453175203 14.13142581882784
H 5.78580621812378 0.02899265252178 12.68216076582423
H 4.71438745777868 -1.29905725118391 13.09824445193277
C 2.23390363304000 7.02475161850909 12.92700672851113
H 2.78771942681406 6.09033359412613 12.86371637563214
H 1.22659513210677 6.79985783908125 13.27182565264814
H 2.71073957638809 7.63764369890386 13.69246149010335
C 1.01872742048742 9.38081513448846 11.42885009930212
H 1.30391807398280 10.02483466615047 12.26160384336932
H -0.00336099763461 9.04588478926668 11.59481587387024
H 1.02363478782015 9.99004796155178 10.52683538068969
C 3.49876354452058 -0.13610685385136 10.37688775639335
H 3.46696206132243 -1.22618963373202 10.36231407258461
H 4.41589427278172 0.17686447264809 9.88156997106624
H 2.66049114438292 0.21904008456222 9.77848854372557

Table S14. Coordinates of the B3LYPHF30 fully geometry optimized structure of [La{P(SiMe$_3$)$_3$}$_2$(THF)$_2$] (**1-La**), in .xyz format with all coordinates in angstrom.

108
Coordinates from ORCA-job La_opt
La 2.03701731857698 3.66433503889142 9.74261371426283
P 0.74828582066808 2.20335719445233 7.58350212520888
P 3.40380286642380 2.80920275424702 12.19584984958593
P 1.82296689297361 6.48747205888001 9.70914221092863
Si 1.34032174422463 7.63651013361808 7.86985325124385
Si 0.72907707980570 0.03238016249558 7.10133105137267
Si -1.35293189135091 2.87585463842235 7.29161615259241
Si 2.13897507186721 7.94402819164721 11.35957038542453
Si 5.52443767178765 3.45766942049351 11.98812638284570
Si 3.49940882328343 0.59627852155961 12.01093054358432
O 0.01724790821844 3.21869166408572 11.23138152759412
O 4.10540390664880 3.69326848225728 8.21704152687823
C -1.60117162916205 4.47391291518814 8.25826164662082
H -0.88343498270871 5.22903174878587 7.95114942787498
H -1.48732944494817 4.34231683620136 9.33085572636345
H -2.59758952167026 4.87637105444076 8.07476851472352
C 2.47902271150571 -0.53824655286929 6.69619077558915
H 3.17379587551571 -0.37556286156924 7.51406348753705
H 2.85627010893943 -0.01583903841561 5.81868286641225
H 2.47558411605067 -1.60544481375403 6.47257223949297
C -2.67076744512054 1.64248016497730 7.84895966274568
H -3.65766460026209 2.09722333305829 7.75340979249010
H -2.54179218532332 1.33638392129278 8.88304451503315
H -2.66533992326922 0.74322164138528 7.23874754079585
C -0.42731459083963 8.28178184977942 7.83710965640638
H -0.61900080636173 8.81430885606573 6.90457044840917
H -0.60504696937938 8.97078443489673 8.65974344980748
H -1.15049321883680 7.47499417957274 7.92111317212935
C -0.11506190903058 3.93651680716867 12.47693623063551
H -0.90691628347250 4.67761470197232 12.36911132963091
H 0.82654723893579 4.43310828727767 12.67204090860827

C	5.42449012638856	4.64316163830508	6.51858980643692
H	6.34846086042379	5.17631023128271	6.31665360483059
H	4.66725281235021	4.98510023866803	5.81660316080275
C	2.47861371863072	9.12188032138691	7.64947930433770
H	3.52532392532954	8.82496652062090	7.66352663345024
H	2.33154988790537	9.85878638354871	8.43600798083819
H	2.27705458503856	9.61210558318165	6.69630612713525
C	4.95133392379575	4.84373868843366	7.94444728696625
H	5.77927824496865	4.83061879405997	8.64887139765890
H	4.35267967746575	5.73112241405062	8.11306614715539
C	1.59649066156564	6.48811231627355	6.39537235546912
H	1.23660794187244	6.95791135311678	5.47994635657695
H	1.07706601665775	5.53823874291322	6.50617947038453
H	2.65378722929815	6.26889999179823	6.26011443529055
C	-1.34370598272860	1.89404758642672	12.67701404998852
H	-2.39581426505192	2.10519719166694	12.84615751565675
H	-1.16516759364058	0.85853012880472	12.94589819616998
C	-0.45129223989311	2.85400408438594	13.48193872455471
H	0.46601591616772	2.36304487472028	13.79187511983307
H	-0.94705731022113	3.24946648278770	14.36308235914621
C	4.39133447155347	2.63028728968140	7.25938400799907
H	3.49771668034251	2.49130758212175	6.65968139726397
H	4.59226938662005	1.72284748581605	7.81751624071204
C	-1.68340232222703	3.25887594076635	5.47876056382994
H	-2.70712179342448	3.60550607677707	5.33139814778474
H	-1.53150114558548	2.37919012176048	4.85749845502729
H	-1.00722915139270	4.03446767557542	5.12408423988553
C	0.07936484117098	-1.01295624103727	8.52994826967825
H	-0.94173866148860	-0.73326075826808	8.78083455223192
H	0.69169163349251	-0.88058438735638	9.41975545749327
H	0.08369573231578	-2.07343415568459	8.27516515302153
C	6.48110844497266	3.20922763957053	13.58994033484278
H	6.55356558920610	2.15729530811413	13.85523536273017
H	5.98611512553518	3.72445507539390	14.41102008511227
H	7.49360305519587	3.60500589031599	13.50089319312404
C	3.80087621094528	8.81616265813156	11.23055217973968
H	3.92953419854469	9.51769698494476	12.05591656824815
H	3.88008489708618	9.37313558758820	10.30007917200416
H	4.61887008468831	8.10108733985121	11.26295568754801
C	5.57741027204724	3.12243109732142	6.44479319180869
H	5.55274115351571	2.73822367328520	5.42997775431448
H	6.51401642787557	2.81448748138451	6.90437112985372
C	-0.97963904918529	2.17094965795965	11.21053007959262
H	-0.54462272915750	1.32828085500165	10.68815636623462
H	-1.83243163175675	2.52698952970390	10.64170558248018
C	-0.30212908311877	-0.34578645297856	5.56932159761419
H	-0.22764694118773	-1.40719235584345	5.33007312104899
H	0.06532732870970	0.21799984142451	4.71410678167029
H	-1.35472922289536	-0.10995430941389	5.69638917036008
C	5.50675203980287	5.31012976102021	11.63458462373878
H	6.51271090881292	5.67674422691602	11.42870744935075
H	5.13183640812130	5.84776422887638	12.50326701678749
H	4.87085212203787	5.57828469240272	10.79418887742230
C	6.49523095404315	2.58863274643961	10.62007701411243
H	7.48710543504470	3.03084571233358	10.51938227740647
H	5.98733665573428	2.66695000096181	9.66404599960651
H	6.62835503697203	1.53191494756196	10.84086792460464

C	1.85987895828774	-0.12142452416955	12.60444894339065
H	1.86483119747733	-1.20828166955110	12.52272751885444
H	1.03091537417431	0.24849707575232	12.00602931119606
H	1.66761012664100	0.13875797578358	13.64322610560002
C	4.87626083557224	-0.19013971636224	13.02411159097249
H	4.80301317648322	0.10068281446308	14.07005693425960
H	5.86080695121671	0.10047757161993	12.66562189432665
H	4.80970633841024	-1.27710770872980	12.96806269231475
C	2.07400283110355	7.02131346316222	13.00007630689269
H	2.70403181403087	6.13448289058406	13.00223578722985
H	1.05497562100191	6.71367936614986	13.22582799864448
H	2.40911403334090	7.67067273562009	13.80898106151003
C	0.79111428437584	9.25952672427591	11.38218556547169
H	0.91221345949493	9.90913328110864	12.24985709969281
H	-0.19563686359760	8.80383775024876	11.43272621639599
H	0.82336258506144	9.88662684923124	10.49392081834742
C	3.70399036127540	0.03343120868995	10.22025490904275
H	3.67735809744021	-1.05306500868936	10.13383497591911
H	4.64624032035739	0.38103309839716	9.80392267459988
H	2.89718924487181	0.42007808105761	9.59853642354223

Table S15. Experimental and calculated ^{31}P chemical shifts and anisotropy parameters for **1-La**, using both XRD and optimized structures with different DFT methods. The optimization in each case was carried out using the same method as the NMR parameter calculation.

Method	Structure	δ_{iso} (ppm)	δ_{11} (ppm)	δ_{22} (ppm)	δ_{33} (ppm)	Ω (ppm)	κ
Experimental	-	-123 (solid), -113.0 (solution)	32	-139	-262	295	-0.16
BP86	XRD	-77.7	288.2	-197.0	-324.4	612.5	-0.58
	XRD (PCM)	-84.6	271.5	-198.7	-326.5	598.0	-0.57
	Optimized (H only)	-96.6	247.5	-190.4	-346.9	594.5	-0.47
	Optimized (H only, PCM)	-108.3	222.0	-191.1	-355.8	577.8	-0.43
	Optimized	-72.4	357.0	-201.6	-372.6	729.7	-0.53
	Optimized (PCM)	-78.1	318.9	-211.1	-342.1	661.1	-0.60
B3LYPHF20	XRD	-85.6	270.5	-200.4	-326.8	597.3	-0.58
	XRD (PCM)	-92.4	252.8	-200.3	-329.7	582.6	-0.56
	Optimized (H only)	-109.0	224.3	-203.3	-348.1	572.4	-0.49
	Optimized (H only, PCM)	-119.3	200.7	-204.3	-354.2	554.9	-0.46
	Optimized	-66.5	283.3	-100.9	-382.1	665.4	-0.15
	Optimized (PCM)	-41.5	303.3	-56.3	-371.7	675.0	-0.07
B3LYPHF25	XRD	-91.6	257.1	-203.3	-328.7	585.8	-0.57
	XRD (PCM)	-98.3	239.6	-202.9	-331.6	571.2	-0.55
	Optimized (H only)	-115.1	212.2	-208.7	-348.8	561.1	-0.50
	Optimized (H only, PCM)	-125.0	189.1	-209.6	-354.7	543.8	-0.47
	Optimized	-64.0	282.4	-71.5	-403.0	685.4	-0.03
	Optimized (PCM)	-68.0	235.5	-85.2	-354.2	589.6	-0.09
B3LYPHF30	XRD	-97.5	243.7	-206.1	-330.2	573.9	-0.57
	XRD (PCM)	-104.0	226.6	-205.3	-333.2	559.8	-0.54
	Optimized (H only)	-121.0	200.4	-213.9	-349.5	549.8	-0.51
	Optimized (H only, PCM)	-130.6	177.8	-214.7	-355.0	532.8	-0.47
	Optimized	-56.5	227.2	-75.2	-321.6	548.8	-0.10
	Optimized (PCM)	-85.1	196.9	-92.4	-360.0	556.9	-0.04
B3LYPHF35	XRD	-103.4	230.5	-208.8	-331.8	562.3	-0.56
	XRD (PCM)	-109.6	213.7	-207.7	-334.8	548.5	-0.54

	Optimized (H only)	-126.8	188.6	-218.9	-350.0	538.6	-0.51
	Optimized (H only, PCM)	-136.1	166.4	-219.7	-355.1	521.5	-0.48
	Optimized	-80.9	202.4	-102.6	-342.4	544.8	-0.12
	Optimized (PCM)	-101.3	160.2	-94.1	-369.9	530.1	0.04
B3LYPHF40	XRD	-108.9	217.7	-211.3	-333.1	550.7	-0.56
	XRD (PCM)	-115.0	201.1	-209.8	-336.2	537.3	-0.53
	Optimized (H only)	-132.3	176.9	-223.6	-350.2	527.1	-0.52
	Optimized (H only, PCM)	-142.0	154.6	-224.0	-356.6	511.2	-0.48
	Optimized	-101.5	174.4	-123.5	-355.4	529.8	-0.12
	Optimized (PCM)	-137.7	82.7	-132.1	-363.9	446.5	0.04
B3LYPHF45	XRD	-114.3	205.0	-213.7	-334.3	539.3	-0.55
	XRD (PCM)	-120.1	188.8	-211.8	-337.4	526.2	-0.52
	Optimized (H only)	-138.4	164.8	-227.80	-352.2	517.0	-0.52
	Optimized (H only, PCM)	-147.1	143.7	-228.8	-356.3	500.0	-0.49
	Optimized	-124.2	145.1	-95.0	-422.6	567.7	0.15
	Optimized (PCM)	-134.2	123.0	-104.6	-421.2	544.3	0.16
B3LYPHF50	XRD	-119.6	192.5	-215.9	-335.5	528.0	-0.55
	XRD (PCM)	-125.2	176.7	-213.6	-338.6	515.3	-0.52
	Optimized (H only)	-143.5	153.7	-232.2	-352.0	505.6	-0.53
	Optimized (H only, PCM)	-152.0	133.0	-233.3	-355.8	488.8	-0.50
	Optimized	-171.4	111.3	-127.0	-498.6	609.9	0.22
	Optimized (PCM)	-208.1	150.2	-175.3	-599.3	749.5	0.13

Table S16. Mayer bond order and NBO analysis of the P-La bonds in **1-La**. Contributions from d and f orbitals on phosphorus are not shown as they are always extremely small. Phosphorus atoms are ordered by increasing pyramidalization.

Method	Structure	Phosphorus	Mayer bond order	σ NBO				π NBO			
				%La	%s/p/d/f	%P	%s/p	%La	%s/p/d/f	%P	%s/p
B3LYPHF20	XRD	P(1)	0.7928	7.58	22.85/2.03/69.67/5.45	92.42	50.39/49.55	8.45	0.15/0.59/68.52/30.75	91.55	0.04/99.88
		P(2)	0.7545	6.95	17.82/1.31/67.66/13.21	93.05	52.47/47.43	7.68	0.86/0.73/58.59/39.82	92.32	0.30/99.61
		P(3)	0.7346	7.00	17.88/0.50/50.65/30.97	93.00	53.92/45.77	7.05	6.84/1.18/65.64/26.34	92.95	0.50/99.21
	Optimized (H only)	P(1)	0.8001	7.11	22.16/2.3/69.67/5.87	92.89	50.48/49.45	8.24	0.17/0.59/67.81/31.43	91.76	0.02/99.90
		P(2)	0.7396	6.55	16.68/1.48/68.19/13.65	93.45	52.23/47.67	7.49	0.56/0.69/58.07/40.68	92.51	0.36/99.55
		P(3)	0.7209	6.78	17.59/0.55/51.22/30.65	93.22	53.99/45.71	6.69	5.20/1.25/66.23/27.33	93.31	0.63/99.08
	Optimized	P(1)	0.8366	6.97	21.72/2.09/69.36/6.83	93.03	51.74/48.15	8.00	0.53/0.96/67.99/30.52	92.00	0.24/99.64
		P(2)	0.7637	6.39	15.16/1.49/67.22/16.13	93.61	54.00/45.87	7.59	0.87/0.86/60.86/37.40	92.41	0.44/99.46
		P(3)	0.6893	6.57	15.05/0.46/49.89/34.60	93.43	56.00/43.66	6.16	4.13/1.21/72.30/22.36	93.84	0.46/99.11
B3LYPHF30	XRD	P(1)	0.7627	7.28	22.92/1.96/69.51/5.61	92.72	50.86/49.07	8.02	0.14/0.57/67.19/32.10	91.98	0.05/99.88
		P(2)	0.7336	6.69	17.89/1.29/67.67/13.16	93.31	53.06/46.83	7.32	0.80/0.70/57.64/40.86	92.68	0.33/99.57
		P(3)	0.7151	6.83	18.04/0.50/50.92/30.53	93.17	54.64/45.04	6.67	6.61/1.10/65.16/27.13	93.33	0.57/99.13
	Optimized (H only)	P(1)	0.7692	6.83	22.23/2.22/69.54/6.01	93.17	51.06/48.87	7.82	0.16/0.58/66.44/32.83	92.18	0.03/99.89
		P(2)	0.7178	6.32	16.76/1.45/68.18/13.61	93.68	52.94/46.95	7.14	0.52/0.66/57.14/41.69	92.86	0.40/99.51
		P(3)	0.7018	6.60	17.71/0.55/51.45/30.28	93.40	54.80/44.88	6.35	5.03/1.17/65.80/28.00	93.65	0.69/99.01
	Optimized	P(1)	0.7713	6.68	23.08/2.37/69.20/5.34	93.32	51.34/48.59	7.97	0.00/0.44/69.14/30.42	92.03	0.02/99.91
		P(2)	0.7292	6.02	15.23/1.23/66.63/16.91	93.98	54.15/45.71	6.97	0.64/0.91/59.13/39.32	93.03	0.58/99.31
		P(3)	0.6834	6.36	15.93/0.52/50.20/33.36	93.64	55.26/44.39	6.13	4.42/1.18/70.55/23.85	93.87	0.61/99.01

11. Complete Active Space Self-Consistent Field (CASSCF) Calculations

Table S17. Electronic structure of **1-Ce** obtained from CASSCF-SO using the solid state geometry of **1-Ce**. Each row corresponds to a Kramers doublet.

**Energy (cm^{-1})**	**Energy (K)**	g_x	g_y	g_z	**Angle[a] (deg)**	**Wavefunction[b]**	**$\langle J_z \rangle$**
0	0	0.1917	0.4003	3.4980	-	78%$\|\pm 5/2\rangle$ + 18%$\|\pm 1/2\rangle$ + 3%$\|\mp 1/2\rangle$ + 3%$\|\pm 3/2\rangle$	±2.062
163	235	0.3983	0.6373	3.8749	59	57%$\|\pm 3/2\rangle$ + 18%$\|\pm 1/2\rangle$ + 14%$\|\pm 5/2\rangle$ + 9%$\|\mp 1/2\rangle$	±1.242
557	802	0.5499	1.7285	3.2414	68	43%$\|\pm 1/2\rangle$ + 37%$\|\pm 3/2\rangle$ + 11%$\|\mp 1/2\rangle$ + 7%$\|\pm 5/2\rangle$	±0.858

[a] The angle between the g_z value of the excited Kramers doublet and the ground Kramers doublet. [b] Contributions > 2%.

Table S18. Electronic structure of **1-Pr** obtained from CASSCF-SO using the solid state geometry of **1-Pr**. States are singly degenerate.

**Energy (cm^{-1})**	**Energy (K)**	**Wavefunction**[b]
0	0	48%|+4⟩ + 48%|−4⟩
0.94	1.35	49%|+4⟩ + 49%|−4⟩
184	265	86%|0⟩ + 5%|+1⟩ + 5%|−1⟩
256	369	31%|+1⟩ + 31%|−1⟩ + 11%|+3⟩ + 11%|−3⟩ + 5%|0⟩ + 4%|+2⟩ + 4%|−2⟩
323	464	40%|+3⟩ + 40%|−3⟩ + 7%|+1⟩ + 7%|−1⟩ + 3%|+2⟩ + 3%|−2⟩
343	493	35%|+1⟩ + 35%|−1⟩ + 11%|+3⟩ + 11%|−3⟩ + 4%|+2⟩ + 4%|−2⟩
395	569	34%|+3⟩ + 34%|−3⟩ + 8%|+1⟩ + 8%|−1⟩+ 6%|0⟩ + 4%|+2⟩ + 4%|−2⟩
514	739	41%|+2⟩ + 41%|−2⟩ + 7%|+1⟩ + 7%|−1⟩
546	785	43%|+2⟩ + 43%|−2⟩ + 5%|+1⟩ + 5%|−1⟩

[a] The angle between the g_z value of the excited Kramers doublet and the ground Kramers doublet. [b] Contributions > 2%.

Table S19. Electronic structure of **1-Nd** obtained from CASSCF-SO using the solid state geometry of **1-Nd**. Each row corresponds to a Kramers doublet.

Energy (cm^{-1})	Energy (K)	g_x	g_y	g_z	Angle[a] (deg)	Wavefunction[b]	$<J_z>$
0	0	0.3680	0.4514	5.8743	-	89%|± 9/2⟩ + 6%|± 5/2⟩ + 5%|∓ 3/2⟩	4.067
105	152	0.4817	2.2344	4.205	81	37%|± 5/2⟩ + 32%|± 3/2⟩ + 14%|∓ 1/2⟩ + 5%|∓ 3/2⟩ + 4%|∓ 5/2⟩ + 3%|∓ 7/2⟩	1.138
152	219	0.4569	1.0841	3.1371	60	31%|± 5/2⟩ + 37%|± 3/2⟩ + 11%|∓ 3/2⟩ + 6%|± 9/2⟩ + 5%|∓ 1/2⟩ + 3%|∓ 7/2⟩ + 2%|∓ 9/2⟩	1.230
233	336	0.2612	1.5708	4.4471	90	45%|± 1/2⟩ + 29%|∓ 1/2⟩ + 10%|±5/2⟩ + 6%|±3/2⟩ + 4%|∓ 5/2⟩ + 3%|∓ 3/2⟩	0.273
333	479	1.2645	1.6648	4.3724	8	87%|± 7/2⟩ + 7%|∓5/2⟩ + 3%|± 1/2⟩	2.940

[a] The angle between the g_z value of the excited Kramers doublet and the ground Kramers doublet. [b] Contributions > 2%.

Table S20. Electronic structure of **1-Sm** calculated with the crystal field parameters obtained from CASSCF-SO using the solid state geometry of **1-Sm** in zero-field. Each row corresponds to a Kramers doublet.

**Energy (cm^{-1})**	**Energy (K)**	g_x	g_y	g_z	**Angle[a] (deg)**	**Wavefunction[b]**	**$\langle J_z \rangle$**
0	0	0.1446	0.3282	1.0709	-	87%$\|\pm 5/2\rangle$ + 5%$\|\pm 1/2\rangle$ + 4%$\|\mp 1/2\rangle$ + 3%$\|\pm 3/2\rangle$	±2.221
114	164	0.3005	0.4019	1.0273	87	51%$\|\pm 3/2\rangle$ + 37%$\|\pm 1/2\rangle$ + 8%$\|\pm 5/2\rangle$ + 3%$\|\mp 5/2\rangle$	±1.073
257	370	0.0841	0.2783	1.8651	80	53%$\|\pm 1/2\rangle$ + 44%$\|\pm 3/2\rangle$	±0.916

[a] The angle between the g_z value of the excited Kramers doublet and the ground Kramers doublet. [b] Contributions > 2%.

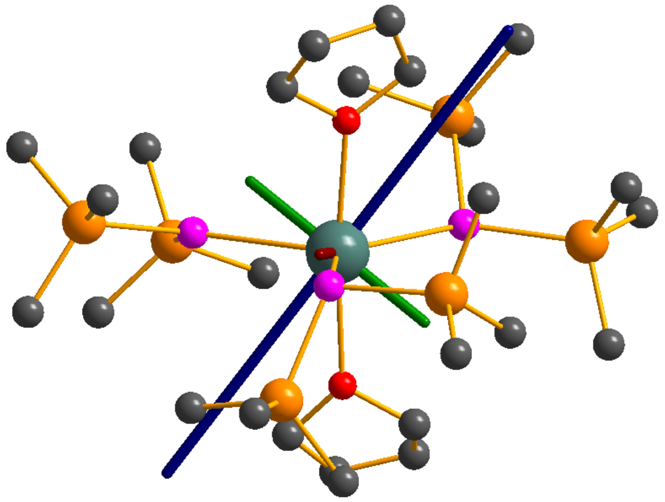

Figure S62. CASSCF-calculated magnetic axes for ground doublet of **1-Ce** (blue: g_z, easy; green: g_y, intermediate; red: g_x, hard). Lanthanide, phosphorus, silicon, oxygen and carbon shown as metallic green, magenta, orange, red and gray respectively. Hydrogen atoms omitted for clarity.

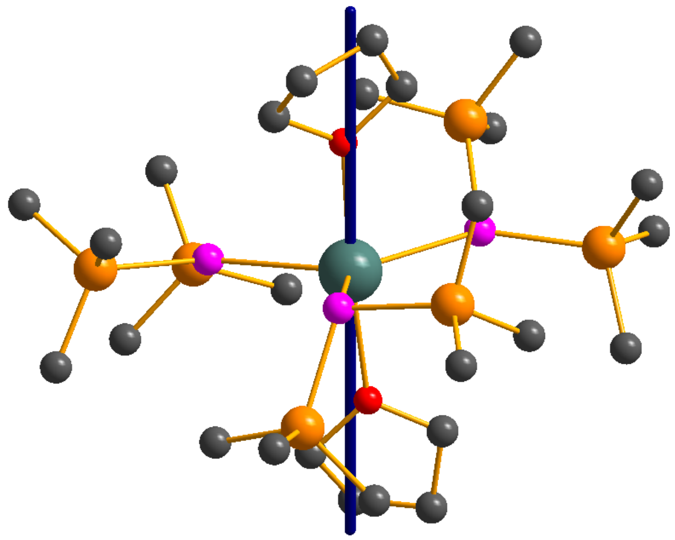

Figure S63. CASSCF-calculated magnetic axes for ground pseudo-doublet of **1-Pr** (blue: g_z, easy). Lanthanide, phosphorus, silicon, oxygen and carbon shown as metallic green, magenta, orange, red and gray respectively. Hydrogen atoms omitted for clarity.

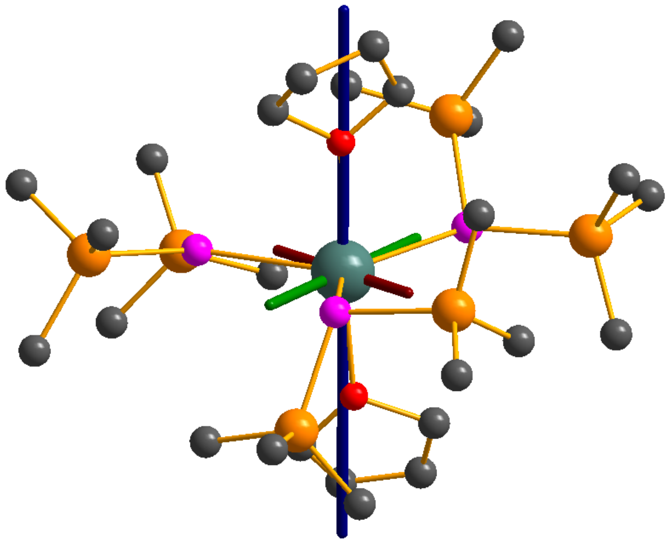

Figure S64. CASSCF-calculated magnetic axes for ground pseudo-doublet of **1-Nd** (blue: g_z, easy). Lanthanide, phosphorus, silicon, oxygen and carbon shown as metallic green, magenta, orange, red and gray respectively. Hydrogen atoms omitted for clarity.

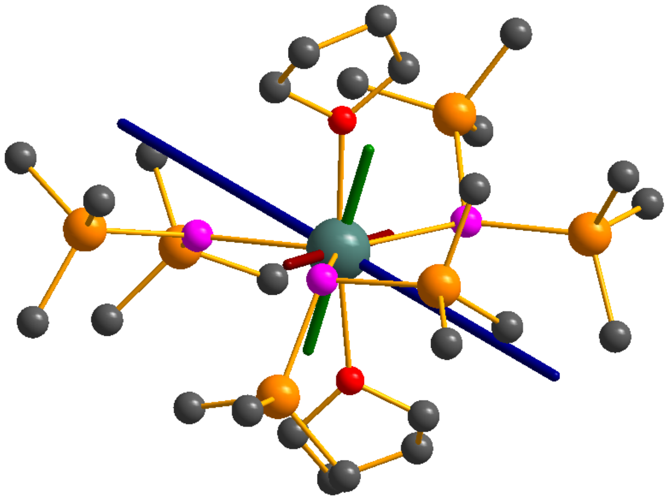

Figure S65. CASSCF-calculated magnetic axes for ground pseudo-doublet of **1-Sm** (blue: g_z, easy). Lanthanide, phosphorus, silicon, oxygen and carbon shown as metallic green, magenta, orange, red and gray respectively. Hydrogen atoms omitted for clarity.

12. pNMR calculations

Table S21. Calculated pNMR shifts for paramagnetic **1-Ln**, found using the point-dipole limit magnetic susceptibility form of the pseudocontact shift.

Nucleus	Complex	Environment	Experimental Paramagnetic Shift (ppm)	Calculated Paramagnetic Shift (ppm)		
				XRD Structure and Vacuum Calculation	Optimized Structure and Vacuum Calculation	Optimized Structure and PCM Calculation
^{1}H	**1-Ce**	CH_3	-2.17	-2.34	-2.09	-2.16
		THF-β	1.02	8.01	8.15	8.30
		THF-α	8.33	15.30	11.72	11.91
	**1-Pr**	CH_3	-6.83	-10.16	-5.52	-5.71
		THF-β	23.50	32.71	18.28	18.76
		THF-α	48.12	61.38	32.29	33.07
	**1-Nd**	CH_3	-2.24	-3.87	-2.03	-2.11
		THF-β	11.75	12.07	7.29	7.56
		THF-α	23.52	21.91	10.31	10.74
	**1-Sm**	CH_3	0.09	-0.54	-0.33	-0.35
		THF-β	2.35	1.69	1.37	1.43
		THF-α	6.11	2.53	1.22	1.30
^{13}C	**1-Ce**	CH_3	7.93	-2.90	-2.60	-2.68
		THF-β	-	12.05	11.84	12.04
		THF-α	-	24.96	20.64	20.95
	**1-Pr**	CH_3	11.17	-12.64	-7.04	-7.26
		THF-β	-	48.50	27.78	28.48
		THF-α	-	100.18	54.38	55.72
	**1-Nd**	CH_3	23.94	-4.87	-2.55	-2.67
		THF-β	-	18.41	10.80	11.20
		THF-α	-	37.15	18.80	19.53
	**1-Sm**	CH_3	5.47	-0.65	-0.37	-0.40
		THF-β	26.15	2.47	1.92	2.00
		THF-α	78.89	4.61	2.86	3.00
^{29}Si	**1-Ce**	$SiMe_3$	5.30	-5.74	-4.77	-4.91
	**1-Pr**	$SiMe_3$	15.65	-26.19	-14.22	-14.64
	**1-Nd**	$SiMe_3$	42.94	-9.84	-4.81	-5.03
	**1-Sm**	$SiMe_3$	0.52	-1.14	-0.56	-0.60
^{31}P	**1-Ce**	P-Ln	616.7	-27.2	-20.0	-20.1
	**1-Pr**	P-Ln	1894.2	-107.8	-56.7	-57.9
	**1-Nd**	P-Ln	2570.1	-40.8	-18.6	-19.3
	**1-Sm**	P-Ln	-259.2	-4.4	-2.6	-2.7

Table S22. Calculated pNMR shifts for some of **1-Ln**, found by applying the van den Heuvel equation to the results of RASCI-SO calculations. Atomic orbitals used in RAS1 specifications refer to those of all three phosphorus atoms.

Nucleus	Complex	Environment	Experimental Paramagnetic Shift (ppm)	Calculated Paramagnetic Shift (ppm)							
				CASSCF-SO	RAS1 = 1s	RAS1 = 2s	RAS1 = 3s	RAS1 = 1s, 2s, 3s	RAS1 = 1s, 2s, 3s, 3×3p	RAS1 = 1s, 2s, 3s, 6×3p	RAS1 = 1s, 2s, 3s, 9×3p
^{1}H	**1-Ce**	CH_3	-2.17	2.39							-2.70
		THF-β	1.02	8.09							9.42
		THF-α	8.33	15.12							17.72
	**1-Pr**	CH_3	-6.83	-10.13							-11.95
		THF-β	23.50	32.59							37.41
		THF-α	48.12	60.75							69.77
	**1-Nd**	CH_3	-2.24	-3.92							-4.51
		THF-β	11.75	12.02							14.54
		THF-α	23.52	21.12							25.89
	**1-Sm**	CH_3	0.09	-0.53							-0.52
		THF-β	2.35	1.68							1.70
		THF-α	6.11	2.52							2.54
^{13}C	**1-Ce**	CH_3	7.93	-6.51							-2.39
		THF-β	-	10.41							-9.94
		THF-α	-	25.48							25.93
	**1-Pr**	CH_3	11.17	-13.31							-10.88
		THF-β	-	47.82							54.83
		THF-α	-	95.78							105.23
	**1-Nd**	CH_3	23.94	-8.22							-0.36
		THF-β	-	15.20							21.29
		THF-α	-	27.24							30.96
	**1-Sm**	CH_3	5.47	-0.58							-1.08
		THF-β	26.15	2.57							2.50
		THF-α	78.89	5.11							5.25
^{29}Si	**1-Ce**	$SiMe_3$	5.30	-9.94							-1.93
	**1-Pr**	$SiMe_3$	15.65	-35.12							-15.27
	**1-Nd**	$SiMe_3$	42.94	-20.09							4.88
	**1-Sm**	$SiMe_3$	0.52	-0.46							-1.36
^{31}P	**1-Ce**	P-Ln	616.7	-52.7	-52.6	-52.6	-48.5	-48.5	-39.1	-3.2	-1.5
	**1-Pr**	P-Ln	1894.2	-201.3	-201.6	-201.6	-190.8	-109.8	-177.0	-103.0	-96.0
	**1-Nd**	P-Ln	2570.1	-172.8	-172.7	-172.7	-160.0	-160.0	-143.0	146.6	-40.6
	**1-Sm**	P-Ln	-259.2	-0.5	-0.6	-0.6	-1.5	-1.5	-4.4	-6.2	-6.1

Table S23. Calculated ^{31}P pNMR shifts for some of **1-Ln**, found by applying the van den Heuvel equation to the results of various multi-configurational electronic structure theory calculations.

Nucleus	Complex	Environment	Experimental Paramagnetic Shift (ppm)	Calculated Paramagnetic Shift (ppm)	
				RASSCF-SO	RASCI-SO
^{31}P	**1-Ce**	P–Ln	616.7	-42.9	-
	**1-Pr**	P–Ln	1894.2	-193.1	-
	**1-Nd**	P–Ln	2570.1	-106.8	-89.2
	**1-Sm**	P–Ln	-259.2	-1.1	-

13. References

1. *WSolids1 ver. 1.21.7*, Eichele, K., Universität Tübingen, 2021.

2. Vold, R. L.; Hoatson, G. L. Effects of jump dynamics on solid state nuclear magnetic resonance line shapes and spin relaxation times. *J Magn Reson.* **2009**, *198*, 57–72.

3. Young, R. P.; Lewis, C. R.; Yang, C.; Wang, L.; Harper, J. K.; Mueller, L. J. TensorView: A Software Tool for Displaying NMR Tensors, *Magn. Reson. Chem.* **2019**, *57*, 211–223.
